# Exploring The Causal Relationship Between Lipid Profiles and Colorectal Cancer Through Mendelian Randomization: A Multidimensional Plasma Lipid Composition Perspective

**DOI:** 10.7150/jca.103247

**Published:** 2025-02-28

**Authors:** Hailan Wu, Jialin Gu, Yun He, Yi Ji, Wen Cao, Rongrong Li, Zhancheng Gu, Guoli Wei, Jiege Huo

**Affiliations:** 1 Affiliated Hospital of Integrated Traditional Chinese and Western Medicine, Nanjing University of Chinese Medicine, Nanjing 210028, Jiangsu, China.; 2 Jinling Hospital, Affiliated Hospital of Medical School, Nanjing University, Nanjing 210002, Jiangsu 210022, Jiangsu, China.; 3 Department of Traditional Chinese medicine, The First Affiliated Hospital, Zhejiang University School of Medicine, Hangzhou 310013, Zhejiang, China.; 4 Department of Oncology, Changshu Hospital Affiliated to Nanjing University of Chinese Medicine, Changshu 215500, Jiangsu, China.; 5 Jiangsu Cancer Hospital, Nanjing 210009, Jiangsu, China.; 6 Department of Oncology, Kunshan Hospital of Traditional Chinese Medicine, Suzhou 215399, Jiangsu, China.; Hailan Wu and Jialin Gu are co-first authors.

**Keywords:** Lipid, Colorectal cancer, Causal association, Mendelian randomization, 179 lipid species

## Abstract

**Background:** The causal relationship between blood lipids and colorectal cancer (CRC) risk has been preliminarily explored in previous Mendelian randomization (MR) studies, but these investigations were limited to conventional or partial metabolic lipid profiles. Recent advancements in genome-wide association studies of plasma lipidomics have expanded our understanding of lipid categories, underscoring the need to evaluate the causal associations between a broader range of lipid types and CRC risk to enhance risk assessment.

**Methods:** This MR study utilized 179 lipid phenotypes across 13 lipid classes to investigate their causal associations with CRC risk. Genetic variants significantly associated with lipid traits at the genome-wide level (*P*<5×10^-8^) were used as instrumental variables for MR analysis. Initial analyses were conducted using a discovery dataset (n=321,040), followed by validation in an independent replication dataset (n=185,616). Meta-analysis was then employed to determine the strength of causal evidence. The inverse-variance weighted (IVW) method and Wald ratio were the primary MR approaches, complemented by up to nine methods for multidimensional validation. Sensitivity analyses included tests for pleiotropy, heterogeneity, Steiger directionality, and Bayesian colocalization analysis, among others.

**Results:** After Bonferroni correction and rigorous validations, 9 significant causal associations were identified. Specifically, genetically predicted levels of sterol ester (27:1/20:5) (OR_IVW_ = 1.214, 95% CI 1.119-1.317), phosphatidylcholine (20:4_0:0) (OR_IVW_ = 1.147, 95% CI 1.077-1.222), phosphatidylcholine (16:0_22:4) (OR_IVW_ = 1.312, 95% CI 1.170-1.472), phosphatidylcholine (16:0_22:5) (OR_IVW_ =1.181, 95% CI 1.093-1.277), and phosphatidylcholine (18:0_20:5) (OR_IVW_ = 1.198, 95% CI 1.104-1.300) were significantly associated with an increased risk of CRC. Conversely, levels of phosphatidylcholine (18:1_20:2) (OR_IVW_ = 0.832, 95% CI 0.771-0.898), phosphatidylethanolamine (18:2_0:0) (OR_IVW_ = 0.804, 95% CI 0.732-0.882), phosphatidylcholine (16:0_18:0) (OR_Wald ratio_ = 0.611, 95% CI 0.481-0.777), and phosphatidylcholine (O-18:1_18:2) (OR_Wald ratio_ = 0.723, 95% CI 0.620-0.840) were significantly associated with a decreased risk of CRC. Colocalization analysis revealed posterior probabilities for hypothesis 4 exceeding 90%, identifying rs174546 and rs28456 as shared causal variants. Additionally, 14 suggestive causal associations were observed.

**Conclusions:** This study establishes a causal link between specific lipid species and CRC risk. These findings suggest new avenues for CRC prevention and treatment strategies.

## Introduction

Colorectal cancer (CRC), primarily affecting the mucosal layer of the colon or rectum, ranks among the most common malignant tumors within the digestive system^1^. By 2020, global statistics revealed over 1.9 million new cases of CRC and more than 930,000 deaths, accounting for 10.0% of all new cancer cases. This ranks CRC as the third most common cancer worldwide and the second leading cause of cancer-related mortality, trailing only breast and lung cancers^2^. The annual incidence and mortality rates of CRC remain high, with projections indicating an increase to 3.2 million new cases (a 63% increase) and 1.6 million deaths (a 73% increase) by 2040 2. The pathogenesis of CRC is complex, involving a multitude of factors including genetics, lifestyle, socioeconomic status, geographical differences, and a variety of modifiable environmental risks^3^. Despite some progress in the diagnosis and treatment of CRC in recent years, prevention remains challenging, partly due to the incomplete understanding of its etiology, hindering the implementation of effective preventive measures. Most CRC patients are diagnosed at advanced stages due to the absence of early symptoms or subtle clinical signs^4^, imposing significant economic burdens on patients, complicating treatment, reducing survival rates, and severely impacting their quality of life. Therefore, understanding in depth the etiopathology of CRC and its risk factors becomes of prime importance toward improving early diagnosis rates, designing effective strategies for prevention, and thereby reducing the burden of CRC incidence.

Lipid biomarkers have significant roles not only as indicators of the status of lipid metabolism in the body but also as important tools with help in the evaluation of the risk of cerebrovascular diseases and other metabolic disorders. It has been shown to have a significant role in the inflammatory pathways, oxidative stress, and insulin resistance that probably account for the appearance of CRC^5^. Traditional lipid biomarkers have levels of low-density lipoprotein cholesterol (LDL-C), high-density lipoprotein cholesterol (HDL-C), triglycerides (TG), and total cholesterol (TC) and have been used for a long time in CRC assessment and management. However, due to the inherent limitations of observational studies, the results remain controversial^6^-^11^. Mendelian randomization (MR) analysis, an epidemiological approach that uses genetic variants as instrumental variables (IVs), addresses several challenges faced by randomized controlled trials (RCTs) and offers a powerful alternative for establishing causal relationships. The basic core principle of MR analysis lies in the truth that there is a random allocation of genetic variants at conception. Thus, MR analysis effectively avoids confounding and reverse causation. Although published MR studies have provided a new perspective in addressing these issues, their analyses have primarily focused on common lipid phenotypes, limiting our understanding of the depth and breadth of the relationship between lipids and CRC^12^-^15^.

Recently, research conducted by Ottensmann *et al.* has significantly expanded our understanding of plasma lipid varieties through a comprehensive analysis of a broader range of lipid types^16^. This study goes beyond standard lipid measurements, elucidating the importance of precisely identifying various lipid categories, thereby opening new perspectives on the potential of lipids as biomarkers for disease risk. Compared to relying solely on traditional lipid biomarkers, the investigation of a wider array of lipid types offers more detailed and comprehensive information for predicting CRC risk. This approach facilitates the advancement of personalized medicine in CRC risk stratification and therapeutic intervention guidance. Therefore, we employ MR analysis to explore the causal association between multidimensional lipid phenotypes and CRC, providing more precise and comprehensive information for the risk assessment and management of CRC.

## Materials and Methods

### Study design

**Figure 1:**
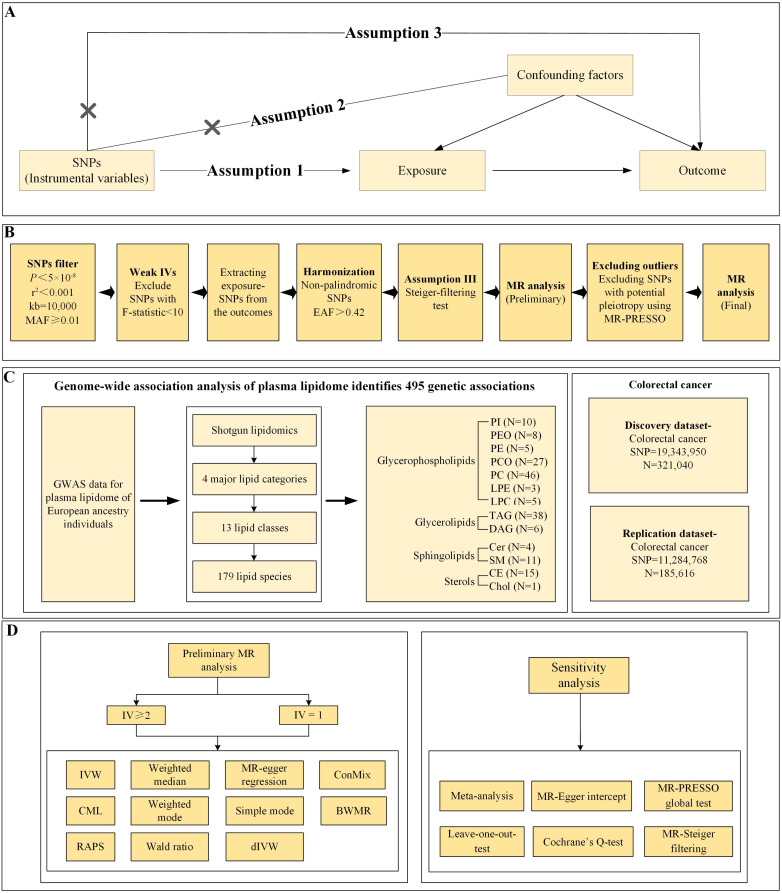
** Study design** A) Mendelian Randomization Analysis Three Key Assumptions: Relevance, Exclusion Restriction, and Independence Assumptions B) Detailed Instrumental Variable Selection Process C) Utilization of GWAS Data Sources D) Primary MR Analysis and Sensitivity Analysis Methods. PI, Phosphatidylinositol; PEO, Phosphatidylethanolamine-ether; PE, Phosphatidylethanolamine; PCO, Phosphatidylcholine-ether; PC, Phosphatidylcholine; LPE, Lysophosphatidylethanolamine; LPC, Lysophosphatidylcholine; TAG, Triacylglycerols; DAG, Diacylglycerols; Cer, Ceramide; SM, Sphingomyelin; CE, Cholesteryl ester; Chol, Cholesterol; GWAS, Genome-wide association studies; MR, Mendelian randomization; MR-PRESSO, MR Pleiotropy Residual Sum and Outlier; IV, instrumental variable; IVW, inverse-variance-weighted; RAPS, robust adjusted profile score; CML, constrained maximum likelihood; dIVW, debiased inverse-variance weighted; ConMix, contamination mixture; SNP, single nucleotide polymorphism; EAF, effect allele frequency; MAF, minor allele frequency; BWMR, Bayesian weighted Mendelian randomization.

This study employs secondary analysis on publicly available and shared genome-wide association studies (GWAS) data, thus obviating the need for ethical approval or clinical registration and adhering to Strengthening the Reporting of Observational Studies in Epidemiology using Mendelian Randomization (STROBE-MR) reporting guidelines^17^. It explores the potential causal relationships between 179 designated plasma lipidome species and CRC via MR analysis. The selection of IVs is predicated on the three core assumptions of MR: (i) The relevance assumption stipulates that genetic variants employed as instrumental variables must exhibit a robust association with the exposure; (ii) The independence assumption demands that genetic variants remain uncorrelated with any confounders that influence the outcome; (iii) The exclusion restriction assumption requires that the impact of genetic variants on the outcome be channeled solely through the exposure, precluding any alternative pathways^18^. **Figure 1** delineates the specifics of the study design.

### Instrumental variables selection criteria

(i) Preliminary screening of single nucleotide polymorphism (SNP) within the exposure GWAS dataset was conducted at genome-wide significance levels (*P*<5×10^-8^) and under stringent linkage disequilibrium (LD) clumping (Phase 3 of 1,000 Genomes: r^2^ < 0.001, clumping distance = 10MB). Additionally, we implemented a quality control threshold of a minor allele frequency (MAF) of 0.01 to ensure sufficient statistical power and reliability in the MR analysis.

(ii) The F-statistic for each SNP was calculated, and any SNP with an F-statistic [*F*=((*n*-*k*-1)/*k*)( *R^2^*/(1-* R^2^*)) less than 10 was excluded as a weak IV^19^. R² [R²=2×minor allele frequency (MAF)×(1-MAF)×beta²] quantifies the proportion of variance in the exposure attributable to the SNPs, N represents the sample size in the GWAS, and k = 1 indicates analysis based on individual SNPs^20^,^21^.

(iii) SNPs were further extracted from the outcome GWAS dataset and harmonized. SNPs not matched in the outcome dataset were not used as proxy SNPs to ensure accuracy. During harmonization, alleles with intermediate effect allele frequencies (EAF) > 0.42 or ambiguous alleles (e.g., C/G vs. C/T) were excluded^22^.

(iv) The MR-Steiger test was employed to remove SNPs where the association with the outcome was greater than with the exposure^23^, adjusting the threshold stringently to 5×10^-5^.

### Data Source

The plasma lipidome dataset used in this analysis includes 7,174 Finnish participants, comprising 4,642 women and 2,624 men, aged between 45 and 66 years. The study conducted both univariate and multivariate genome-wide analyses through shotgun lipidomics, identifying 179 lipid species across 13 lipid categories. The research uncovered 495 genomic trait associations at 56 genetic loci, including 8 novel loci, thus unveiling genetic correlations between diseases and specific lipid species^16^. The summary-level CRC data were sourced from the FinnGen consortium's R10 release^24^, comprising summary-level data of European ancestry with 6,847 cases and 314,193 controls. Controls excluded all other cancers. A total of 19,343,950 SNPs were analyzed, and identified under the FinnGen code C3_COLORECTAL. CRC defined three endpoints: malignant neoplasm of the rectosigmoid junction, malignant neoplasm of the rectum, and malignant neoplasm of the colon. These diagnoses are defined according to the international classification of diseases (ICD)-10/9/8 classifications, as detailed at https://risteys.finregistry.fi/. Additionally, our study incorporated a trans-ancestry CRC GWAS by Rozadilla *et al.* 25, including participants of European ancestry with 78,473 cases and 107,143 controls. A total of 11,284,768 SNPs were examined. Under rigorous IV selection criteria, data from the FinnGen consortium allowed for the analysis of 161 exposure phenotypes, while the GWAS study by Rozadilla identified 151. Consequently, the FinnGen consortium's data were utilized as the discovery dataset, with the latter serving as the replication dataset for further validation.

The GWAS summary data used in this study were adjusted for quality control based on age, gender, and up to 20 principal components (PCs). Additionally, the dataset exhibited only a 2% sample overlap with the exposure, thus minimizing the risk of bias due to the winner's curse.

### Statistical analyses

For individual IVs, the primary analytical method employed is the Wald ratio, which calculates causal effects by dividing the SNP-exposure association (β_X) by the SNP-outcome association (β_Y). This approach provides an estimate of causality for each genetic variant, assuming that the IV significantly influences the exposure, operates independently of confounders, and affects the outcome solely through the exposure^26^. For multiple IVs (≥2), the primary method used is the random-effects inverse-variance weighted (IVW), where the weighting of the IVW method is based on the inverse of the variance of the Wald ratio estimates for each SNP^27^. This method is effective when all genetic variants are valid IVs. Additionally, this study incorporates MR-Egger and the weighted median method. The weighted median method is applicable when at least half of the genetic variants are invalid, whereas MR-Egger is suitable when all genetic variants might be invalid^28^. Given the stringent IV selection criteria in this research, the number of IVs ultimately available for analysis is limited. The use of online tools such as LDtrait or PhenoScanner to identify SNP traits and subjectively exclude them could result in no usable IVs or lead to "blind noise reduction," which diminishes statistical power and weakens the robustness of causal inference. To mitigate potential biases from confounding factors, heterogeneity, or horizontal pleiotropy, this study also employs advanced methods such as Bayesian weighted Mendelian randomization (BWMR)^29^, robust adjusted profile score (RAPS)^30^, constrained maximum likelihood (cML)^31^, contamination mixture (ConMix)^32^, and debiased inverse-variance weighted (dIVW)^33^, ensuring the robustness of the causal evidence.

In the MR analysis, a suite of methodological evaluations was conducted to ensure the robustness and validity of the findings. Cochran's Q test was employed to detect heterogeneity across genetic variants, with significance established at a *P* -value less than 0.05, indicating notable variability among SNPs^34^. MR-Egger regression was utilized to assess the presence of directional pleiotropy^35^, with an intercept *P*-value below 0.05 signaling significant directional pleiotropy^36^. The MR Pleiotropy Residual Sum and Outlier (MR-PRESSO) approach was applied to identify potential outliers and examine horizontal pleiotropy, with a global *P* -value under 0.05 confirming its presence^37^. Identified outliers were subsequently removed, and a leave-one-out sensitivity analysis was performed to evaluate the impact of individual SNPs on the overall results^38^. The mRnd website^39^ (https://shiny.cnsgenomics.com/mRnd/) was utilized to determine the statistical power of our analyses. To account for multiple comparisons, the Bonferroni correction was applied, setting a significance threshold for causal associations at *P* < 0.00016 [0.05/(151+161)], with values between 0.00016 and 0.05 indicating suggestive causal evidence.

### Bayesian colocalization analysis

Bayesian colocalization analysis was employed to verify whether the exposure and outcome traits shared a common causal variant within the same genomic region, thereby reducing potential bias from unaccounted confounding SNPs^40^. This method evaluates the causal relationship between two traits using five distinct hypotheses (H0 to H4). H0 assumes no genetic association between the traits; H1 suggests the variant is associated only with the exposure; H2 implies the variant is linked exclusively to the outcome; H3 reflects genetic association between the traits due to separate causal variants; and H4 indicates that both traits are influenced by a shared causal variant. A posterior probability for hypothesis 4 (PPH4) exceeding 0.8 signifies a significant shared causal relationship.

## Results

In the analyzed dataset, 161 lipidomic exposure phenotypes were identified, while the replication dataset contained 151. The IVs utilized ranged from 1 to 10 SNPs, with all IVs exhibiting F-statistics greater than 10. This significantly reduced the bias associated with weak IVs. Additionally, all IVs passed the Steiger filtering test, minimizing the potential for reverse causality. A total of 58 outliers were removed to decrease the bias from horizontal pleiotropy.

The results of all analyses are presented in **Tables S1-S2**, and specific details on all SNPs used for analysis are found in **Tables S3-S4**. This study ultimately identified 10 pieces of significant causal evidence and 13 suggestive causal evidence. Specifically, within the discovery dataset, 24 lipidomic phenotypes showed significant causal evidence after a rigorous Bonferroni correction (*P*<0.05/312) and 15 suggestive causal evidence** (Table S5)**. Further analysis of the replication dataset and meta-analysis **(Table S6)** indicated that 10 lipidomic exposure phenotypes could not be extracted and analyzed in the replication cohort, and thus were conservatively classified as suggestive evidence. After the meta-analysis, 16 lipidomic data lost statistical significance, leading to the exclusion of these phenotypes and resulting in 10 significant and 13 suggestive causal evidence. Moreover, the study had sufficient statistical power (> 85%) to detect associations for all causal evidence, further strengthening the causal inference.

**Figure 2:**
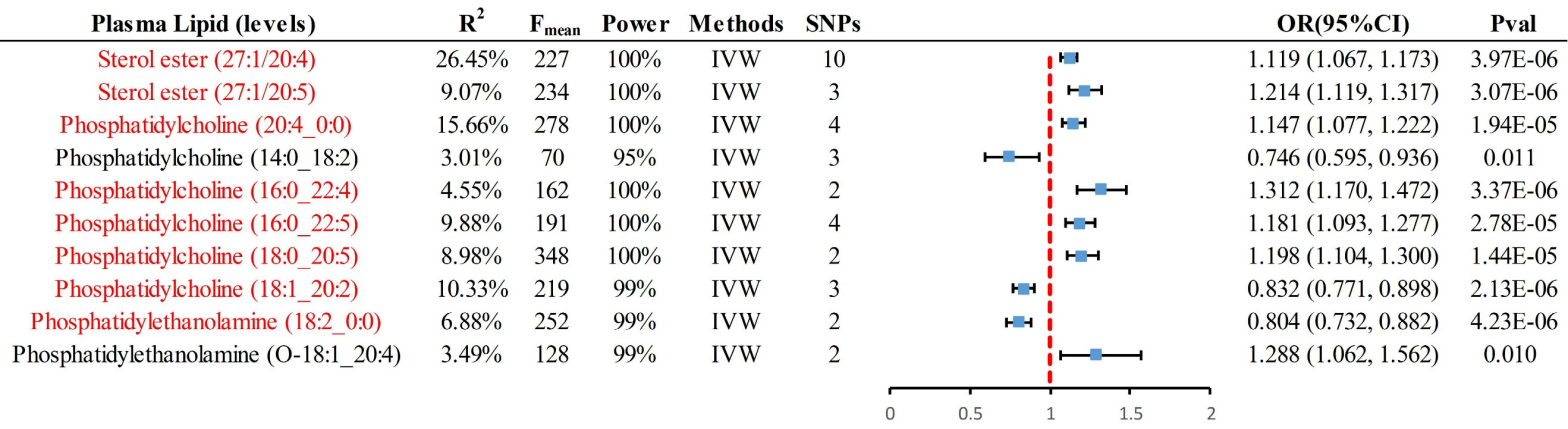
**Summary of all positive results with instrumental variables greater than or equal to 2, presented by IVW analysis.** Red markers represent significant causal associations after passing Bonferroni correction (*P* < 0.00016 (0.05/312)). IVW, inverse-variance-weighted; SNP, single nucleotide polymorphism; OR, odds ratio; CI, confidence interval.

For analyses involving multiple IVs (≥2), the primary method employed was IVW, resulting in 8 significant causal pieces of evidence and 2 suggestive pieces of evidence** (Fig. 2)**. Specifically, genetically predicted levels of sterol ester (27:1/20:4) (OR = 1.119, 95% CI 1.067-1.173, *P* = 3.97×10^-6^), sterol ester (27:1/20:5) (OR = 1.214, 95% CI 1.119-1.317, *P* = 3.07×10^-6^), phosphatidylcholine (PC) (20:4_0:0) (OR = 1.147, 95% CI 1.077-1.222, *P* = 1.94×10^-5^), PC (16:0_22:4) (OR = 1.312, 95% CI 1.170-1.472, *P* = 3.37×10^-6^), PC (16:0_22:5) (OR = 1.181, 95% CI 1.093-1.277, *P* = 2.78×10^-5^), and PC (18:0_20:5) (OR = 1.198, 95% CI 1.104-1.300, *P* = 1.44×10^-5^) were found to have a significant causal association with an increased risk of CRC. Conversely, genetically predicted levels of PC (18:1_20:2) (OR = 0.832, 95% CI 0.771-0.898, *P* = 2.13×10^-6^) and phosphatidylethanolamine (PE) (18:2_0:0) (OR = 0.804, 95% CI 0.732-0.882, *P* = 4.23×10^-6^) were significantly associated with a decreased risk of CRC. PE (O-18:1_20:4) and PC (14:0_18:2) provided suggestive evidence of an association with CRC. Supplementary methods including cML, ConMix, RAPS, dLVW, and BWMR all provided consistent causal association evidence.

**Figure 3:**
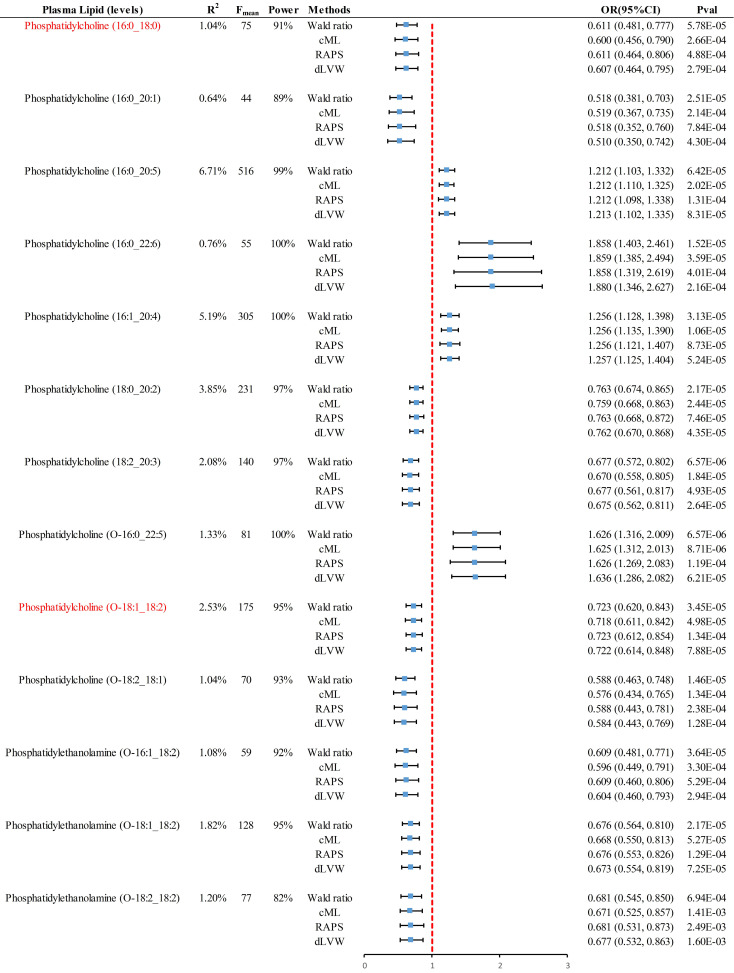
** Summary of positive results from the analysis of individual instrumental variables using four different methods.** Red markers represent significant causal associations after passing Bonferroni correction (*P* < 0.00016 (0.05/312)). RAPS, robust adjusted profile score; CML, constrained maximum likelihood; dIVW, debiased inverse-variance weighted; OR, odds ratio; CI, confidence interval.

In the case of analyses with a single IV, the Wald ratio was the primary method used, yielding 2 significant causal pieces of evidence and 11 suggestive ones** (Fig. 3)**. Specifically, genetically predicted levels of PC (16:0_18:0) (OR = 0.611, 95% CI 0.481-0.777, *P* = 5.78×10^-5^) and PE (O-18:1_18:2) (OR = 0.723, 95% CI 0.620-0.843, *P* = 3.45×10^-5^) were significantly associated with a decreased risk of CRC. Additionally, suggestive evidence of an association with CRC was found for genetically predicted levels of PC (16:0_20:1), PC (18:0_20:2), PC (18:2_20:3), PC (O-18:2_18:1), PE (O-16:1_18:2), PE (O-18:1_18:2), and PE (O-18:2_18:2). Multidimensional validation using supplementary methods such as cML, RAPS, and dLVW provided consistent evidence of causal associations. Sensitivity analyses found no evidence of horizontal pleiotropy (*P*>0.05) or heterogeneity (*P*>0.05) among these 23 pieces of causal evidence, ensuring the robustness of the results **(Table S7)**.

**Figure 4:**
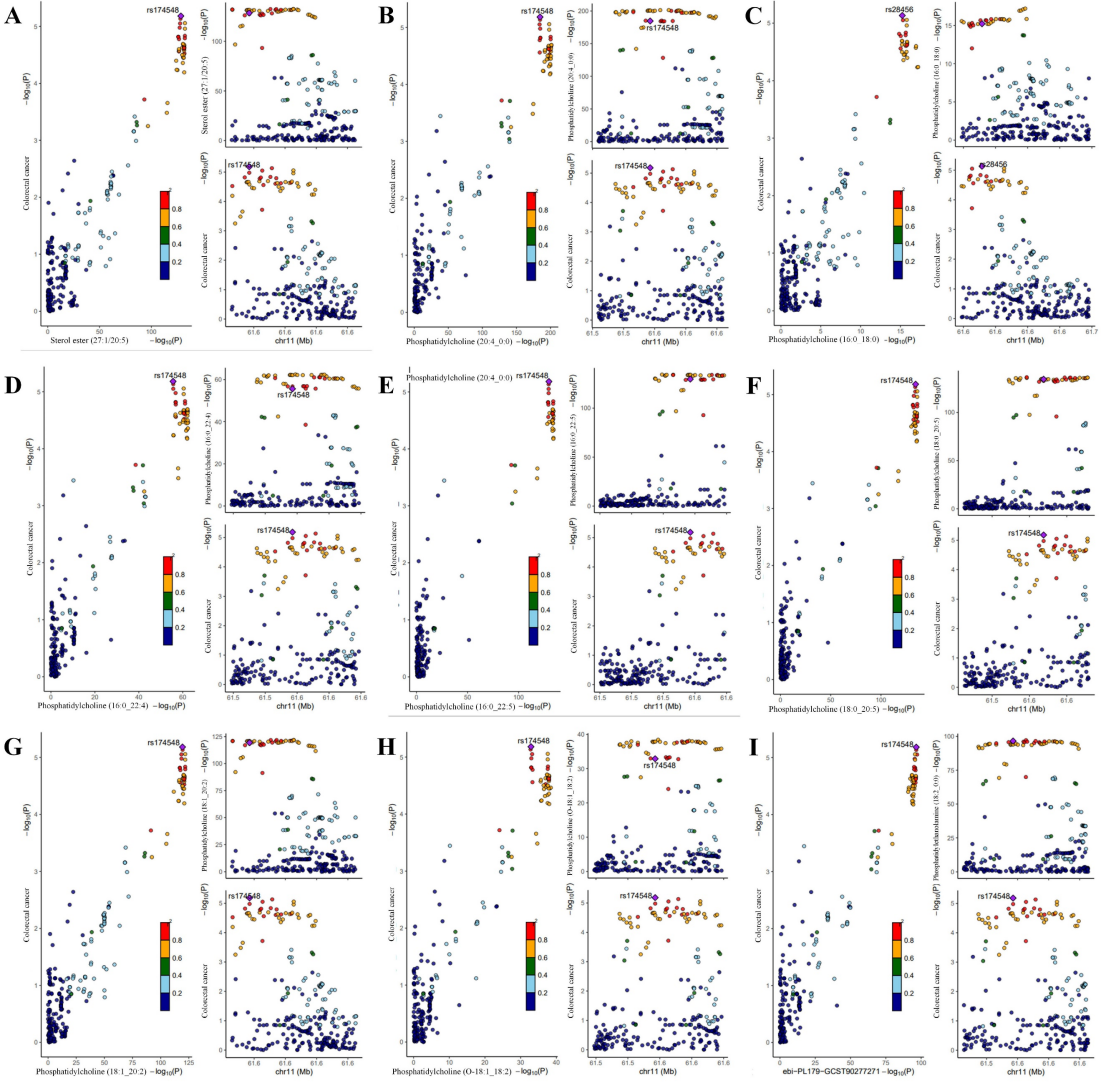
** Colocalization analysis of genetically proxied plasma lipidome and CRC (significant causal evidence).** Points were color-coded according to the LD (r^2^) of each variant relative to the variant with the highest posterior probability of colocalization within the gene region. In the left panel, -log10 P values for associations with plasma lipidome are on the x-axes, and -log10 P values for associations with the CRC on the y-axes. In the right panels, genomic positions are on the x-axes, and the y-axes show-log10 P values for plasma lipidome on the upper panel and -log10 P values with the CRC on the lower panel for the corresponding region. The genetic variants represented by the purple diamond-shaped squares in the figure are causal variants shared by the exposure and the ending. (A) Sterol ester (27:1/20:5) on CRC; (B) Phosphatidylcholine (20:4_0:0) on CRC; (C) Phosphatidylcholine (16:0_18:0) on CRC; (D) Phosphatidylcholine (16:0_22:4) on CRC; (E) Phosphatidylcholine (16:0_22:5) on CRC; (F) Phosphatidylcholine (18:0_20:5) on CRC; (G) Phosphatidylcholine (18:1_20:2) on CRC; (H) Phosphatidylcholine (O-18:1_18:2) on CRC; (I) Phosphatidylethanolamine (18:2_0:0) on CRC.

This study conducted a reverse MR analysis using CRC as the exposure and 179 lipid phenotypes as the outcome to assess potential reverse causal relationships. The results revealed no causal effect of CRC on the lipid phenotypes (Table S8), which further strengthened the robustness of the causal evidence. Additionally, among the 10 significant causal associations identified, Bayesian colocalization analysis was performed. The results showed that, with the exception of Sterol ester (27:1/20:4), the PPH4 of the remaining 9 lipid phenotypes was greater than 90%, confirming them as significant causal evidence (Table S9). Notably, rs28456 was identified as the shared causal variant for PC (16:0_18:0) and CRC, while rs174546 was the shared causal variant for the other 8 lipid phenotypes and CRC (Fig.4).

## Discussion

To our knowledge, this is the first two-sample MR analysis to investigate the association between 179 multidimensional lipid phenotypes and the risk of CRC. This study has identified 9 significant causal associations, indicating that genetically predicted levels of sterol ester (27:1/20:5), PC (20:4_0:0), PC (16:0_22:4), PC (16:0_22:5), and PC (18:0_20:5) are significantly associated with an increased risk of CRC. Conversely, genetically predicted levels of PC (18:1_20:2), PE (18:2_0:0), PC (16:0_18:0), and PC (O-18:1_18:2) are significantly associated with a decreased risk of CRC. Additionally, 14 other causal associations were identified. We will elaborate on our findings by discussing both risk-enhancing and protective lipid phenotypes.

Previous MR studies have preliminarily explored the causal associations between blood lipids and CRC risk. Shu *et al.* 41 evaluated the associations of 217 predicted metabolites, including 113 polar analytes and 104 lipid analytes, with CRC risk in different populations. Their analysis focused on lipid phenotypes such as PC, LPE, LPC, TAG, DAG, SM, and CE, some of which overlap with our study. However, our study includes a broader range of lipid phenotypes, such as PI, PEO, PE, and PCO. Furthermore, Shu *et al.* employed logistic regression models for their analysis, which may have been susceptible to confounding bias. Bull *et al.* 42 conducted an MR study examining causal relationships between 231 metabolites and CRC, similar to investigations by Bull *et al.* 43 and Yang *et al.* 44, which emphasized integrated measurements of lipoprotein subclasses and lipid content. In contrast, our research investigated a broader and more detailed set of 179 lipid phenotypes, including specific subtypes of sphingomyelins, phosphatidylcholines, and triacylglycerols. Yang *et al.* 44 assessed causal relationships between 1,400 metabolites and CRC, but their dataset focused on broader biochemical features with insufficient lipid representation. Although overlapping metabolites included ceramides, sphingomyelins, and cholesterol, their study lacked detailed subcategories to differentiate various lipid types and did not extract SNPs at genome-wide significance levels. Yuan *et al.* 45 primarily examined traditional lipid phenotypes, such as HDL-C and TG, in relation to CRC risk. Thus, previous studies offered limited depth and breadth in understanding the associations between lipids and CRC. Our study addresses these gaps by incorporating unique lipid subtypes and providing a highly detailed evaluation of causal relationships between lipid phenotypes and CRC. By employing a targeted lipidomics approach, we elucidate specific lipid-related mechanisms, avoiding the confounding effects of irrelevant metabolites commonly present in broad metabolomics datasets used in earlier studies.

In the context of CRC, persistent low-grade inflammation is considered one of the key factors promoting tumorigenesis^3^. This MR analysis identified two specific fatty acid chains in sterol esters, 20:4 (arachidonic acid) and 20:5 [Eicosapentaenoic acid (EPA), an omega-3 fatty acid], as having significant causal associations with an increased risk of CRC. On one hand, a review by Wang *et al.* systematically highlighted that arachidonic acid is metabolized via the cyclooxygenase (COX) pathway to produce prostaglandin E2 (PGE2), a known pro-inflammatory lipid mediator that plays a crucial role in promoting immune evasion in colorectal cancer^46^. Moreover, research by Neoptolemos *et al.* found that the concentration of arachidonic acid and its metabolites in human CRC tissue, compared to unaffected mucosa, is elevated, a rise potentially linked to a reduction in lipid peroxidation in rapidly growing cells or an increase in enzyme activity^47^. In terms of pharmacology, indomethacin has been demonstrated to reduce the intake of arachidonic acid and decrease the expression of fatty acid transport proteins/CD36 and peroxisome proliferator-activated receptor gamma, thereby potentially inhibiting the malignant behavior of colorectal cancer^48^. On the other hand, the specific role of EPA (20:5) within sterol esters may depend on its balance with other fatty acid types and its integration into cellular metabolism. The excessive accumulation of sterol esters containing EPA may reflect a dysregulated state of lipid metabolism, thereby affecting the tumor microenvironment and disease progression. Research by Courtney *et al.* indicates that dietary supplementation of EPA significantly reduces crypt cell proliferation while increasing apoptosis in the normal colonic mucosa of subjects with a history of colorectal adenomas^49^. Similarly, a study by Weng *et al.* suggests that the combination of EPA, epigallocatechin-3-gallate (EGCG), and proanthocyanidins significantly inhibits the mTOR signaling pathway in CRC cells, indicating that EPA may suppress cancer progression by modulating key signaling pathways^50^. Additionally, Gaszewska *et al.* noted that EPA could influence the survival and proliferation of CRC cells by regulating enzymes such as SCD1^51^. The accumulation of sterol esters might lead to alterations in intracellular signaling pathways, such as activating certain growth factor signaling pathways or inhibiting apoptotic pathways, thus promoting the proliferation and survival of tumor cells. Therefore, sterol esters containing 27:1/20:4 and 27:1/20:5 may play a role in developing CRC through multiple mechanisms, offering potential pathways for revealing new therapeutic targets and preventive strategies.

PC, a major phospholipid component of cell membranes, has been identified through this MR analysis as having significant causal associations with an increased risk of CRC for specific fatty acid chains (20:4_0:0, 16:0_22:4, 16:0_22:5, 18:0_20:5). Kurabe *et al.* utilized imaging mass spectrometry to identify PC (16:0/16:1) as a novel biomarker in CRC^52^, although this MR analysis did not establish it as significant causal evidence, it was validated in the replication cohort** (Table S2)**. Kühn *et al.* focused on metabolic changes preceding CRC diagnosis, highlighting alterations in lipid composition, including PC, years before the diagnosis of common malignancies^53^. As mentioned earlier, these PC molecules, enriched with polyunsaturated fatty acids (PUFAs) such as arachidonic acid (20:4), docosatetraenoic acid (22:4), and docosapentaenoic acid (22:5), can serve as precursors to inflammatory mediators, promoting a chronic inflammatory state^46^. Moreover, studies have shown that phospholipase C specific to PC (PC-PLC) is associated with apoptosis induced by the deprivation of survival factors in endothelial cells^54^, underscoring the vital role of PC in regulating tumor cell behavior, particularly through signaling pathways related to cell survival and apoptosis. Protective effects were also observed for PC (18:1_20:2, 16:0_18:0, O-18:1_18:2), with molecules rich in monounsaturated fatty acids (MUFAs) and PUFAs, such as 18:1_20:2 and O-18:1_18:2, potentially increasing membrane fluidity and facilitating the normal function of signaling pathways associated with cell proliferation, migration, and apoptosis. Therefore, by revealing the causal links between specific PCs and CRC risk, this study offers new insights for future preventive and therapeutic strategies against CRC.

PE constitutes an essential component of cell membranes, and this MR analysis has identified a protective causal effect of PE (18:2_0:0) against CRC. PE (18:2_0:0) contains two linoleic acid (18:2) chains, a PUFA belonging to the omega-6 family, whose metabolites play complex roles in regulating inflammation^55^. Although omega-6 family PUFAs are often associated with pro-inflammatory responses, research by Kumar *et al.* highlights that a moderate intake of linoleic acid can exert anti-inflammatory effects through specific metabolic pathways^56^. Furthermore, PE with high linoleic acid content may confer protective effects by alleviating oxidative stress and reducing the damage free radicals cause to cells. Therefore, PE (18:2_0:0) may help modulate the gut environment through its anti-inflammatory and antioxidant properties, reducing DNA damage and the accumulation of mutations, thereby lowering the risk of CRC. Future research is needed to further explore the mechanisms by which PE operates in CRC and how dietary and lifestyle adjustments can optimize the body's lipid profile, offering practical guidance for the prevention and management of CRC.

This study boasts several strengths. Firstly, the MR analysis utilized two CRC datasets, with final causal evidence determined through meta-analysis and substantiated by stringent Bonferroni correction, demonstrating sufficient statistical power to affirm the robustness of our causal associations. Secondly, nine different supplementary methods were employed for multidimensional validation, substantially minimizing potential biases from pleiotropy and heterogeneity, thereby ensuring more robust results. Furthermore, a rigorous screening process was executed, with all IVs exhibiting F-statistics greater than 10, mitigating the bias from weak IVs, and all passing MR-Steiger filtering. Importantly, this research pioneers the causal analysis between lipidomics and CRC, laying a foundation for future studies. However, the study also has limitations. Primarily, the analysis focused on populations of European ancestry, thereby reducing the generalizability across diverse ethnic groups, necessitating further validation in trans-ancestral studies. Lastly, the study relies on summary-level GWAS data, precluding subgroup analysis by age, sex, etc., and the interpretation of nonlinear causal effects, a common limitation of two-sample MR analyses.

This study utilized genetic data to investigate the causal relationship between multidimensional lipid profiles and CRC risk in European populations, laying a foundational basis for further research in this area. Future studies should employ cell or animal models to explore the functional impact of specific lipid species on the pathogenesis of colorectal cancer. Additionally, integrating multi-omics data should be considered to examine the interactions between lipid molecules and other metabolic or immune pathways. Such an approach could provide new insights into the mechanisms underlying CRC development. Furthermore, combining multiple experimental methods with MR analysis for triangulation could uncover novel therapeutic targets and offer crucial scientific evidence for the development of precise prevention and treatment strategies for colorectal cancer.

## Conclusions

This study identifies specific lipid species that significantly influence the risk of CRC. Our findings reveal novel associations that enhance our understanding of lipid-related mechanisms in CRC development. These results not only improve CRC risk assessment but also highlight potential lipid targets for therapeutic intervention. Future research should focus on elucidating the biological pathways underlying these associations and evaluating the clinical utility of lipid-based strategies in CRC prevention and treatment.

## Supplementary Material

Supplementary tables.

## Abbreviations

CRC: colorectal cancer
LDL-C: low-density lipoprotein cholesterol
HDL-C: high-density lipoprotein cholesterol
TG: triglycerides
TC: total cholesterol
MR: Mendelian randomization
IV: instrumental variable
GWAS: genome-wide association study
PC: phosphatidylcholine
PE: phosphatidylethanolamine
SNP: single nucleotide polymorphism
IVW: inverse-variance-weighted
RAPS: robust adjusted profile score
CML: constrained maximum likelihood
dIVW: debiased inverse-variance-weighted
ConMix: contamination mixture
EAF: effect allele frequency
MAF: minor allele frequency
BWMR: Bayesian weighted Mendelian randomization
OR: odds ratio
CI: confidence interval
ICD: international classification of diseases
SNP: single nucleotide polymorphism
STROBE-MR: Strengthening the Reporting of Observational Studies in Epidemiology using Mendelian Randomization
PPH4: posterior probability for hypothesis 4
