# Supplementary material for: Exploring The Causal Relationship Between Lipid Profiles and Colorectal Cancer Through Mendelian Randomization: A Multidimensional Plasma Lipid Composition Perspective
Source: J Cancer. 2025 Feb 28;16(6):1848–59. doi: 10.7150/jca.103247 (PMC11905406; doi:10.7150/jca.103247)
Supplement: Supplementary file 1 — Supplementary tables. [file jcav16p1848s1.pdf]

Table S1.Summary of all results in the discovery dataset

exposure	GWAS Catalog ID	outcome	method	nsnp	b	se	or	or_lci95	or_uci95	pval
Sterol ester (27:1/14:0) levels	GCST90277238	CRC	IVW	2	-0.24468	0.182939	0.782954	0.547035	1.120617	0.181059
Sterol ester (27:1/16:0) levels	GCST90277240	CRC	IVW	8	0.072089	0.066605	1.074752	0.94322	1.224625	0.279099
Sterol ester (27:1/16:1) levels	GCST90277241	CRC	IVW	3	0.007237	0.109858	1.007264	0.812138	1.24927	0.947475
Sterol ester (27:1/17:0) levels	GCST90277242	CRC	IVW	2	-0.06556	0.286027	0.936542	0.534634	1.640583	0.818705
Sterol ester (27:1/17:1) levels	GCST90277243	CRC	Wald ratio	1	-0.05262	0.197136	0.948744	0.64468	1.396222	0.789545
Sterol ester (27:1/18:0) levels	GCST90277244	CRC	IVW	6	0.02033	0.076018	1.020538	0.879268	1.184505	0.789136
Sterol ester (27:1/18:1) levels	GCST90277245	CRC	IVW	6	0.087143	0.078452	1.091052	0.935547	1.272405	0.266665
Sterol ester (27:1/18:2) levels	GCST90277246	CRC	IVW	8	0.070101	0.06416	1.072616	0.945868	1.216349	0.274571
Sterol ester (27:1/18:3) levels	GCST90277247	CRC	IVW	5	0.209759	0.124007	1.23338	0.967253	1.57273	0.090741
Sterol ester (27:1/20:2) levels	GCST90277248	CRC	IVW	3	-0.02215	0.120748	0.978094	0.771965	1.239264	0.854458
Sterol ester (27:1/20:3) levels	GCST90277249	CRC	IVW	6	0.118341	0.080988	1.125627	0.960408	1.319269	0.143959
Sterol ester (27:1/20:4) levels	GCST90277250	CRC	IVW	10	0.112198	0.024322	1.118734	1.066655	1.173356	3.97E-06
Sterol ester (27:1/20:5) levels	GCST90277251	CRC	IVW	3	0.19416	0.041611	1.214291	1.119187	1.317476	3.07E-06
Sterol ester (27:1/22:6) levels	GCST90277252	CRC	IVW	2	0.278724	0.163471	1.321443	0.959178	1.82053	0.088187
Ceramide (d40:1) levels	GCST90277253	CRC	IVW	6	-0.01633	0.053354	0.983804	0.886121	1.092256	0.759578
Ceramide (d40:2) levels	GCST90277254	CRC	IVW	2	0.017311	0.079259	1.017461	0.871067	1.188459	0.827113
Ceramide (d42:1) levels	GCST90277255	CRC	IVW	3	-0.00204	0.066699	0.997958	0.875662	1.137333	0.975551
Ceramide (d42:2) levels	GCST90277256	CRC	IVW	8	-0.05282	0.04675	0.94855	0.865497	1.039572	0.258537
Cholesterol levels	GCST90277257	CRC	Wald ratio	1	0.082553	0.182989	1.086057	0.758733	1.55459	0.65189
Diacylglycerol (16:0_18:1) levels	GCST90277258	CRC	Wald ratio	1	-0.07493	0.134388	0.927808	0.712959	1.207401	0.577139
Diacylglycerol (16:0_18:2) levels	GCST90277259	CRC	Wald ratio	1	-0.06201	0.111211	0.939876	0.755799	1.168786	0.577139
Diacylglycerol (16:1_18:1) levels	GCST90277260	CRC	IVW	2	-0.13741	0.112348	0.871615	0.699347	1.086318	0.221308
Diacylglycerol (18:1_18:1) levels	GCST90277261	CRC	IVW	5	-0.07103	0.065959	0.931434	0.818478	1.059979	0.281532
Diacylglycerol (18:1_18:2) levels	GCST90277262	CRC	IVW	6	-0.08354	0.061456	0.919851	0.815465	1.037599	0.174016
Diacylglycerol (18:1_18:3) levels	GCST90277263	CRC	Wald ratio	1	-0.08176	0.146636	0.921493	0.69131	1.22832	0.577139
Phosphatidylcholine (16:0_0:0) levels	GCST90277264	CRC	IVW	2	-0.08345	0.119585	0.91994	0.727724	1.162926	0.485299
Phosphatidylcholine (18:1_0:0) levels	GCST90277266	CRC	Wald ratio	1	0.014366	0.165344	1.01447	0.733661	1.402759	0.930762
Phosphatidylcholine (18:2_0:0) levels	GCST90277267	CRC	IVW	3	-0.17323	0.295286	0.840944	0.471427	1.500097	0.557436
Phosphatidylcholine (20:4_0:0) levels	GCST90277268	CRC	IVW	4	0.137572	0.032207	1.147485	1.077288	1.222256	1.94E-05
Phosphatidylethanolamine (18:0_0:0) levels	GCST90277269	CRC	IVW	2	0.026055	0.108871	1.026398	0.829168	1.270541	0.810857
Phosphatidylethanolamine (18:2_0:0) levels	GCST90277271	CRC	IVW	2	-0.21874	0.047552	0.803534	0.732027	0.882026	4.23E-06
Phosphatidylcholine (14:0_18:1) levels	GCST90277273	CRC	IVW	2	-0.12805	0.108363	0.879811	0.711458	1.088002	0.23734
Phosphatidylcholine (14:0_18:2) levels	GCST90277274	CRC	IVW	3	-0.29276	0.115681	0.746204	0.594823	0.936111	0.011383
Phosphatidylcholine (15:0_18:2) levels	GCST90277276	CRC	IVW	6	-0.15113	0.059576	0.859739	0.764988	0.966226	0.01119
Phosphatidylcholine (16:0_16:0) levels	GCST90277277	CRC	IVW	4	0.208085	0.128748	1.231318	0.956704	1.584758	0.106047
Phosphatidylcholine (16:0_16:1) levels	GCST90277278	CRC	Wald ratio	1	-0.20646	0.171863	0.81346	0.580823	1.139275	0.229634
Phosphatidylcholine (16:0_18:0) levels	GCST90277280	CRC	Wald ratio	1	-0.49197	0.122337	0.611421	0.481067	0.777098	5.78E-05
Phosphatidylcholine (16:0_18:1) levels	GCST90277281	CRC	IVW	2	-0.08434	0.135668	0.919114	0.704509	1.199091	0.534138
Phosphatidylcholine (16:0_18:2) levels	GCST90277282	CRC	IVW	7	-0.12712	0.056015	0.880625	0.789061	0.982815	0.023241
Phosphatidylcholine (16:0_18:3) levels	GCST90277283	CRC	IVW	3	-0.22337	0.212803	0.799819	0.527047	1.213762	0.293877
Phosphatidylcholine (16:0_20:1) levels	GCST90277284	CRC	Wald ratio	1	-0.65861	0.156282	0.517571	0.381013	0.703071	2.51E-05
Phosphatidylcholine (16:0_20:2) levels	GCST90277285	CRC	IVW	6	-0.19542	0.06242	0.822488	0.727775	0.929528	0.001744
Phosphatidylcholine (16:0_20:3) levels	GCST90277286	CRC	IVW	2	0.010286	0.152362	1.010339	0.749504	1.361949	0.946174
Phosphatidylcholine (16:0_20:4) levels	GCST90277287	CRC	IVW	4	0.12584	0.028774	1.134101	1.07191	1.1999	1.22E-05
Phosphatidylcholine (16:0_20:5) levels	GCST90277288	CRC	Wald ratio	1	0.192537	0.048172	1.212322	1.103095	1.332364	6.42E-05
Phosphatidylcholine (16:0_22:4) levels	GCST90277289	CRC	IVW	2	0.271775	0.058483	1.312292	1.170168	1.471677	3.37E-06
Phosphatidylcholine (16:0_22:5) levels	GCST90277290	CRC	IVW	4	0.166546	0.039739	1.181218	1.092707	1.276898	2.78E-05
Phosphatidylcholine (16:0_22:6) levels	GCST90277291	CRC	Wald ratio	1	0.619762	0.143265	1.858486	1.403491	2.460986	1.52E-05
Phosphatidylcholine (16:1_18:0) levels	GCST90277292	CRC	Wald ratio	1	-0.20699	0.172304	0.813029	0.580013	1.139656	0.229634
Phosphatidylcholine (16:1_18:1) levels	GCST90277293	CRC	IVW	5	-0.26356	0.093872	0.768313	0.639194	0.923513	0.00499
Phosphatidylcholine (16:1_18:2) levels	GCST90277294	CRC	IVW	8	-0.14629	0.044865	0.863903	0.791179	0.943312	0.001111
Phosphatidylcholine (16:1_20:4) levels	GCST90277295	CRC	Wald ratio	1	0.227918	0.054736	1.255982	1.128214	1.39822	3.13E-05
Phosphatidylcholine (17:0_18:1) levels	GCST90277296	CRC	Wald ratio	1	0.023817	0.160802	1.024103	0.74725	1.403528	0.882254
Phosphatidylcholine (17:0_18:2) levels	GCST90277297	CRC	IVW	3	-0.21718	0.099897	0.804783	0.661675	0.978843	0.029701
Phosphatidylcholine (17:0_20:4) levels	GCST90277298	CRC	IVW	6	0.121906	0.037574	1.129648	1.049446	1.21598	0.001177
Phosphatidylcholine (18:0_18:1) levels	GCST90277299	CRC	IVW	2	0.087234	0.253136	1.091152	0.664373	1.792085	0.730385
Phosphatidylcholine (18:0_18:2) levels	GCST90277300	CRC	IVW	5	-0.14802	0.095485	0.862412	0.715215	1.039904	0.121089
Phosphatidylcholine (18:0_18:3) levels	GCST90277301	CRC	Wald ratio	1	-0.30707	0.167582	0.735601	0.529656	1.021623	0.0669
Phosphatidylcholine (18:0_20:2) levels	GCST90277302	CRC	Wald ratio	1	-0.27	0.063576	0.763379	0.673943	0.864683	2.17E-05
Phosphatidylcholine (18:0_20:3) levels	GCST90277303	CRC	IVW	3	-0.00902	0.107866	0.991024	0.80217	1.224339	0.933379
Phosphatidylcholine (18:0_20:4) levels	GCST90277304	CRC	IVW	7	0.103884	0.032103	1.109472	1.041812	1.181525	0.001213
Phosphatidylcholine (18:0_20:5) levels	GCST90277305	CRC	IVW	2	0.180818	0.041682	1.198197	1.104201	1.300195	1.44E-05
Phosphatidylcholine (18:0_22:5) levels	GCST90277306	CRC	IVW	2	0.211804	0.147992	1.235905	0.924723	1.651804	0.152378
Phosphatidylcholine (18:0_22:6) levels	GCST90277307	CRC	IVW	2	0.297136	0.24239	1.345998	0.836985	2.164567	0.220253
Phosphatidylcholine (18:1_18:1) levels	GCST90277308	CRC	IVW	4	-0.19281	0.127627	0.824641	0.642136	1.059018	0.130863
Phosphatidylcholine (18:1_18:2) levels	GCST90277309	CRC	IVW	5	-0.13265	0.06438	0.875769	0.771948	0.993553	0.039354
Phosphatidylcholine (18:1_20:2) levels	GCST90277311	CRC	IVW	3	-0.1839	0.038795	0.832022	0.771103	0.897755	2.13E-06
Phosphatidylcholine (18:1_20:3) levels	GCST90277312	CRC	IVW	3	0.000323	0.115324	1.000323	0.797947	1.254025	0.997765
Phosphatidylcholine (18:1_20:4) levels	GCST90277313	CRC	IVW	7	0.124063	0.031718	1.132087	1.063852	1.2047	9.17E-05
Phosphatidylcholine (18:2_18:2) levels	GCST90277314	CRC	IVW	3	-0.21319	0.149261	0.807999	0.603055	1.082591	0.153196
Phosphatidylcholine (18:2_20:3) levels	GCST90277316	CRC	Wald ratio	1	-0.38988	0.086505	0.67714	0.571537	0.802256	6.57E-06
Phosphatidylcholine (18:2_20:4) levels	GCST90277317	CRC	IVW	4	0.200423	0.125128	1.221919	0.956162	1.561541	0.109212
Phosphatidylcholine (O-16:0_16:1) levels	GCST90277319	CRC	IVW	2	0.029732	0.134877	1.030178	0.790866	1.341904	0.825532
Phosphatidylcholine (O-16:0_18:1) levels	GCST90277320	CRC	IVW	3	0.116109	0.096574	1.123118	0.929437	1.35716	0.229255
Phosphatidylcholine (O-16:0_18:2) levels	GCST90277321	CRC	IVW	2	-0.26053	0.260453	0.770643	0.462542	1.283973	0.317169
Phosphatidylcholine (O-16:0_20:3) levels	GCST90277322	CRC	IVW	3	-0.01294	0.106251	0.987148	0.801566	1.215695	0.9031
Phosphatidylcholine (O-16:0_20:4) levels	GCST90277323	CRC	IVW	3	0.170552	0.058949	1.18596	1.056554	1.331215	0.003813
Phosphatidylcholine (O-16:0_22:5) levels	GCST90277324	CRC	Wald ratio	1	0.486179	0.107872	1.626091	1.316202	2.008942	6.57E-06
Phosphatidylcholine (O-16:1_16:0) levels	GCST90277325	CRC	Wald ratio	1	-0.13815	0.097929	0.870969	0.71886	1.055265	0.158331
Phosphatidylcholine (O-16:1_18:1) levels	GCST90277327	CRC	IVW	3	0.127911	0.099684	1.136452	0.934756	1.381669	0.199434
Phosphatidylcholine (O-16:1_18:2) levels	GCST90277328	CRC	IVW	2	0.100468	0.108926	1.105688	0.893127	1.368838	0.356345
Phosphatidylcholine (O-16:1_20:3) levels	GCST90277329	CRC	IVW	3	-0.05597	0.131825	0.945568	0.730266	1.224347	0.671146
Phosphatidylcholine (O-16:1_20:4) levels	GCST90277330	CRC	IVW	3	0.121704	0.086144	1.12942	0.953955	1.33716	0.157716
Phosphatidylcholine (O-16:2_18:0) levels	GCST90277331	CRC	IVW	2	0.046521	0.075042	1.04762	0.904329	1.213615	0.535304
Phosphatidylcholine (O-17:0_17:1) levels	GCST90277333	CRC	Wald ratio	1	0.157266	0.164036	1.170306	0.848534	1.614098	0.337697
Phosphatidylcholine (O-18:0_16:1) levels	GCST90277335	CRC	Wald ratio	1	-0.06178	0.152513	0.940089	0.697184	1.267624	0.685414
Phosphatidylcholine (O-18:0_20:4) levels	GCST90277336	CRC	IVW	2	0.229877	0.055334	1.258445	1.129102	1.402604	3.26E-05
Phosphatidylcholine (O-18:1_16:0) levels	GCST90277337	CRC	IVW	3	0.145971	0.094739	1.157163	0.961061	1.393278	0.123372
Phosphatidylcholine (O-18:1_18:2) levels	GCST90277338	CRC	Wald ratio	1	-0.32444	0.078332	0.722935	0.620043	0.842902	3.45E-05
Phosphatidylcholine (O-18:1_20:3) levels	GCST90277339	CRC	IVW	2	-0.23425	0.199425	0.791161	0.535194	1.16955	0.240136
Phosphatidylcholine (O-18:1_20:4) levels	GCST90277340	CRC	IVW	4	0.2311	0.05402	1.259985	1.133397	1.400711	1.89E-05
Phosphatidylcholine (O-18:2_16:0) levels	GCST90277341	CRC	IVW	2	-0.29628	0.318226	0.743583	0.39852	1.387423	0.351843

Phosphatidylcholine (O-18:2_18:1) levels	GCST90277342	CRC	Wald ratio	1	-0.53042	0.122387	0.588359	0.462876	0.74786	1.46E-05
Phosphatidylcholine (O-18:2_18:2) levels	GCST90277343	CRC	IVW	2	-0.27777	0.308814	0.757472	0.413522	1.387505	0.368403
Phosphatidylcholine (O-18:2_20:4) levels	GCST90277344	CRC	IVW	2	0.228287	0.304049	1.256446	0.69236	2.280109	0.452759
Phosphatidylethanolamine (16:0_18:2) levels	GCST90277345	CRC	IVW	4	-0.08043	0.082393	0.922724	0.785123	1.084441	0.329005
Phosphatidylethanolamine (16:0_20:4) levels	GCST90277346	CRC	IVW	5	0.018968	0.032885	1.01915	0.955533	1.087002	0.564065
Phosphatidylethanolamine (18:0_18:2) levels	GCST90277347	CRC	IVW	9	0.011975	0.036581	1.012047	0.942025	1.087275	0.743391
Phosphatidylethanolamine (18:0_20:4) levels	GCST90277348	CRC	IVW	8	0.05872	0.049923	1.060478	0.961627	1.16949	0.239511
Phosphatidylethanolamine (18:1_18:1) levels	GCST90277349	CRC	IVW	5	-0.08701	0.095681	0.916668	0.759918	1.105751	0.363151
Phosphatidylethanolamine (O-16:1_18:2) levels	GCST90277350	CRC	Wald ratio	1	-0.4959	0.120097	0.60902	0.481286	0.770656	3.64E-05
Phosphatidylethanolamine (O-16:1_20:4) levels	GCST90277351	CRC	IVW	2	0.207859	0.228136	1.231039	0.787189	1.925152	0.362232
Phosphatidylethanolamine (O-18:1_18:2) levels	GCST90277353	CRC	Wald ratio	1	-0.39216	0.09234	0.675598	0.563751	0.809635	2.17E-05
Phosphatidylethanolamine (O-18:1_20:4) levels	GCST90277354	CRC	IVW	2	0.253125	0.098549	1.288045	1.061803	1.562492	0.010214
Phosphatidylethanolamine (O-18:2_18:2) levels	GCST90277356	CRC	Wald ratio	1	-0.38465	0.113394	0.680691	0.545038	0.850107	0.000694
Phosphatidylethanolamine (O-18:2_20:4) levels	GCST90277357	CRC	IVW	2	0.333222	0.183879	1.395457	0.973186	2.000954	0.069958
Phosphatidylinositol (16:0_18:1) levels	GCST90277358	CRC	IVW	2	-0.28625	0.427682	0.751073	0.324812	1.736731	0.503297
Phosphatidylinositol (16:0_18:2) levels	GCST90277359	CRC	IVW	3	-0.12459	0.162584	0.882859	0.641945	1.214186	0.443491
Phosphatidylinositol (16:0_20:4) levels	GCST90277360	CRC	IVW	3	0.135114	0.248373	1.144667	0.703494	1.862509	0.586443
Phosphatidylinositol (18:0_18:1) levels	GCST90277361	CRC	IVW	5	-0.02503	0.049682	0.975279	0.884787	1.075027	0.614377
Phosphatidylinositol (18:0_18:2) levels	GCST90277362	CRC	IVW	7	-0.06712	0.054776	0.935079	0.839889	1.041057	0.220412
Phosphatidylinositol (18:0_20:3) levels	GCST90277363	CRC	IVW	5	-0.1355	0.06424	0.873283	0.769968	0.990461	0.034928
Phosphatidylinositol (18:0_20:4) levels	GCST90277364	CRC	IVW	6	-0.0221	0.043063	0.978144	0.898973	1.064287	0.607829
Phosphatidylinositol (18:1_18:1) levels	GCST90277365	CRC	IVW	3	-0.08408	0.077431	0.919355	0.789901	1.070025	0.277521
Phosphatidylinositol (18:1_18:2) levels	GCST90277366	CRC	IVW	3	-0.22394	0.169411	0.799363	0.573506	1.114166	0.18621
Phosphatidylinositol (18:1_20:4) levels	GCST90277367	CRC	IVW	2	-0.02085	0.1151	0.979364	0.781572	1.22721	0.856238
Sphingomyelin (d32:1) levels	GCST90277368	CRC	IVW	3	0.063671	0.058622	1.065742	0.950061	1.195508	0.277426
Sphingomyelin (d34:0) levels	GCST90277369	CRC	IVW	4	0.02834	0.078021	1.028745	0.882866	1.198728	0.716435
Sphingomyelin (d34:1) levels	GCST90277370	CRC	IVW	7	0.090975	0.068755	1.095241	0.957159	1.253244	0.18578
Sphingomyelin (d34:2) levels	GCST90277371	CRC	IVW	5	0.232115	0.112835	1.261265	1.011019	1.573452	0.039675
Sphingomyelin (d36:1) levels	GCST90277372	CRC	IVW	9	0.072208	0.05782	1.074879	0.959715	1.203863	0.211723
Sphingomyelin (d36:2) levels	GCST90277373	CRC	IVW	4	0.150893	0.155496	1.162872	0.857375	1.577223	0.33185
Sphingomyelin (d38:1) levels	GCST90277374	CRC	IVW	10	0.066261	0.037337	1.068505	0.993104	1.149631	0.075953
Sphingomyelin (d38:2) levels	GCST90277375	CRC	IVW	5	0.014278	0.057072	1.014381	0.907027	1.134441	0.80245
Sphingomyelin (d40:1) levels	GCST90277376	CRC	IVW	8	0.07829	0.046216	1.081436	0.987781	1.18397	0.090267
Sphingomyelin (d40:2) levels	GCST90277377	CRC	IVW	6	0.088826	0.063722	1.09289	0.964574	1.238276	0.163329
Sphingomyelin (d42:2) levels	GCST90277378	CRC	IVW	9	0.095842	0.049429	1.100585	0.99896	1.212547	0.052506
Triacylglycerol (46:2) levels	GCST90277380	CRC	Wald ratio	1	-0.19636	0.163458	0.821715	0.596463	1.132033	0.229634
Triacylglycerol (48:1) levels	GCST90277382	CRC	IVW	2	-0.15639	0.121802	0.855223	0.673596	1.085823	0.199142
Triacylglycerol (48:2) levels	GCST90277383	CRC	IVW	2	-0.13801	0.106828	0.871092	0.70653	1.073983	0.196399
Triacylglycerol (48:3) levels	GCST90277384	CRC	IVW	2	-0.12738	0.09987	0.880396	0.723882	1.070752	0.202135
Triacylglycerol (49:2) levels	GCST90277386	CRC	IVW	2	-0.14002	0.111681	0.869343	0.698436	1.082072	0.209942
Triacylglycerol (50:1) levels	GCST90277387	CRC	IVW	3	-0.13197	0.096385	0.876369	0.725508	1.0586	0.170946
Triacylglycerol (50:2) levels	GCST90277388	CRC	IVW	2	-0.11894	0.095332	0.887865	0.736544	1.070274	0.21218
Triacylglycerol (50:3) levels	GCST90277389	CRC	IVW	3	-0.09301	0.074988	0.911189	0.786643	1.055454	0.214875
Triacylglycerol (50:4) levels	GCST90277390	CRC	IVW	3	-0.0927	0.073801	0.911469	0.788716	1.053326	0.2091
Triacylglycerol (50:5) levels	GCST90277391	CRC	IVW	3	-0.09547	0.077343	0.908948	0.781095	1.057729	0.217073
Triacylglycerol (51:2) levels	GCST90277393	CRC	IVW	2	-0.10887	0.091525	0.896849	0.749569	1.073067	0.234247
Triacylglycerol (51:3) levels	GCST90277394	CRC	IVW	6	-0.07837	0.060475	0.92462	0.82127	1.040975	0.194989
Triacylglycerol (51:4) levels	GCST90277395	CRC	IVW	2	-0.09707	0.083058	0.907489	0.771153	1.067928	0.242506
Triacylglycerol (52:2) levels	GCST90277396	CRC	IVW	4	-0.06631	0.071077	0.935839	0.81414	1.075729	0.35084
Triacylglycerol (52:3) levels	GCST90277397	CRC	IVW	6	-0.08981	0.054488	0.914107	0.821515	1.017135	0.099313
Triacylglycerol (52:4) levels	GCST90277398	CRC	IVW	7	-0.09542	0.054414	0.908988	0.817033	1.011292	0.079492
Triacylglycerol (52:5) levels	GCST90277399	CRC	IVW	4	-0.05617	0.06516	0.94538	0.832034	1.074166	0.388681
Triacylglycerol (52:6) levels	GCST90277400	CRC	IVW	3	-0.09599	0.078832	0.908477	0.778414	1.060273	0.22338
Triacylglycerol (53:2) levels	GCST90277401	CRC	IVW	2	-0.10092	0.08858	0.904003	0.759922	1.075402	0.254564
Triacylglycerol (53:3) levels	GCST90277402	CRC	IVW	5	-0.02043	0.076948	0.97978	0.842614	1.139274	0.790648
Triacylglycerol (53:4) levels	GCST90277403	CRC	IVW	4	-0.04526	0.065	0.955751	0.841426	1.08561	0.486258
Triacylglycerol (54:3) levels	GCST90277404	CRC	IVW	3	0.027198	0.076847	1.027572	0.883892	1.194608	0.72339
Triacylglycerol (54:4) levels	GCST90277405	CRC	IVW	6	-0.07259	0.089337	0.929983	0.780602	1.10795	0.416486
Triacylglycerol (54:5) levels	GCST90277406	CRC	IVW	4	-0.01323	0.077998	0.986853	0.846953	1.149861	0.865265
Triacylglycerol (54:6) levels	GCST90277407	CRC	IVW	3	0.078554	0.258085	1.081721	0.652273	1.793913	0.760845
Triacylglycerol (54:7) levels	GCST90277408	CRC	IVW	3	-0.1143	0.09573	0.891987	0.739387	1.076083	0.232471
Triacylglycerol (56:3) levels	GCST90277409	CRC	IVW	2	-0.04833	0.090289	0.952823	0.798282	1.137282	0.59249
Triacylglycerol (56:4) levels	GCST90277410	CRC	IVW	3	-0.1374	0.214272	0.871619	0.572709	1.326535	0.521357
Triacylglycerol (56:5) levels	GCST90277411	CRC	IVW	3	-0.01913	0.07826	0.981054	0.841544	1.143692	0.806913
Triacylglycerol (56:6) levels	GCST90277412	CRC	IVW	6	-0.04098	0.054832	0.959849	0.862043	1.06875	0.454838
Triacylglycerol (56:7) levels	GCST90277413	CRC	IVW	4	-0.05171	0.073824	0.949606	0.82168	1.097448	0.483666
Triacylglycerol (56:8) levels	GCST90277414	CRC	IVW	4	0.021623	0.079102	1.021858	0.8751	1.193228	0.784581
Triacylglycerol (58:7) levels	GCST90277415	CRC	IVW	5	-0.02577	0.065425	0.974561	0.857272	1.107898	0.693687
Triacylglycerol (58:8) levels	GCST90277416	CRC	IVW	2	-0.07253	0.110767	0.930041	0.748542	1.15555	0.512618

These are selected and significantly reduced contents. Showing results for the main method only. Readers may request the nonmodified whole contents by contacting the corresponding authors of the current study: weiguoli1987@163.com (G.W.); Huojiege@jsatcm.com (J. H.)

Table S2.Summary of all results in the replication dataset

exposure	GWAS Catalog ID	outcome	method	nsnp	b	se	or	or_lci95	or_uci95	pval
Sterol ester (27:1/14:0) levels	GCST90277238	CRC	IVW	2	0.12224	0.06879	1.130025	0.987491	1.293133	0.075567
Sterol ester (27:1/16:0) levels	GCST90277240	CRC	IVW	8	0.061863	0.033198	1.063817	0.996799	1.13534	0.062401
Sterol ester (27:1/16:1) levels	GCST90277241	CRC	IVW	3	0.082276	0.114437	1.085756	0.867604	1.35876	0.472162
Sterol ester (27:1/17:0) levels	GCST90277242	CRC	IVW	2	0.087512	0.055193	1.091455	0.979546	1.216149	0.112838
Sterol ester (27:1/17:1) levels	GCST90277243	CRC	Wald ratio	1	0.268266	0.190929	1.307695	0.899465	1.901203	0.160005
Sterol ester (27:1/18:0) levels	GCST90277244	CRC	IVW	7	0.048252	0.032274	1.049435	0.985107	1.117964	0.134892
Sterol ester (27:1/18:1) levels	GCST90277245	CRC	IVW	6	0.110356	0.050562	1.116675	1.011317	1.233009	0.029067
Sterol ester (27:1/18:2) levels	GCST90277246	CRC	IVW	6	-0.03057	0.034994	0.969891	0.905598	1.038749	0.382321
Sterol ester (27:1/18:3) levels	GCST90277247	CRC	Wald ratio	1	0.33979	0.159344	1.404652	1.027856	1.919576	0.032972
Sterol ester (27:1/20:2) levels	GCST90277248	CRC	IVW	3	0.04343	0.024961	1.044387	0.994521	1.096754	0.08188
Sterol ester (27:1/20:3) levels	GCST90277249	CRC	IVW	5	0.144932	0.053685	1.155961	1.040509	1.284223	0.00694
Sterol ester (27:1/20:4) levels	GCST90277250	CRC	IVW	9	0.092943	0.032799	1.097399	1.029073	1.170263	0.0046
Sterol ester (27:1/20:5) levels	GCST90277251	CRC	IVW	2	0.163804	0.066508	1.177983	1.034015	1.341997	0.013781
Sterol ester (27:1/22:6) levels	GCST90277252	CRC	Wald ratio	1	0.100617	0.051633	1.105854	0.999418	1.223625	0.051329
Ceramide (d40:1) levels	GCST90277253	CRC	IVW	6	-0.04141	0.042402	0.959435	0.882921	1.042579	0.328755
Ceramide (d40:2) levels	GCST90277254	CRC	IVW	2	-0.02107	0.027802	0.979146	0.927218	1.033982	0.448437
Ceramide (d42:1) levels	GCST90277255	CRC	IVW	3	-0.02296	0.030214	0.9773	0.921106	1.036922	0.447261
Ceramide (d42:2) levels	GCST90277256	CRC	IVW	8	-0.03563	0.028646	0.964995	0.912308	1.020725	0.213536
Cholesterol levels	GCST90277257	CRC	Wald ratio	1	0.227998	0.1552	1.256082	0.926636	1.702657	0.141818
Diacylglycerol (16:0_18:1) levels	GCST90277258	CRC	Wald ratio	1	0.01006	0.057065	1.01011	0.903221	1.129649	0.860072
Diacylglycerol (16:0_18:2) levels	GCST90277259	CRC	Wald ratio	1	0.008325	0.047223	1.008359	0.919217	1.106146	0.860072
Diacylglycerol (16:1_18:1) levels	GCST90277260	CRC	IVW	2	-0.12912	0.145735	0.878869	0.660499	1.169434	0.375621
Diacylglycerol (18:1_18:1) levels	GCST90277261	CRC	IVW	4	-0.00209	0.035936	0.997915	0.930045	1.070738	0.953684
Diacylglycerol (18:1_18:2) levels	GCST90277262	CRC	IVW	4	-0.04121	0.048023	0.95963	0.873426	1.054341	0.39084
Diacylglycerol (18:1_18:3) levels	GCST90277263	CRC	Wald ratio	1	0.010976	0.062266	1.011037	0.894881	1.14227	0.860072
Phosphatidylcholine (16:0_0:0) levels	GCST90277264	CRC	IVW	2	-0.05672	0.215322	0.94486	0.619558	1.440965	0.792233
Phosphatidylcholine (18:1_0:0) levels	GCST90277266	CRC	Wald ratio	1	0.129592	0.067004	1.138364	0.998266	1.298124	0.0531
Phosphatidylcholine (18:2_0:0) levels	GCST90277267	CRC	IVW	2	0.1219	0.066054	1.129641	0.992463	1.285779	0.06497
Phosphatidylcholine (20:4_0:0) levels	GCST90277268	CRC	IVW	3	0.080504	0.053417	1.083833	0.976097	1.20346	0.131789
Phosphatidylethanolamine (18:0_0:0) levels	GCST90277269	CRC	IVW	2	0.092015	0.04483	1.096382	1.004157	1.197076	0.040117
Phosphatidylethanolamine (18:2_0:0) levels	GCST90277271	CRC	Wald ratio	1	-0.2734	0.065505	0.760792	0.669124	0.865017	3.00E-05
Phosphatidylcholine (14:0_18:1) levels	GCST90277273	CRC	Wald ratio	1	-0.29793	0.071384	0.742352	0.645427	0.853834	3.00E-05
Phosphatidylcholine (14:0_18:2) levels	GCST90277274	CRC	Wald ratio	1	-0.28518	0.066671	0.751877	0.659774	0.856838	1.89E-05
Phosphatidylcholine (15:0_18:2) levels	GCST90277276	CRC	IVW	5	0.024758	0.048892	1.025067	0.931395	1.128159	0.612597
Phosphatidylcholine (16:0_16:0) levels	GCST90277277	CRC	IVW	4	0.108648	0.038479	1.11477	1.033788	1.202096	0.004749
Phosphatidylcholine (16:0_16:1) levels	GCST90277278	CRC	Wald ratio	1	-0.28987	0.069452	0.748361	0.653119	0.857492	3.00E-05
Phosphatidylcholine (16:0_18:0) levels	GCST90277280	CRC	Wald ratio	1	-0.28562	0.052533	0.751551	0.678018	0.833059	5.42E-08
Phosphatidylcholine (16:0_18:1) levels	GCST90277281	CRC	IVW	2	-0.13013	0.187257	0.877984	0.608261	1.26731	0.48711
Phosphatidylcholine (16:0_18:2) levels	GCST90277282	CRC	IVW	6	0.016626	0.038905	1.016765	0.942116	1.097329	0.669117
Phosphatidylcholine (16:0_18:3) levels	GCST90277283	CRC	IVW	3	-0.18146	0.144745	0.834053	0.628035	1.107651	0.209971
Phosphatidylcholine (16:0_20:2) levels	GCST90277285	CRC	IVW	4	-0.0106	0.039161	0.989452	0.916348	1.068389	0.786567
Phosphatidylcholine (16:0_20:3) levels	GCST90277286	CRC	IVW	2	0.016724	0.088845	1.016865	0.854352	1.21029	0.850687
Phosphatidylcholine (16:0_20:4) levels	GCST90277287	CRC	IVW	3	-0.00836	0.05102	0.991674	0.897305	1.095968	0.869834
Phosphatidylcholine (16:0_22:4) levels	GCST90277289	CRC	IVW	2	0.074063	0.123506	1.076875	0.845346	1.371815	0.548725
Phosphatidylcholine (16:0_22:5) levels	GCST90277290	CRC	IVW	2	0.115339	0.068579	1.122254	0.981105	1.283709	0.0926
Phosphatidylcholine (16:1_18:0) levels	GCST90277292	CRC	Wald ratio	1	-0.29061	0.06963	0.747805	0.652406	0.857154	3.00E-05
Phosphatidylcholine (16:1_18:1) levels	GCST90277293	CRC	IVW	4	-0.07732	0.079965	0.925592	0.79132	1.082647	0.333569
Phosphatidylcholine (16:1_18:2) levels	GCST90277294	CRC	IVW	4	-0.03563	0.043811	0.964997	0.885592	1.051522	0.416061
Phosphatidylcholine (17:0_18:1) levels	GCST90277296	CRC	Wald ratio	1	0.047293	0.068544	1.048429	0.916628	1.199181	0.490218
Phosphatidylcholine (17:0_18:2) levels	GCST90277297	CRC	IVW	2	-0.00756	0.051506	0.992471	0.897171	1.097894	0.88334
Phosphatidylcholine (17:0_20:4) levels	GCST90277298	CRC	IVW	5	0.035355	0.042224	1.035987	0.953703	1.125371	0.402409
Phosphatidylcholine (18:0_18:1) levels	GCST90277299	CRC	IVW	2	0.01868	0.05498	1.018856	0.914771	1.134783	0.734039
Phosphatidylcholine (18:0_18:2) levels	GCST90277300	CRC	IVW	4	0.055633	0.037831	1.05721	0.981655	1.138579	0.141406
Phosphatidylcholine (18:0_18:3) levels	GCST90277301	CRC	Wald ratio	1	0.033669	0.062141	1.034242	0.915644	1.168201	0.587951
Phosphatidylcholine (18:0_20:3) levels	GCST90277303	CRC	IVW	3	0.014903	0.060911	1.015014	0.90079	1.143723	0.806715
Phosphatidylcholine (18:0_20:4) levels	GCST90277304	CRC	IVW	6	0.013499	0.031548	1.01359	0.952814	1.078243	0.668742
Phosphatidylcholine (18:0_20:5) levels	GCST90277305	CRC	Wald ratio	1	0.17523	0.083494	1.191521	1.01165	1.403373	0.035841
Phosphatidylcholine (18:0_22:5) levels	GCST90277306	CRC	Wald ratio	1	-0.30994	0.07426	0.733493	0.634139	0.848414	3.00E-05
Phosphatidylcholine (18:0_22:6) levels	GCST90277307	CRC	Wald ratio	1	0.110179	0.072032	1.116478	0.969473	1.285775	0.126116
Phosphatidylcholine (18:1_18:1) levels	GCST90277308	CRC	IVW	3	0.096344	0.040589	1.101138	1.016931	1.192317	0.017613
Phosphatidylcholine (18:1_18:2) levels	GCST90277309	CRC	IVW	4	0.03423	0.047378	1.034823	0.943056	1.135519	0.469987
Phosphatidylcholine (18:1_20:2) levels	GCST90277311	CRC	IVW	2	-0.0751	0.044285	0.92765	0.850526	1.011766	0.089913
Phosphatidylcholine (18:1_20:3) levels	GCST90277312	CRC	IVW	3	0.023091	0.086013	1.023359	0.864594	1.211279	0.788348
Phosphatidylcholine (18:1_20:4) levels	GCST90277313	CRC	IVW	6	-0.00932	0.033217	0.990722	0.928276	1.057368	0.779001
Phosphatidylcholine (18:2_18:2) levels	GCST90277314	CRC	IVW	2	-0.06336	0.056971	0.938609	0.83944	1.049493	0.266103
Phosphatidylcholine (18:2_20:4) levels	GCST90277317	CRC	IVW	3	-0.06307	0.05019	0.938878	0.850915	1.035933	0.20889
Phosphatidylcholine (O-16:0_16:1) levels	GCST90277319	CRC	IVW	2	0.040328	0.061164	1.041152	0.923529	1.173756	0.509678
Phosphatidylcholine (O-16:0_18:1) levels	GCST90277320	CRC	IVW	3	0.054151	0.040485	1.055644	0.975115	1.142823	0.181041
Phosphatidylcholine (O-16:0_18:2) levels	GCST90277321	CRC	Wald ratio	1	0.111431	0.065028	1.117876	0.984103	1.269834	0.086608
Phosphatidylcholine (O-16:0_20:3) levels	GCST90277322	CRC	IVW	3	-0.01869	0.069952	0.981483	0.855733	1.125713	0.789324
Phosphatidylcholine (O-16:0_20:4) levels	GCST90277323	CRC	IVW	2	0.020358	0.056835	1.020567	0.912982	1.140829	0.720195
Phosphatidylcholine (O-16:1_16:0) levels	GCST90277325	CRC	Wald ratio	1	0.116384	0.043983	1.123427	1.030636	1.224572	0.008143
Phosphatidylcholine (O-16:1_18:1) levels	GCST90277327	CRC	IVW	3	0.025511	0.043205	1.02584	0.942546	1.116494	0.554874
Phosphatidylcholine (O-16:1_18:2) levels	GCST90277328	CRC	IVW	2	0.081943	0.044901	1.085394	0.993956	1.185244	0.068004
Phosphatidylcholine (O-16:1_20:3) levels	GCST90277329	CRC	IVW	3	0.045802	0.085644	1.046867	0.885094	1.238207	0.592797
Phosphatidylcholine (O-16:1_20:4) levels	GCST90277330	CRC	IVW	2	0.095197	0.036743	1.099876	1.023452	1.182006	0.009572
Phosphatidylcholine (O-16:2_18:0) levels	GCST90277331	CRC	IVW	2	0.016858	0.031978	1.017001	0.955215	1.082783	0.598077
Phosphatidylcholine (O-17:0_17:1) levels	GCST90277333	CRC	Wald ratio	1	0.113163	0.065674	1.119815	0.984563	1.273646	0.084868
Phosphatidylcholine (O-18:0_16:1) levels	GCST90277335	CRC	Wald ratio	1	-0.02095	0.052664	0.979272	0.883232	1.085756	0.69084
Phosphatidylcholine (O-18:0_20:4) levels	GCST90277336	CRC	Wald ratio	1	-0.04269	0.107935	0.95821	0.775505	1.183961	0.692477
Phosphatidylcholine (O-18:1_16:0) levels	GCST90277337	CRC	IVW	3	0.042393	0.040238	1.043305	0.964185	1.128917	0.292078
Phosphatidylcholine (O-18:1_18:2) levels	GCST90277338	CRC	Wald ratio	1	-0.2056	0.064517	0.814156	0.717447	0.923901	0.001438
Phosphatidylcholine (O-18:1_20:3) levels	GCST90277339	CRC	Wald ratio	1	0.200088	0.073452	1.22151	1.057726	1.410655	0.006449
Phosphatidylcholine (O-18:1_20:4) levels	GCST90277340	CRC	IVW	3	-0.00262	0.073439	0.997386	0.863675	1.151796	0.971565
Phosphatidylcholine (O-18:2_16:0) levels	GCST90277341	CRC	Wald ratio	1	0.117567	0.071879	1.124757	0.976953	1.294922	0.101921
Phosphatidylcholine (O-18:2_18:2) levels	GCST90277343	CRC	Wald ratio	1	0.115769	0.06756	1.122736	0.983489	1.281699	0.086608
Phosphatidylcholine (O-18:2_20:4) levels	GCST90277344	CRC	Wald ratio	1	0.238113	0.078275	1.268853	1.088384	1.479247	0.00235
Phosphatidylethanolamine (16:0_18:2) levels	GCST90277345	CRC	IVW	3	-0.00798	0.020683	0.992055	0.952643	1.033098	0.699753
Phosphatidylethanolamine (16:0_20:4) levels	GCST90277346	CRC	IVW	5	-0.00608	0.014395	0.993934	0.966282	1.022377	0.672546
Phosphatidylethanolamine (18:0_18:2) levels	GCST90277347	CRC	IVW	8	-0.01165	0.017577	0.988418	0.954947	1.023063	0.507485
Phosphatidylethanolamine (18:0_20:4) levels	GCST90277348	CRC	IVW	7	0.001814	0.02563	1.001815	0.952732	1.053428	0.943585
Phosphatidylethanolamine (18:1_18:1) levels	GCST90277349	CRC	IVW	5	-0.01629	0.023338	0.983846	0.939857	1.029895	0.485297

Phosphatidylethanolamine (O-16:1_20:4) levels	GCST90277351	CRC	IVW	2	0.136507	0.084814	1.146263	0.970708	1.353567	0.107512
Phosphatidylethanolamine (O-18:1_20:4) levels	GCST90277354	CRC	Wald ratio	1	0.148119	0.080355	1.159651	0.990668	1.357459	0.065282
Phosphatidylethanolamine (O-18:2_18:2) levels	GCST90277356	CRC	Wald ratio	1	-0.31659	0.064454	0.728627	0.642156	0.826742	9.02E-07
Phosphatidylethanolamine (O-18:2_20:4) levels	GCST90277357	CRC	Wald ratio	1	0.146502	0.080494	1.157777	0.988797	1.355636	0.068754
Phosphatidylinositol (16:0_18:1) levels	GCST90277358	CRC	Wald ratio	1	-0.11125	0.064056	0.894716	0.78915	1.014403	0.082432
Phosphatidylinositol (16:0_18:2) levels	GCST90277359	CRC	IVW	2	-0.02786	0.057208	0.972528	0.869373	1.087924	0.626309
Phosphatidylinositol (16:0_20:4) levels	GCST90277360	CRC	IVW	2	-0.20853	0.079526	0.81178	0.694615	0.948708	0.008739
Phosphatidylinositol (18:0_18:1) levels	GCST90277361	CRC	IVW	5	0.003573	0.027248	1.00358	0.951388	1.058635	0.895667
Phosphatidylinositol (18:0_18:2) levels	GCST90277362	CRC	IVW	6	0.00019	0.024634	1.00019	0.953046	1.049667	0.99384
Phosphatidylinositol (18:0_20:3) levels	GCST90277363	CRC	IVW	4	0.068187	0.026887	1.070566	1.015608	1.128497	0.011212
Phosphatidylinositol (18:0_20:4) levels	GCST90277364	CRC	IVW	5	0.041773	0.018593	1.042658	1.005344	1.081357	0.024661
Phosphatidylinositol (18:1_18:1) levels	GCST90277365	CRC	IVW	4	0.009141	0.033558	1.009183	0.94494	1.077793	0.785317
Phosphatidylinositol (18:1_18:2) levels	GCST90277366	CRC	IVW	2	-0.00277	0.092379	0.997229	0.832071	1.19517	0.976041
Phosphatidylinositol (18:1_20:4) levels	GCST90277367	CRC	IVW	2	0.016444	0.056593	1.01658	0.909848	1.135834	0.77138
Sphingomyelin (d32:1) levels	GCST90277368	CRC	IVW	3	-0.01796	0.019818	0.9822	0.944779	1.021103	0.364807
Sphingomyelin (d34:0) levels	GCST90277369	CRC	IVW	4	0.014667	0.034954	1.014775	0.947581	1.086733	0.674781
Sphingomyelin (d34:1) levels	GCST90277370	CRC	IVW	7	0.039035	0.030264	1.039806	0.979922	1.10335	0.197113
Sphingomyelin (d34:2) levels	GCST90277371	CRC	IVW	4	0.041833	0.049347	1.042721	0.946592	1.148612	0.396589
Sphingomyelin (d36:1) levels	GCST90277372	CRC	IVW	8	0.029633	0.031954	1.030077	0.967543	1.096652	0.353724
Sphingomyelin (d36:2) levels	GCST90277373	CRC	IVW	3	-0.05059	0.080852	0.950671	0.811349	1.113917	0.531527
Sphingomyelin (d38:1) levels	GCST90277374	CRC	IVW	10	-0.00602	0.017358	0.994002	0.960754	1.028402	0.728914
Sphingomyelin (d38:2) levels	GCST90277375	CRC	IVW	4	-0.03871	0.083423	0.962031	0.816917	1.132923	0.642647
Sphingomyelin (d40:1) levels	GCST90277376	CRC	IVW	8	0.013003	0.029833	1.013088	0.955548	1.074092	0.662941
Sphingomyelin (d40:2) levels	GCST90277377	CRC	IVW	6	-0.01584	0.025089	0.984288	0.937057	1.033899	0.527879
Sphingomyelin (d42:2) levels	GCST90277378	CRC	IVW	9	0.074983	0.028454	1.077866	1.019399	1.139687	0.008408
Triacylglycerol (46:2) levels	GCST90277380	CRC	Wald ratio	1	-0.27569	0.066056	0.759046	0.666869	0.863964	3.00E-05
Triacylglycerol (48:1) levels	GCST90277382	CRC	IVW	2	-0.16026	0.135059	0.85192	0.653784	1.110104	0.235382
Triacylglycerol (48:2) levels	GCST90277383	CRC	IVW	2	-0.14333	0.114728	0.866467	0.691979	1.084953	0.211551
Triacylglycerol (48:3) levels	GCST90277384	CRC	IVW	2	-0.12878	0.114092	0.879166	0.702997	1.099481	0.259002
Triacylglycerol (49:2) levels	GCST90277386	CRC	IVW	2	-0.13712	0.135708	0.871862	0.668238	1.137534	0.312285
Triacylglycerol (50:1) levels	GCST90277387	CRC	IVW	3	-0.03703	0.140216	0.963645	0.732087	1.268444	0.791695
Triacylglycerol (50:2) levels	GCST90277388	CRC	IVW	2	-0.11549	0.117554	0.890927	0.707583	1.121776	0.32587
Triacylglycerol (50:3) levels	GCST90277389	CRC	IVW	3	-0.08664	0.085021	0.917005	0.776248	1.083286	0.30817
Triacylglycerol (50:4) levels	GCST90277390	CRC	IVW	3	-0.09014	0.081477	0.913808	0.778934	1.072035	0.26861
Triacylglycerol (50:5) levels	GCST90277391	CRC	IVW	3	-0.0956	0.088739	0.908828	0.76374	1.08148	0.281345
Triacylglycerol (51:2) levels	GCST90277393	CRC	IVW	2	-0.09794	0.125385	0.906706	0.709147	1.159302	0.434751
Triacylglycerol (51:3) levels	GCST90277394	CRC	IVW	5	0.007971	0.039471	1.008003	0.93296	1.089081	0.839966
Triacylglycerol (51:4) levels	GCST90277395	CRC	IVW	2	-0.08501	0.117004	0.918506	0.730274	1.155255	0.467512
Triacylglycerol (52:2) levels	GCST90277396	CRC	IVW	2	-0.01573	0.117926	0.984398	0.78125	1.24037	0.893919
Triacylglycerol (52:3) levels	GCST90277397	CRC	IVW	5	-0.00563	0.025235	0.994386	0.946398	1.044806	0.823451
Triacylglycerol (52:4) levels	GCST90277398	CRC	IVW	6	0.004425	0.027952	1.004435	0.950887	1.060999	0.874209
Triacylglycerol (52:5) levels	GCST90277399	CRC	IVW	4	-0.05039	0.068139	0.950858	0.831984	1.086717	0.459585
Triacylglycerol (52:6) levels	GCST90277400	CRC	IVW	3	-0.07986	0.110473	0.923243	0.743498	1.146443	0.469733
Triacylglycerol (53:2) levels	GCST90277401	CRC	IVW	2	-0.08501	0.129096	0.918502	0.713167	1.182957	0.510208
Triacylglycerol (53:3) levels	GCST90277402	CRC	IVW	4	0.009929	0.039292	1.009978	0.935118	1.090832	0.800501
Triacylglycerol (53:4) levels	GCST90277403	CRC	IVW	4	-0.05999	0.065991	0.941774	0.827512	1.071813	0.363314
Triacylglycerol (54:3) levels	GCST90277404	CRC	IVW	3	0.033632	0.029454	1.034204	0.97619	1.095666	0.253518
Triacylglycerol (54:4) levels	GCST90277405	CRC	IVW	4	0.03116	0.026611	1.031651	0.979222	1.086886	0.241611
Triacylglycerol (54:5) levels	GCST90277406	CRC	IVW	3	0.028612	0.031443	1.029026	0.967523	1.094438	0.362838
Triacylglycerol (54:6) levels	GCST90277407	CRC	IVW	2	-0.09077	0.137036	0.913229	0.698123	1.194612	0.507732
Triacylglycerol (54:7) levels	GCST90277408	CRC	IVW	3	-0.09006	0.13695	0.913879	0.698738	1.195262	0.510801
Triacylglycerol (56:3) levels	GCST90277409	CRC	IVW	3	-0.01125	0.048566	0.988817	0.899034	1.087567	0.816882
Triacylglycerol (56:4) levels	GCST90277410	CRC	IVW	2	0.0264	0.036702	1.026751	0.955486	1.103332	0.47195
Triacylglycerol (56:5) levels	GCST90277411	CRC	IVW	2	0.050976	0.042613	1.052297	0.967978	1.143962	0.231602
Triacylglycerol (56:6) levels	GCST90277412	CRC	IVW	5	0.011788	0.032049	1.011858	0.950253	1.077456	0.71301
Triacylglycerol (56:7) levels	GCST90277413	CRC	IVW	2	0.03651	0.093958	1.037185	0.862735	1.24691	0.697588
Triacylglycerol (56:8) levels	GCST90277414	CRC	IVW	4	0.085638	0.065483	1.089412	0.958191	1.238603	0.190943
Triacylglycerol (58:7) levels	GCST90277415	CRC	IVW	4	0.005465	0.040449	1.00548	0.928843	1.08844	0.892523
Triacylglycerol (58:8) levels	GCST90277416	CRC	IVW	2	0.049604	0.115426	1.050855	0.83809	1.317635	0.667377

These are selected and significantly reduced contents. Showing results for the main method only. Readers may request the nonmodified whole contents by contacting the corresponding authors of the current study: weiguoli1987@163.com (G.W.); Huojiege@jsatcm.com (J. H.)

Table S3.Summary of all SNP-specific information in the discovery dataset(Red color represents excluded outliers)

					**Harmonization**		**Exposure**				**Outcome**				**Steiger-test**			
Exposure	Outcome	SNP	chr	pos	EFFECT ALLELE	OTHER ALLELE	beta.exposı	eaf.exposu	se.exposure	pval.exposure	beta.outcor	eaf.outcom	se.outcome	pval.outcome	steiger_dir	steiger_pval	R2	F
Sterol ester (27:1/20:4) levels	CRC	rs113248417	11	62059462	G	A	-0.267977	0.03689	0.044942	2.59E-09	-0.036861	0.039346	0.046075	0.423695	TRUE	7.76E-09	0.005103	36.78508
Sterol ester (27:1/20:4) levels	CRC	rs1135999	16	15038105	G	A	-0.101484	0.315252	0.017797	1.23E-08	-0.028741	0.315027	0.019039	0.131159	TRUE	6.24E-08	0.004446	32.0324
Sterol ester (27:1/20:4) levels	CRC	rs11591147	1	55039974	T	G	-0.297311	0.033215	0.046189	1.30E-10	0.060649	0.036156	0.048295	0.209192	TRUE	6.66E-10	0.005677	40.94812
Sterol ester (27:1/20:4) levels	CRC	rs174528	11	61776027	C	T	-0.64833	0.41439	0.015196	1.00E-200	-0.075685	0.420675	0.017897	2.35E-05	TRUE	0	0.204005	1838.102
Sterol ester (27:1/20:4) levels	CRC	rs182695896	4	73947510	C	A	0.295609	0.025305	0.052463	1.82E-08	0.072226	0.02586	0.056331	0.199783	TRUE	7.50E-08	0.004311	31.04966
Sterol ester (27:1/20:4) levels	CRC	rs187429064	19	19269704	G	A	-0.254328	0.053531	0.037458	1.21E-11	0.011076	0.05117	0.041499	0.789545	TRUE	2.59E-11	0.006554	47.31832
Sterol ester (27:1/20:4) levels	CRC	rs2229738	11	68794860	T	C	-0.15287	0.155986	0.023409	7.01E-11	-0.037929	0.155795	0.024965	0.128696	TRUE	4.75E-10	0.006153	44.40488
Sterol ester (27:1/20:4) levels	CRC	rs3741252	11	61744026	T	C	0.212004	0.133653	0.024519	6.46E-18	0.025172	0.129675	0.026893	0.349263	TRUE	4.54E-17	0.010409	75.43502
Sterol ester (27:1/20:4) levels	CRC	rs4382917	11	62454004	A	G	0.146345	0.255777	0.019705	1.24E-13	0.02651	0.243412	0.020939	0.205476	TRUE	8.79E-13	0.008154	58.95847
Sterol ester (27:1/20:4) levels	CRC	rs7412	19	44908822	T	C	-0.309996	0.053066	0.036791	4.29E-17	-0.004271	0.052525	0.03859	0.911869	TRUE	1.02E-16	0.009658	69.94122
Sterol ester (27:1/20:5) levels	CRC	rs174581	11	61839211	A	G	-0.4109	0.409733	0.01638	3.56E-133	-0.079816	0.415508	0.017954	8.76E-06	TRUE	3.24E-125	0.081668	637.8114
Sterol ester (27:1/20:5) levels	CRC	rs508049	11	68908029	T	C	-0.15839	0.097967	0.028652	3.35E-08	-0.024509	0.093124	0.03102	0.429468	TRUE	8.98E-08	0.004434	31.94172
Sterol ester (27:1/20:5) levels	CRC	rs7936002	11	62451557	G	A	0.109709	0.257197	0.019671	2.53E-08	0.025198	0.245379	0.020852	0.226901	TRUE	9.67E-08	0.004599	33.13571
Phosphatidylcholine (20:4_0:0) levels	CRC	rs174545	11	61801834	G	C	-0.533953	0.407641	0.017005	1.00E-200	-0.076117	0.414007	0.017945	2.22E-05	TRUE	1.99E-194	0.137689	995.2497
Phosphatidylcholine (20:4_0:0) levels	CRC	rs58451960	11	62059600	T	C	-0.169585	0.137195	0.026302	1.22E-10	-0.035106	0.139923	0.025337	0.165872	TRUE	6.12E-10	0.006809	42.72873
Phosphatidylcholine (20:4_0:0) levels	CRC	rs600626	11	75744264	G	A	-0.120776	0.232311	0.021178	1.23E-08	0.018388	0.230625	0.020981	0.380803	TRUE	3.35E-08	0.005203	32.59935
Phosphatidylcholine (20:4_0:0) levels	CRC	rs7936002	11	62451557	G	A	0.134077	0.257197	0.020945	1.65E-10	0.025198	0.245379	0.020852	0.226901	TRUE	7.00E-10	0.006869	43.10906
Phosphatidylethanolamine (18:2_0:0) levels	CRC	rs1260326	2	27508073	C	T	-0.114725	0.650878	0.017763	1.12E-10	0.02234	0.650148	0.018596	0.229635	TRUE	5.32E-10	0.005982	41.53999
Phosphatidylethanolamine (18:2_0:0) levels	CRC	rs174560	11	61814292	C	T	0.364648	0.382915	0.01709	7.02E-98	-0.080586	0.388646	0.018138	8.87E-06	TRUE	4.41E-91	0.062838	462.8587
Phosphatidylcholine (14:0_18:2) levels	CRC	rs174548	11	61803876	G	C	0.184079	0.383631	0.017505	1.15E-25	-0.081685	0.389804	0.018124	6.58E-06	TRUE	2.37E-22	0.016025	111.7857
Phosphatidylcholine (14:0_18:2) levels	CRC	rs4665972	2	27375230	C	T	-0.114678	0.620291	0.017513	6.24E-11	0.028131	0.624423	0.018277	0.123772	TRUE	4.12E-10	0.006195	42.78708
Phosphatidylcholine (14:0_18:2) levels	CRC	rs571968630	18	20932590	C	G	0.590789	0.011389	0.080052	1.76E-13	-0.042263	0.014027	0.075052	0.573352	TRUE	5.56E-13	0.007859	54.37397
Phosphatidylcholine (15:0_18:2) levels	CRC	rs10468017	15	58386313	T	C	0.111867	0.337526	0.018143	7.41E-10	0.007439	0.331296	0.018788	0.692161	TRUE	1.53E-09	0.005596	37.35246
Phosphatidylcholine (15:0_18:2) levels	CRC	rs1168036	1	62497063	G	A	0.106914	0.737927	0.019562	4.78E-08	-0.012258	0.736587	0.020021	0.540375	TRUE	1.05E-07	0.004421	29.47345
Phosphatidylcholine (15:0_18:2) levels	CRC	rs17231506	16	56960616	T	C	0.142778	0.27829	0.019212	1.20E-13	0.02011	0.278594	0.019666	0.306512	TRUE	6.03E-13	0.008189	54.79686
Phosphatidylcholine (15:0_18:2) levels	CRC	rs174566	11	61824890	G	A	0.316974	0.408433	0.017255	1.46E-73	-0.075641	0.413654	0.017944	2.49E-05	TRUE	4.76E-68	0.048551	338.6792
Phosphatidylcholine (15:0_18:2) levels	CRC	rs55727637	16	15051395	T	C	0.110967	0.298983	0.018977	5.22E-09	-0.011677	0.297998	0.01933	0.545775	TRUE	1.22E-08	0.005162	34.43595
Phosphatidylcholine (15:0_18:2) levels	CRC	rs964184	11	1.17E+08	C	G	-0.140695	0.848991	0.024077	5.34E-09	0.01394	0.855576	0.025002	0.577138	TRUE	1.20E-08	0.005076	33.85907
Phosphatidylcholine (16:0_18:0) levels	CRC	rs174601	11	61855668	T	C	0.146355	0.419025	0.01691	6.00E-18	-0.072002	0.426726	0.017905	5.78E-05	TRUE	1.85E-15	0.010429	75.37426
Phosphatidylcholine (16:0_18:2) levels	CRC	rs1532085	15	58391167	G	A	-0.110553	0.573441	0.016767	4.61E-11	-0.004959	0.577767	0.017873	0.781444	TRUE	9.61E-11	0.005979	43.14035
Phosphatidylcholine (16:0_18:2) levels	CRC	rs17231506	16	56960616	T	C	0.143843	0.27829	0.018493	8.35E-15	0.02011	0.278594	0.019666	0.306512	TRUE	5.06E-14	0.008311	60.10806
Phosphatidylcholine (16:0_18:2) levels	CRC	rs174574	11	61832870	C	A	-0.330699	0.590933	0.016603	5.66E-86	0.076186	0.585489	0.017942	2.17E-05	TRUE	1.29E-79	0.052872	400.3688
Phosphatidylcholine (16:0_18:2) levels	CRC	rs1800588	15	58431476	T	C	0.137688	0.258046	0.019308	1.09E-12	0.014862	0.24873	0.020555	0.46967	TRUE	4.00E-12	0.007259	52.4446
Phosphatidylcholine (16:0_18:2) levels	CRC	rs62039480	16	15043593	A	G	0.115988	0.332015	0.017732	6.52E-11	-0.013739	0.328903	0.018801	0.464906	TRUE	2.09E-10	0.005967	43.05466
Phosphatidylcholine (16:0_18:2) levels	CRC	rs9436221	1	62437688	C	T	-0.126689	0.261404	0.018823	1.82E-11	0.010428	0.262815	0.020051	0.603004	TRUE	4.96E-11	0.006198	44.72674
Phosphatidylcholine (16:0_18:2) levels	CRC	rs964184	11	1.17E+08	C	G	-0.158038	0.848991	0.023159	9.56E-12	0.01394	0.855576	0.025002	0.577138	TRUE	2.76E-11	0.006404	46.22633
Phosphatidylcholine (16:0_20:1) levels	CRC	rs174594	11	61852357	A	C	-0.11459	0.5815	0.017281	3.58E-11	0.07547	0.574741	0.017908	2.51E-05	TRUE	2.80E-09	0.006391	44.25288
Phosphatidylcholine (16:0_20:2) levels	CRC	rs10160784	11	75745010	C	T	-0.10964	0.232378	0.01988	3.61E-08	0.018958	0.230715	0.020978	0.366146	TRUE	1.05E-07	0.004289	30.72198
Phosphatidylcholine (16:0_20:2) levels	CRC	rs1260326	2	27508073	C	T	-0.097152	0.650878	0.017513	3.00E-08	0.02234	0.650148	0.018596	0.229635	TRUE	1.12E-07	0.00429	30.7292
Phosphatidylcholine (16:0_20:2) levels	CRC	rs1532085	15	58391167	G	A	-0.093833	0.573441	0.016856	2.69E-08	-0.004959	0.577767	0.017873	0.781444	TRUE	4.75E-08	0.004307	30.8569
Phosphatidylcholine (16:0_20:2) levels	CRC	rs174592	11	61851136	G	A	0.261677	0.418474	0.016766	4.96E-54	-0.075196	0.425184	0.017907	2.68E-05	TRUE	4.12E-49	0.033327	245.9186
Phosphatidylcholine (16:0_20:2) levels	CRC	rs1800588	15	58431476	T	C	0.10685	0.258046	0.019416	3.85E-08	0.014862	0.24873	0.020555	0.46967	TRUE	9.70E-08	0.004372	31.32046
Phosphatidylcholine (16:0_20:2) levels	CRC	rs62081852	18	26802732	T	C	0.389035	0.017663	0.067672	9.35E-09	-0.033702	0.019994	0.063535	0.595808	TRUE	2.11E-08	0.005252	37.66118
Phosphatidylcholine (16:0_20:4) levels	CRC	rs174528	11	61776027	C	T	-0.58723	0.41439	0.015544	1.00E-200	-0.075685	0.420675	0.017897	2.35E-05	TRUE	1.62E-277	0.167365	1441.617
Phosphatidylcholine (16:0_20:4) levels	CRC	rs187429064	19	19269704	G	A	-0.226182	0.053531	0.037687	2.04E-09	0.011076	0.05117	0.041499	0.789545	TRUE	3.84E-09	0.005184	37.37304
Phosphatidylcholine (16:0_20:4) levels	CRC	rs2943813	11	61490996	C	T	-0.139019	0.782953	0.020392	9.96E-12	-0.025418	0.792124	0.021902	0.245834	TRUE	5.27E-11	0.006569	47.42094
Phosphatidylcholine (16:0_20:4) levels	CRC	rs3741252	11	61744026	T	C	0.210427	0.133653	0.024533	1.17E-17	0.025172	0.129675	0.026893	0.349263	TRUE	8.10E-17	0.010254	74.30536
Phosphatidylcholine (16:0_20:5) levels	CRC	rs102274	11	61790354	C	T	-0.372594	0.408673	0.016501	4.34E-109	-0.071738	0.415679	0.017949	6.42E-05	TRUE	2.23E-102	0.067097	515.7613
Phosphatidylcholine (16:0_22:4) levels	CRC	rs174551	11	61806212	C	T	-0.289752	0.406164	0.017095	3.67E-63	-0.07436	0.412521	0.017954	3.45E-05	TRUE	5.21E-58	0.0405	290.0607
Phosphatidylcholine (16:0_22:4) levels	CRC	rs61008604	2	1.02E+08	T	G	-0.116042	0.248766	0.019847	5.24E-09	-0.045881	0.248578	0.020544	0.0255252	TRUE	4.85E-08	0.005033	34.76171
Phosphatidylcholine (16:0_22:5) levels	CRC	rs174533	11	61781553	A	G	-0.41301	0.408601	0.016346	5.08E-135	-0.073076	0.415098	0.01794	4.63E-05	TRUE	1.39E-127	0.082439	644.1918
Phosphatidylcholine (16:0_22:5) levels	CRC	rs2236514	9	1.37E+08	G	C	-0.123138	0.647094	0.017725	4.04E-12	-0.011391	0.654132	0.0187	0.54244	TRUE	1.26E-11	0.006925	50.0009
Phosphatidylcholine (16:0_22:5) levels	CRC	rs508049	11	68908029	T	C	-0.16692	0.097967	0.028607	5.62E-09	-0.024509	0.093124	0.03102	0.429468	TRUE	1.61E-08	0.004924	35.48236
Phosphatidylcholine (16:0_22:5) levels	CRC	rs62229686	21	43971391	T	C	-0.246348	0.039112	0.041965	4.54E-09	-0.027281	0.041785	0.044311	0.538105	TRUE	1.13E-08	0.004561	32.85575
Phosphatidylcholine (16:0_22:6) levels	CRC	rs174544	11	61800281	A	C	-0.126716	0.379757	0.017255	2.30E-13	-0.078534	0.386483	0.018154	1.52E-05	TRUE	3.74E-11	0.007564	54.63313
Phosphatidylcholine (16:1_18:1) levels	CRC	rs1260326	2	27508073	C	T	-0.110177	0.650878	0.017461	2.95E-10	0.02234	0.650148	0.018596	0.229635	TRUE	1.39E-09	0.005517	39.74174
Phosphatidylcholine (16:1_18:1) levels	CRC	rs146934967	18	15033501	T	C	0.41533	0.013079	0.073728	1.83E-08	-0.115575	0.014526	0.07391	0.117878	TRUE	9.51E-08	0.004453	32.04577
Phosphatidylcholine (16:1_18:1) levels	CRC	rs1532085	15	58391167	G	A	-0.092555	0.573441	0.016809	3.79E-08	-0.004959	0.577767	0.017873	0.781444	TRUE	6.64E-08	0.004191	30.14922
Phosphatidylcholine (16:1_18:1) levels	CRC	rs174560	11	61814292	C	T	0.175659	0.382915	0.017182	2.28E-24	-0.080586	0.388646	0.018138	8.87E-06	TRUE	4.11E-21	0.014582	106.0116
Phosphatidylcholine (16:1_18:1) levels	CRC	rs603424	10	1E+08	A	G	-0.176374	0.121331	0.025975	1.21E-11	0.011204	0.116436	0.027658	0.685416	TRUE	2.97E-11	0.006633	47.83458
Phosphatidylcholine (16:1_18:2) levels	CRC	rs1260326	2	27508073	C	T	-0.09847	0.650878	0.017428	1.66E-08	0.02234	0.650148	0.018596	0.229635	TRUE	6.44E-08	0.004407	31.70467
Phosphatidylcholine (16:1_18:2) levels	CRC	rs1532085	15	58391167	G	A	-0.097216	0.573441	0.016768	7.02E-09	-0.004959	0.577767	0.017873	0.781444	TRUE	1.28E-08	0.004624	33.27236
Phosphatidylcholine (16:1_18:2) levels	CRC	rs174574	11	61832870	C	A	-0.365719	0.590933	0.016496	2.11E-105	0.076186	0.585489	0.017942	2.17E-05	TRUE	2.07E-98	0.064663	495.2046
Phosphatidylcholine (16:1_18:2) levels	CRC	rs1800588	15	58431476	T	C	0.115653	0.258046	0.019312	2.21E-09	0.014862	0.24873	0.020555	0.46967	TRUE	6.21E-09	0.005122	36.87593
Phosphatidylcholine (16:1_18:2) levels	CRC	rs56156922	16	56953457	C	T	0.113979	0.278673	0.018542	8.30E-10	0.018911	0.278938	0.019696	0.336992	TRUE	2.99E-09	0.005223	37.60763
Phosphatidylcholine (16:1_18:2) levels	CRC	rs571968630	18	20932590	C	G	0.493926	0.011389	0.079177	4.67E-10	-0.042263	0.014027	0.075052	0.573352	TRUE	1.20E-09	0.005493	39.56701

Phosphatidylcholine (16:1_18:2) levels	CRC	rs603424	10	1E+08	A	G	-0.161319	0.121331	0.025924	5.15E-10	0.011204	0.116436	0.027658	0.685416	TRUE	1.14E-09	0.005549	39.96774
Phosphatidylcholine (16:1_18:2) levels	CRC	rs7936002	11	62451557	G	A	-0.115471	0.257197	0.01963	4.22E-09	0.025198	0.245379	0.020852	0.226901	TRUE	1.76E-08	0.005095	36.67994
Phosphatidylcholine (16:1_20:4) levels	CRC	rs174535	11	61783884	C	T	-0.327652	0.409133	0.019152	5.33E-64	-0.074678	0.415943	0.017934	3.13E-05	TRUE	1.57E-59	0.051905	304.5012
Phosphatidylcholine (17:0_18:2) levels	CRC	rs174566	11	61824890	G	A	0.258705	0.408433	0.016784	9.03E-53	-0.075641	0.413654	0.017944	2.49E-05	TRUE	6.95E-48	0.032342	239.5744
Phosphatidylcholine (17:0_18:2) levels	CRC	rs1800588	15	58431476	T	C	0.114618	0.258046	0.019352	3.31E-09	0.014862	0.24873	0.020555	0.46967	TRUE	9.16E-09	0.00503	36.24082
Phosphatidylcholine (17:0_18:2) levels	CRC	rs964184	11	1.17E+08	C	G	-0.154483	0.848991	0.023164	2.76E-11	0.01394	0.855576	0.025002	0.577138	TRUE	7.71E-11	0.006119	44.13275
Phosphatidylcholine (17:0_20:4) levels	CRC	rs12417747	11	62060922	A	G	-0.145567	0.114819	0.026603	4.60E-08	-0.045169	0.114902	0.027589	0.101584	TRUE	2.38E-07	0.004307	30.73119
Phosphatidylcholine (17:0_20:4) levels	CRC	rs174528	11	61776027	C	T	-0.534586	0.41439	0.015932	1.00E-200	-0.075685	0.420675	0.017897	2.35E-05	TRUE	3.79E-221	0.138702	1144.017
Phosphatidylcholine (17:0_20:4) levels	CRC	rs187429064	19	19269704	G	A	-0.23955	0.053531	0.038035	3.18E-10	0.011076	0.05117	0.041499	0.789545	TRUE	6.25E-10	0.005815	41.55017
Phosphatidylcholine (17:0_20:4) levels	CRC	rs3741252	11	61744026	T	C	0.206133	0.133653	0.024645	7.20E-17	0.025172	0.129675	0.026893	0.349263	TRUE	4.61E-16	0.00984	70.59813
Phosphatidylcholine (17:0_20:4) levels	CRC	rs58983615	11	62053609	T	A	-0.328615	0.02083	0.059204	2.95E-08	0.066953	0.020833	0.062772	0.286146	TRUE	9.88E-08	0.004405	31.43232
Phosphatidylcholine (17:0_20:4) levels	CRC	rs600518	11	75744328	T	A	-0.113042	0.2323	0.01991	1.42E-08	0.018456	0.230538	0.02098	0.379026	TRUE	4.21E-08	0.004558	32.5265
Phosphatidylcholine (18:0_20:2) levels	CRC	rs174547	11	61803311	C	T	0.282264	0.407429	0.018586	4.05E-51	-0.076212	0.413977	0.017945	2.17E-05	TRUE	6.13E-47	0.038471	231.099
Phosphatidylcholine (18:0_20:4) levels	CRC	rs147549994	16	1939264	T	G	0.255782	0.035936	0.044991	1.35E-08	-0.104787	0.034779	0.048866	0.0320052	TRUE	1.15E-07	0.004533	32.66022
Phosphatidylcholine (18:0_20:4) levels	CRC	rs174528	11	61776027	C	T	-0.666205	0.41439	0.015102	1.00E-200	-0.075685	0.420675	0.017897	2.35E-05	TRUE	0	0.215409	1969.067
Phosphatidylcholine (18:0_20:4) levels	CRC	rs2797620	1	94975736	T	C	-0.101178	0.332583	0.017383	6.11E-09	0.017692	0.328894	0.018786	0.346321	TRUE	2.00E-08	0.004545	32.74296
Phosphatidylcholine (18:0_20:4) levels	CRC	rs3019200	11	61481911	A	C	-0.162646	0.803119	0.021306	2.57E-14	-0.028803	0.808536	0.022587	0.202241	TRUE	1.97E-13	0.008366	60.50471
Phosphatidylcholine (18:0_20:4) levels	CRC	rs3741252	11	61744026	T	C	0.242617	0.133653	0.024494	5.54E-23	0.025172	0.129675	0.026893	0.349263	TRUE	5.69E-22	0.013631	99.11608
Phosphatidylcholine (18:0_20:4) levels	CRC	rs4382917	11	62454004	A	G	0.148945	0.255777	0.019714	4.68E-14	0.02651	0.243412	0.020939	0.205476	TRUE	3.47E-13	0.008446	61.09002
Phosphatidylcholine (18:0_20:4) levels	CRC	rs79136768	11	61594967	G	A	-0.249044	0.036937	0.044747	2.71E-08	-0.036748	0.036049	0.04853	0.448917	TRUE	7.12E-08	0.004413	31.78772
Phosphatidylcholine (18:0_20:5) levels	CRC	rs102275	11	61790331	C	T	-0.419506	0.410377	0.016455	3.30E-137	-0.07484	0.416098	0.017929	2.99E-05	TRUE	1.06E-129	0.085166	662.7355
Phosphatidylcholine (18:0_20:5) levels	CRC	rs7936002	11	62451557	G	A	0.110547	0.257197	0.019742	2.22E-08	0.025198	0.245379	0.020852	0.226901	TRUE	8.50E-08	0.004669	33.39759
Phosphatidylcholine (18:1_18:2) levels	CRC	rs1077835	15	58431227	G	A	0.12469	0.266329	0.01915	7.94E-11	0.012205	0.257918	0.020326	0.548192	TRUE	2.23E-10	0.006076	43.84299
Phosphatidylcholine (18:1_18:2) levels	CRC	rs10889352	1	62633352	C	T	-0.126365	0.262366	0.018758	1.75E-11	0.011748	0.263349	0.020028	0.557485	TRUE	5.08E-11	0.006181	44.60309
Phosphatidylcholine (18:1_18:2) levels	CRC	rs1601935	15	58379566	T	G	-0.105723	0.611241	0.017002	5.31E-10	-0.014351	0.616613	0.018206	0.430549	TRUE	1.66E-09	0.005312	38.30146
Phosphatidylcholine (18:1_18:2) levels	CRC	rs17231506	16	56960616	T	C	0.119523	0.27829	0.01848	1.06E-10	0.02011	0.278594	0.019666	0.306512	TRUE	4.41E-10	0.005738	41.39357
Phosphatidylcholine (18:1_18:2) levels	CRC	rs174574	11	61832870	C	A	-0.380135	0.590933	0.016431	2.79E-114	0.076186	0.585489	0.017942	2.17E-05	TRUE	4.79E-107	0.069862	538.6803
Phosphatidylcholine (18:1_20:2) levels	CRC	rs12417747	11	62060922	A	G	0.18995	0.114819	0.02896	5.83E-11	-0.045169	0.114902	0.027589	0.101584	TRUE	3.55E-10	0.007334	43.22224
Phosphatidylcholine (18:1_20:2) levels	CRC	rs174580	11	61839170	G	A	0.431366	0.409729	0.017898	1.49E-122	-0.078801	0.415508	0.017921	1.10E-05	TRUE	3.19E-116	0.090006	578.6116
Phosphatidylcholine (18:1_20:2) levels	CRC	rs656095	11	75734293	G	A	-0.130437	0.226939	0.022171	4.24E-09	0.017584	0.221616	0.021256	0.408109	TRUE	1.11E-08	0.00597	35.13264
Phosphatidylcholine (18:1_20:4) levels	CRC	rs147797702	18	52743009	C	T	-0.489187	0.009355	0.089017	4.03E-08	0.007042	0.010022	0.087242	0.935665	TRUE	5.99E-08	0.004435	31.94847
Phosphatidylcholine (18:1_20:4) levels	CRC	rs174528	11	61776027	C	T	-0.499162	0.41439	0.015979	1.00E-200	-0.075685	0.420675	0.017897	2.35E-05	TRUE	1.18E-192	0.120929	986.4762
Phosphatidylcholine (18:1_20:4) levels	CRC	rs187429064	19	19269704	G	A	-0.276291	0.053531	0.037729	2.68E-13	0.011076	0.05117	0.041499	0.789545	TRUE	6.31E-13	0.007735	55.9024
Phosphatidylcholine (18:1_20:4) levels	CRC	rs2943813	11	61490996	C	T	-0.129638	0.782953	0.020413	2.26E-10	-0.025418	0.792124	0.021902	0.245834	TRUE	1.04E-09	0.005712	41.19567
Phosphatidylcholine (18:1_20:4) levels	CRC	rs3741252	11	61744026	T	C	0.186126	0.133653	0.024573	4.05E-14	0.025172	0.129675	0.026893	0.349263	TRUE	2.10E-13	0.008023	57.99527
Phosphatidylcholine (18:1_20:4) levels	CRC	rs531117	11	75745089	T	C	-0.113887	0.232379	0.019845	9.96E-09	0.018962	0.230715	0.020978	0.366049	TRUE	3.07E-08	0.004627	33.33619
Phosphatidylcholine (18:1_20:4) levels	CRC	rs8107974	19	19277691	T	A	-0.190759	0.062468	0.034384	3.00E-08	0.005684	0.063828	0.036071	0.874785	TRUE	4.78E-08	0.004262	30.69568
Phosphatidylcholine (18:2_20:3) levels	CRC	rs174548	11	61803876	G	C	0.209516	0.383631	0.017827	1.40E-31	-0.081685	0.389804	0.018124	6.58E-06	TRUE	6.47E-28	0.02076	140.1936
Phosphatidylcholine (O-16:0_20:4) levels	CRC	rs174568	11	61826344	T	C	-0.381503	0.40512	0.016461	1.16E-114	-0.075982	0.411924	0.017972	2.36E-05	TRUE	1.92E-107	0.070152	541.0871
Phosphatidylcholine (O-16:0_20:4) levels	CRC	rs3017103	11	62406721	G	A	0.115568	0.803552	0.021159	4.87E-08	-0.003202	0.809459	0.022559	0.887146	TRUE	7.59E-08	0.004217	30.36982
Phosphatidylcholine (O-16:0_20:4) levels	CRC	rs6709442	2	1.24E+08	A	G	0.094665	0.41602	0.017066	3.00E-08	-0.009812	0.427154	0.017876	0.583073	TRUE	6.64E-08	0.004354	31.36594
Phosphatidylcholine (O-16:0_22:5) levels	CRC	rs174548	11	61803876	G	C	-0.168015	0.383631	0.018886	7.57E-19	-0.081685	0.389804	0.018124	6.58E-06	TRUE	2.73E-16	0.01335	80.99425
Phosphatidylcholine (O-18:0_20:4) levels	CRC	rs174536	11	61784455	C	A	-0.308417	0.408477	0.016945	2.28E-72	-0.072915	0.414519	0.017942	4.83E-05	TRUE	7.37E-67	0.045967	335.0558
Phosphatidylcholine (O-18:0_20:4) levels	CRC	rs3741252	11	61744026	T	C	0.150025	0.133653	0.024979	1.99E-09	0.025172	0.129675	0.026893	0.349263	TRUE	6.63E-09	0.005212	36.43612
Phosphatidylcholine (O-18:1_18:2) levels	CRC	rs174551	11	61806212	C	T	0.229198	0.406164	0.017393	3.59E-39	-0.07436	0.412521	0.017954	3.45E-05	TRUE	2.88E-35	0.025341	174.6134
Phosphatidylcholine (O-18:1_20:4) levels	CRC	rs11057853	12	1.25E+08	C	T	-0.094005	0.453524	0.016839	2.45E-08	-0.017683	0.451049	0.017838	0.321535	TRUE	7.86E-08	0.00438	31.54955
Phosphatidylcholine (O-18:1_20:4) levels	CRC	rs173539	16	56954132	T	C	0.120675	0.282691	0.018459	6.68E-11	0.019012	0.283156	0.019617	0.332446	TRUE	2.69E-10	0.005906	42.6025
Phosphatidylcholine (O-18:1_20:4) levels	CRC	rs174536	11	61784455	C	A	-0.283584	0.408477	0.016741	3.74E-63	-0.072915	0.414519	0.017942	4.83E-05	TRUE	6.48E-58	0.038863	289.9526
Phosphatidylcholine (O-18:1_20:4) levels	CRC	rs1800588	15	58431476	T	C	0.107292	0.258046	0.01936	3.09E-08	0.014862	0.24873	0.020555	0.46967	TRUE	7.87E-08	0.004408	31.74952
Phosphatidylcholine (O-18:2_18:1) levels	CRC	rs174564	11	61820833	G	A	0.146557	0.409161	0.017529	7.47E-17	-0.077736	0.414913	0.017937	1.46E-05	TRUE	2.14E-14	0.010385	70.04732
Phosphatidylethanolamine (18:2_0:0) levels	CRC	rs1260326	2	27508073	C	T	-0.114725	0.650878	0.017763	1.12E-10	0.02234	0.650148	0.018596	0.229635	TRUE	5.32E-10	0.005982	41.53999
Phosphatidylethanolamine (18:2_0:0) levels	CRC	rs174560	11	61814292	C	T	0.364648	0.382915	0.01709	7.02E-98	-0.080586	0.388646	0.018138	8.87E-06	TRUE	4.41E-91	0.062838	462.8587
hosphatidylethanolamine (O-16:1_18:2) levels	CRC	rs174530	11	61779120	G	A	0.148929	0.414119	0.019284	1.33E-14	-0.073854	0.422885	0.017886	3.64E-05	TRUE	1.15E-12	0.010763	59.05579
hosphatidylethanolamine (O-18:1_18:2) levels	CRC	rs174547	11	61803311	C	T	0.194339	0.407429	0.017256	3.60E-29	-0.076212	0.413977	0.017945	2.17E-05	TRUE	9.20E-26	0.018237	127.575
hosphatidylethanolamine (O-18:1_20:4) levels	CRC	rs174536	11	61784455	C	A	-0.251165	0.408477	0.016818	1.09E-49	-0.072915	0.414519	0.017942	4.83E-05	TRUE	4.19E-45	0.030485	225.451
hosphatidylethanolamine (O-18:1_20:4) levels	CRC	rs204468	19	44987378	C	T	-0.095273	0.594995	0.017043	2.35E-08	0.00077	0.593866	0.018039	0.965957	TRUE	3.43E-08	0.004375	31.5042
hosphatidylethanolamine (O-18:2_18:2) levels	CRC	rs2727270	11	61835765	T	C	0.176894	0.2576	0.02019	2.43E-18	-0.068042	0.269346	0.020059	0.00069357	TRUE	2.70E-16	0.011969	76.73895
Phosphatidylinositol (18:0_20:3) levels	CRC	rs1168104	1	62662654	G	A	0.151178	0.737655	0.019359	6.66E-15	-0.012495	0.736517	0.020026	0.532655	TRUE	2.42E-14	0.008846	60.52709
Phosphatidylinositol (18:0_20:3) levels	CRC	rs28456	11	61822009	G	A	0.34925	0.383508	0.017256	1.70E-88	-0.081239	0.389903	0.018121	7.36E-06	TRUE	4.78E-82	0.057677	415.1096
Phosphatidylinositol (18:0_20:3) levels	CRC	rs6498540	16	15036737	G	A	-0.125793	0.334003	0.018203	5.24E-12	-0.027963	0.333744	0.018795	0.136818	TRUE	3.68E-11	0.00704	48.083
Phosphatidylinositol (18:0_20:3) levels	CRC	rs8736	19	54173495	C	T	0.222428	0.594967	0.017378	4.35E-37	-0.008012	0.605367	0.018137	0.658685	TRUE	3.91E-36	0.023845	165.6651
Phosphatidylinositol (18:0_20:3) levels	CRC	rs964184	11	1.17E+08	C	G	-0.161961	0.848991	0.02378	1.05E-11	0.01394	0.855576	0.025002	0.577138	TRUE	2.90E-11	0.006726	45.92462
Sphingomyelin (d34:2) levels	CRC	rs113127305	10	70837164	A	G	-0.217065	0.049798	0.038265	1.46E-08	0.040535	0.04467	0.042531	0.340554	TRUE	4.63E-08	0.004459	32.12294
Sphingomyelin (d34:2) levels	CRC	rs113722226	19	11078596	C	T	-0.186339	0.094288	0.028124	3.70E-11	-0.015204	0.101685	0.029552	0.606911	TRUE	9.82E-11	0.00593	42.7865
Sphingomyelin (d34:2) levels	CRC	rs116302332	4	74505174	T	C	0.29779	0.028116	0.050794	4.74E-09	0.109952	0.032989	0.049573	0.0265546	TRUE	4.62E-08	0.004846	34.92696
Sphingomyelin (d34:2) levels	CRC	rs1532085	15	58391167	G	A	-0.109151	0.573441	0.016744	7.57E-11	-0.004959	0.577767	0.017873	0.781444	TRUE	1.56E-10	0.005828	42.04673
Sphingomyelin (d34:2) levels	CRC	rs174561	11	61815236	C	T	-0.169628	0.381331	0.017171	7.26E-23	-0.076071	0.387761	0.018144	2.76E-05	TRUE	7.00E-20	0.013576	98.71028

These are selected and significantly reduced contents. SNPs showing only positive results. Readers may request the nonmodified whole contents by contacting the corresponding authors of the current study: weiguoli1987@163.com (G.W.); Huojiege@jsatcm.com (J. H.)

Table S4.Summary of all SNP-specific information in the replication dataset(Red color represents excluded outliers)

						Harmonization		Exposure				Outcome				Steiger-test			
Exposure	GWAS Catalog ID	Outcome	SNP	chr	pos	EFFECT ALLELE	OTHER ALLELE	beta.exposu	eaf.exposu	se.exposure	pval.exposure	beta.outcor	eaf.outcom	se.outcome	pval.outcome	steiger_dir	steiger_pval	R2	F
Sterol ester (27:1/18:1) levels	GCST90277245	CRC	rs11591147	1	55039974	T	G	-0.298324	0.033215	0.046602	1.63E-10	0.03165	0.02147	0.029231	0.278927	TRUE	1.31E-09	0.005716	41.22924
Sterol ester (27:1/18:1) levels	GCST90277245	CRC	rs118068660	19	11079868	T	C	-0.171924	0.094615	0.028154	1.07E-09	-0.036794	0.1115	0.025046	0.141818	TRUE	1.18E-08	0.005064	36.50419
Sterol ester (27:1/18:1) levels	GCST90277245	CRC	rs1800961	20	44413724	T	C	-0.21016	0.052411	0.037228	1.71E-08	-0.070484	0.03681	0.021734	0.00118252	TRUE	9.14E-07	0.004387	31.60251
Sterol ester (27:1/18:1) levels	GCST90277245	CRC	rs182695896	4	73947510	C	A	0.338302	0.025305	0.052958	1.78E-10	0.021062	0.01431	0.057675	0.714974	TRUE	5.97E-10	0.005646	40.72052
Sterol ester (27:1/18:1) levels	GCST90277245	CRC	rs7412	19	44908822	T	C	-0.281493	0.053066	0.037156	4.01E-14	-0.027034	0.06442	0.013873	0.0513296	TRUE	1.82E-12	0.007963	57.57234
Sterol ester (27:1/18:1) levels	GCST90277245	CRC	rs74915447	4	72899760	A	G	0.26344	0.037786	0.044273	2.80E-09	0.03789	0.02658	0.023604	0.108438	TRUE	3.32E-08	0.005047	36.37747
Sterol ester (27:1/18:3) levels	GCST90277247	CRC	rs1260326	2	27508073	C	T	-0.105379	0.650878	0.017453	1.64E-09	0.031365	0.5859	0.007515	3.00E-05	TRUE	3.15E-07	0.005047	36.37915
Sterol ester (27:1/18:3) levels	GCST90277247	CRC	rs1800961	20	44413724	T	C	-0.239553	0.052411	0.037219	1.30E-10	-0.070484	0.03681	0.021734	0.00118252	TRUE	1.32E-08	0.0057	41.11476
Sterol ester (27:1/18:3) levels	GCST90277247	CRC	rs182611493	19	19347579	G	A	-0.282368	0.053801	0.037768	8.55E-14	-0.095946	0.01329	0.044994	0.032972	TRUE	4.69E-12	0.008118	58.69704
Sterol ester (27:1/18:3) levels	GCST90277247	CRC	rs2255531	12	1.21E+08	A	G	0.093397	0.394936	0.017025	4.25E-08	0.021774	0.3681	0.007642	0.00438008	TRUE	1.37E-06	0.004169	30.02482
Sterol ester (27:1/20:3) levels	GCST90277249	CRC	rs1135999	16	15038105	G	A	-0.22595	0.315252	0.017821	1.90E-36	-0.021152	0.3037	0.008037	0.00849732	TRUE	1.39E-32	0.022042	161.5777
Sterol ester (27:1/20:3) levels	GCST90277249	CRC	rs1800961	20	44413724	T	C	-0.23874	0.052411	0.037247	1.55E-10	-0.070484	0.03681	0.021734	0.00118252	TRUE	1.54E-08	0.005661	40.81749
Sterol ester (27:1/20:3) levels	GCST90277249	CRC	rs187429064	19	19269704	G	A	-0.235892	0.053531	0.037864	4.92E-10	-0.056472	0.01329	0.040192	0.160005	TRUE	5.34E-09	0.005639	40.65234
Sterol ester (27:1/20:3) levels	GCST90277249	CRC	rs2727271	11	61835886	T	A	-0.170476	0.257859	0.019021	3.98E-19	-0.056187	0.1462	0.011409	8.44E-07	TRUE	4.97E-15	0.011123	80.63833
Sterol ester (27:1/20:3) levels	GCST90277249	CRC	rs56374730	16	15773795	G	T	0.11198	0.296257	0.018371	1.15E-09	0.002168	0.2618	0.008449	0.79749	TRUE	3.10E-09	0.005229	37.68157
Sterol ester (27:1/20:4) levels	GCST90277250	CRC	rs113248417	11	62059462	G	A	-0.267977	0.03689	0.044942	2.59E-09	-0.025174	0.02761	0.02473	0.308696	TRUE	1.61E-08	0.005103	36.78508
Sterol ester (27:1/20:4) levels	GCST90277250	CRC	rs1135999	16	15038105	G	A	-0.101484	0.315252	0.017797	1.23E-08	-0.021152	0.3037	0.008037	0.00849732	TRUE	3.72E-07	0.004446	32.0324
Sterol ester (27:1/20:4) levels	GCST90277250	CRC	rs11591147	1	55039974	T	G	-0.297311	0.033215	0.046189	1.30E-10	0.03165	0.02147	0.029231	0.278927	TRUE	1.06E-09	0.005677	40.94812
Sterol ester (27:1/20:4) levels	GCST90277250	CRC	rs182695896	4	73947510	C	A	0.295609	0.025305	0.052463	1.82E-08	0.021062	0.01431	0.057675	0.714974	TRUE	4.93E-08	0.004311	31.04966
Sterol ester (27:1/20:4) levels	GCST90277250	CRC	rs187429064	19	19269704	G	A	-0.254328	0.053531	0.037458	1.21E-11	-0.056472	0.01329	0.040192	0.160005	TRUE	1.73E-10	0.006554	47.31832
Sterol ester (27:1/20:4) levels	GCST90277250	CRC	rs2229738	11	68794860	T	C	-0.15287	0.155986	0.023409	7.01E-11	-0.040976	0.08691	0.015618	0.00870106	TRUE	3.75E-09	0.006153	44.40488
Sterol ester (27:1/20:4) levels	GCST90277250	CRC	rs3741252	11	61744026	T	C	0.212004	0.133653	0.024519	6.46E-18	-0.006404	0.09714	0.016193	0.692477	TRUE	4.75E-17	0.010409	75.43502
Sterol ester (27:1/20:4) levels	GCST90277250	CRC	rs4382917	11	62454004	A	G	0.146345	0.255777	0.019705	1.24E-13	0.016391	0.2464	0.009321	0.0786528	TRUE	3.96E-12	0.008154	58.95847
Sterol ester (27:1/20:4) levels	GCST90277250	CRC	rs7412	19	44908822	T	C	-0.309996	0.053066	0.036791	4.29E-17	-0.027034	0.06442	0.013873	0.0513296	TRUE	3.33E-15	0.009658	69.94122
Sterol ester (27:1/20:5) levels	GCST90277251	CRC	rs508049	11	68908029	T	C	-0.15839	0.097967	0.028652	3.35E-08	-0.022576	0.06748	0.0172	0.189335	TRUE	2.38E-07	0.004434	31.94172
Sterol ester (27:1/20:5) levels	GCST90277251	CRC	rs7936002	11	62451557	G	A	0.109709	0.257197	0.019671	2.53E-08	0.019371	0.2474	0.00923	0.0358416	TRUE	4.12E-07	0.004599	33.13571
Phosphatidylethanolamine (18:0_0:0) levels	GCST90277269	CRC	rs1532085	15	58391167	G	A	-0.101106	0.573441	0.017974	1.93E-08	-0.006364	0.6319	0.007563	0.400141	TRUE	7.63E-08	0.005001	31.44313
Phosphatidylethanolamine (18:0_0:0) levels	GCST90277269	CRC	rs7529794	1	39937698	T	G	0.150648	0.249805	0.020699	3.79E-13	0.016317	0.2352	0.008436	0.0531005	TRUE	1.06E-11	0.008506	53.67088
Phosphatidylethanolamine (18:2_0:0) levels	GCST90277271	CRC	rs1260326	2	27508073	C	T	-0.114725	0.650878	0.017763	1.12E-10	0.031365	0.5859	0.007515	3.00E-05	TRUE	2.94E-08	0.005982	41.53999
Phosphatidylcholine (14:0_18:1) levels	GCST90277273	CRC	rs1260326	2	27508073	C	T	-0.105277	0.650878	0.017615	2.39E-09	0.031365	0.5859	0.007515	3.00E-05	TRUE	4.16E-07	0.005037	35.75153
Phosphatidylcholine (14:0_18:2) levels	GCST90277274	CRC	rs4665972	2	27375230	C	T	-0.114678	0.620291	0.017513	6.24E-11	0.032704	0.5818	0.007646	1.89E-05	TRUE	1.96E-08	0.006195	42.78708
Phosphatidylcholine (16:0_16:0) levels	GCST90277277	CRC	rs17231506	16	56960616	T	C	0.142323	0.27829	0.018485	1.54E-14	0.013557	0.2955	0.007912	0.0866071	TRUE	5.46E-13	0.008137	58.83407
Phosphatidylcholine (16:0_16:0) levels	GCST90277277	CRC	rs261290	15	58386521	C	T	-0.138985	0.612243	0.016905	2.37E-16	-0.004702	0.6748	0.007805	0.546855	TRUE	2.06E-15	0.009172	66.38825
Phosphatidylcholine (16:0_16:0) levels	GCST90277277	CRC	rs3134950	6	32159700	A	C	0.096672	0.583482	0.016848	9.96E-09	0.02137	0.6268	0.0077	0.00551414	TRUE	3.57E-07	0.004542	32.72737
Phosphatidylcholine (16:0_16:0) levels	GCST90277277	CRC	rs73015021	19	11082239	G	A	-0.158246	0.092126	0.028665	3.49E-08	-0.025822	0.1104	0.011493	0.024654	TRUE	6.38E-07	0.004189	30.16948
Phosphatidylcholine (16:0_16:1) levels	GCST90277278	CRC	rs1260326	2	27508073	C	T	-0.108205	0.650878	0.017465	6.12E-10	0.031365	0.5859	0.007515	3.00E-05	TRUE	1.37E-07	0.005321	38.35639
Phosphatidylcholine (16:0_18:0) levels	GCST90277280	CRC	rs174601	11	61855668	T	C	0.146355	0.419025	0.01691	6.00E-18	-0.041801	0.3947	0.007689	5.42E-08	TRUE	1.08E-13	0.010429	75.37426
Phosphatidylcholine (16:1_18:0) levels	GCST90277292	CRC	rs1260326	2	27508073	C	T	-0.107928	0.650878	0.018284	3.75E-09	0.031365	0.5859	0.007515	3.00E-05	TRUE	4.97E-07	0.005294	34.71052
Phosphatidylcholine (18:0_20:5) levels	GCST90277305	CRC	rs7936002	11	62451557	G	A	0.110547	0.257197	0.019742	2.22E-08	0.019371	0.2474	0.00923	0.0358416	TRUE	3.63E-07	0.004669	33.39759
Phosphatidylcholine (18:0_22:5) levels	GCST90277306	CRC	rs1260326	2	27508073	C	T	-0.101199	0.650878	0.017903	1.64E-08	0.031365	0.5859	0.007515	3.00E-05	TRUE	1.91E-06	0.004654	31.81162
Phosphatidylcholine (18:1_18:1) levels	GCST90277308	CRC	rs10889352	1	62633352	C	T	-0.112327	0.262366	0.018843	2.61E-09	-0.012324	0.2986	0.007825	0.115259	TRUE	3.02E-08	0.004884	35.19761
Phosphatidylcholine (18:1_18:1) levels	GCST90277308	CRC	rs1532085	15	58391167	G	A	-0.109594	0.573441	0.016805	7.42E-11	-0.006364	0.6319	0.007563	0.400141	TRUE	4.67E-10	0.005876	42.39075
Phosphatidylcholine (18:1_18:1) levels	GCST90277308	CRC	rs17231506	16	56960616	T	C	0.10933	0.27829	0.018562	4.04E-09	0.013557	0.2955	0.007912	0.0866071	TRUE	5.21E-08	0.004801	34.60187
Phosphatidylcholine (O-16:1_16:0) levels	GCST90277325	CRC	rs1077989	14	67509105	C	A	-0.182775	0.467505	0.018317	2.91E-23	-0.021272	0.4468	0.008039	0.00814285	TRUE	1.03E-20	0.016633	99.21828
Phosphatidylcholine (O-16:1_20:4) levels	GCST90277330	CRC	rs1077989	14	67509105	C	A	-0.191986	0.467505	0.01805	3.43E-26	-0.021272	0.4468	0.008039	0.00814285	TRUE	2.16E-23	0.018351	112.5972
Phosphatidylcholine (O-16:1_20:4) levels	GCST90277330	CRC	rs2043085	15	58388755	C	T	-0.09993	0.576127	0.018277	4.75E-08	-0.004294	0.6288	0.007655	0.574846	TRUE	1.32E-07	0.004877	29.5194
Phosphatidylcholine (O-18:1_18:2) levels	GCST90277338	CRC	rs174551	11	61806212	C	T	0.229198	0.406164	0.017393	3.59E-39	-0.047124	0.3528	0.014787	0.00143841	TRUE	9.80E-35	0.025341	174.6314
Phosphatidylcholine (O-18:1_20:3) levels	GCST90277339	CRC	rs6498540	16	15036737	G	A	-0.109283	0.334003	0.018179	1.93E-09	-0.021866	0.3098	0.008027	0.00644843	TRUE	7.24E-08	0.005313	36.50451
Phosphatidylcholine (O-18:2_20:4) levels	GCST90277344	CRC	rs1980615	14	67491655	C	A	-0.103279	0.455123	0.018056	1.12E-08	-0.024592	0.4141	0.008084	0.00235018	TRUE	3.80E-07	0.00529	32.5755
hosphatidylethanolamine (O-18:2_18:2) leve	GCST90277356	CRC	rs2727270	11	61835765	T	C	0.176894	0.2576	0.02019	2.43E-18	-0.056003	0.1462	0.011402	9.02E-07	TRUE	1.31E-14	0.011969	76.73895
Phosphatidylinositol (16:0_20:4) levels	GCST90277360	CRC	rs1260326	2	27508073	C	T	-0.109504	0.650878	0.019913	3.99E-08	0.031365	0.5859	0.007515	3.00E-05	TRUE	2.54E-06	0.00545	30.23034
Phosphatidylinositol (16:0_20:4) levels	GCST90277360	CRC	rs9653945	3	1.43E+08	A	G	-0.110791	0.364111	0.019903	2.72E-08	0.014109	0.3916	0.007762	0.0691052	TRUE	2.33E-07	0.005684	31.53789
Phosphatidylinositol (18:0_20:3) levels	GCST90277363	CRC	rs1168104	1	62662654	G	A	0.151178	0.737655	0.019359	6.66E-15	0.01127	0.6963	0.007816	0.149329	TRUE	1.50E-13	0.008846	60.52709
Phosphatidylinositol (18:0_20:3) levels	GCST90277363	CRC	rs6498540	16	15036737	G	A	-0.125793	0.334003	0.018203	5.24E-12	-0.021866	0.3098	0.008027	0.00644843	TRUE	3.66E-10	0.00704	48.083
Phosphatidylinositol (18:0_20:3) levels	GCST90277363	CRC	rs8736	19	54173495	C	T	0.222428	0.594967	0.017378	4.35E-37	0.011349	0.5644	0.007489	0.129657	TRUE	1.99E-34	0.023845	165.6651
Phosphatidylinositol (18:0_20:3) levels	GCST90277363	CRC	rs964184	11	1.17E+08	C	G	-0.161961	0.848991	0.02378	1.05E-11	-0.001871	0.8364	0.010616	0.860073	TRUE	2.96E-11	0.006726	45.92462
Phosphatidylinositol (18:0_20:4) levels	GCST90277364	CRC	rs1168104	1	62662654	G	A	0.193608	0.737655	0.018705	6.30E-25	0.01127	0.6963	0.007816	0.149329	TRUE	6.68E-23	0.014508	105.582
Phosphatidylinositol (18:0_20:4) levels	GCST90277364	CRC	rs1260326	2	27508073	C	T	-0.096388	0.650878	0.017386	3.06E-08	0.031365	0.5859	0.007515	3.00E-05	TRUE	3.64E-06	0.004222	30.411
Phosphatidylinositol (18:0_20:4) levels	GCST90277364	CRC	rs17699030	19	11220266	G	A	-0.23075	0.046946	0.039296	4.48E-09	-0.017314	0.03885	0.020008	0.386826	TRUE	2.27E-08	0.004765	34.33548
Phosphatidylinositol (18:0_20:4) levels	GCST90277364	CRC	rs56339947	18	49628409	C	T	0.18819	0.114435	0.026504	1.36E-12	0.017425	0.08691	0.014789	0.238709	TRUE	1.68E-11	0.007178	51.85269
Phosphatidylinositol (18:0_20:4) levels	GCST90277364	CRC	rs8736	19	54173495	C	T	0.275272	0.594967	0.016748	1.26E-59	0.011349	0.5644	0.007489	0.129657	TRUE	8.74E-56	0.036521	271.8537
Phosphatidylinositol (18:0_20:4) levels	GCST90277364	CRC	rs964184	11	1.17E+08	C	G	-0.267072	0.848991	0.022979	5.97E-31	-0.001871	0.8364	0.010616	0.860073	TRUE	8.91E-30	0.018289	133.6132
Sphingomyelin (d42:2) levels	GCST90277378	CRC	rs112585713	4	72876701	A	G	0.28818	0.03164	0.047895	1.86E-09	0.046229	0.0184	0.029435	0.116296	TRUE	2.20E-08	0.005089	36.68476
Sphingomyelin (d42:2) levels	GCST90277378	CRC	rs114483871	4	73124451	T	C	0.279372	0.038097	0.044809	4.78E-10	0.014991	0.02454	0.020379	0.461973	TRUE	2.37E-09	0.00572	41.26203
Sphingomyelin (d42:2) levels	GCST90277378	CRC	rs11591147	1	55039974	T	G	-0.361828	0.033215	0.04651	8.29E-15	0.03165	0.02147	0.029231	0.278927	TRUE	1.23E-13	0.008408	60.81503
Sphingomyelin (d42:2) levels	GCST90277378	CRC	rs118068660	19	11079868	T	C	-0.21909	0.094615	0.02807	6.77E-15	-0.036794	0.1115	0.025046	0.141818	TRUE	1.79E-13	0.008224	59.46976

Sphingomyelin (d42:2) levels	GCST90277378	CRC	rs146331166	17	5464230	C	T	-0.218616	0.059584	0.034523	2.56E-10	-0.025819	0.02147	0.027391	0.345867	TRUE	1.68E-09	0.005356	38.62041
Sphingomyelin (d42:2) levels	GCST90277378	CRC	rs17145750	7	73612048	T	C	0.132228	0.140658	0.023854	3.07E-08	0.01722	0.1472	0.010134	0.0892651	TRUE	3.27E-07	0.004227	30.44298
Sphingomyelin (d42:2) levels	GCST90277378	CRC	rs182695896	4	73947510	C	A	0.38203	0.025305	0.052847	5.36E-13	0.021062	0.01431	0.057675	0.714974	TRUE	2.32E-12	0.007199	52.00897
Sphingomyelin (d42:2) levels	GCST90277378	CRC	rs7412	19	44908822	T	C	-0.210972	0.053066	0.037185	1.45E-08	-0.027034	0.06442	0.013873	0.0513296	TRUE	2.14E-07	0.004473	32.22575
Sphingomyelin (d42:2) levels	GCST90277378	CRC	rs75679663	17	4764677	A	C	-0.537754	0.025788	0.05341	1.09E-23	-0.015069	0.01636	0.03689	0.68292	TRUE	1.42E-22	0.01453	105.7467
Triacylglycerol (46:2) levels	GCST90277380	CRC	rs1260326	2	27508073	C	T	-0.113769	0.650878	0.020003	1.35E-08	0.031365	0.5859	0.007515	3.00E-05	TRUE	9.81E-07	0.005882	32.22512

These are selected and significantly reduced contents. SNPs showing only positive results. Readers may request the nonmodified whole contents by contacting the corresponding authors of the current study: weiguoli1987@163.com (G.W.); Huojiege@jsatcm.com (J. H.)

Table S5. Summary of positive results in both datasets

exposure	outcome	method	nsnp	b	se	pval	or	or_lci95	or_uci95
**Discovery Dataset**									
Sterol ester (27:1/20:4) levels	CRC	IVW	10	0.112198	0.024322	3.97E-06	1.118734	1.066655	1.173356
Sterol ester (27:1/20:5) levels	CRC	IVW	3	0.19416	0.041611	3.07E-06	1.214291	1.119187	1.317476
Phosphatidylcholine (20:4_0:0) levels	CRC	IVW	4	0.137572	0.032207	1.94E-05	1.147485	1.077288	1.222256
Phosphatidylethanolamine (18:2_0:0) levels	CRC	IVW	2	-0.218736	0.047552	4.23E-06	0.803534	0.732027	0.882026
Phosphatidylcholine (14:0_18:2) levels	CRC	IVW	3	-0.292757	0.115681	0.011383	0.746204	0.594823	0.936111
Phosphatidylcholine (15:0_18:2) levels	CRC	IVW	6	-0.151126	0.059576	0.01119	0.859739	0.764988	0.966226
Phosphatidylcholine (16:0_18:0) levels	CRC	Wald ratio	1	-0.491969	0.122337	5.78E-05	0.611421	0.481067	0.777098
Phosphatidylcholine (16:0_18:2) levels	CRC	IVW	7	-0.127123	0.056015	0.023241	0.880625	0.789061	0.982815
Phosphatidylcholine (16:0_20:1) levels	CRC	Wald ratio	1	-0.658609	0.156282	2.51E-05	0.517571	0.381013	0.703071
Phosphatidylcholine (16:0_20:2) levels	CRC	IVW	6	-0.195421	0.06242	0.001744	0.822488	0.727775	0.929528
Phosphatidylcholine (16:0_20:4) levels	CRC	IVW	4	0.12584	0.028774	1.22E-05	1.134101	1.07191	1.1999
Phosphatidylcholine (16:0_20:5) levels	CRC	Wald ratio	1	0.192537	0.048172	6.42E-05	1.212322	1.103095	1.332364
Phosphatidylcholine (16:0_22:4) levels	CRC	IVW	2	0.271775	0.058483	3.37E-06	1.312292	1.170168	1.471677
Phosphatidylcholine (16:0_22:5) levels	CRC	IVW	4	0.166546	0.039739	2.78E-05	1.181218	1.092707	1.276898
Phosphatidylcholine (16:0_22:6) levels	CRC	Wald ratio	1	0.619762	0.143265	1.52E-05	1.858486	1.403491	2.460986
Phosphatidylcholine (16:1_18:1) levels	CRC	IVW	5	-0.263559	0.093872	0.00499	0.768313	0.639194	0.923513
Phosphatidylcholine (16:1_18:2) levels	CRC	IVW	8	-0.146295	0.044865	0.001111	0.863903	0.791179	0.943312
Phosphatidylcholine (16:1_20:4) levels	CRC	Wald ratio	1	0.227918	0.054736	3.13E-05	1.255982	1.128214	1.39822
Phosphatidylcholine (17:0_18:2) levels	CRC	IVW	3	-0.217182	0.099897	0.029701	0.804783	0.661675	0.978843
Phosphatidylcholine (17:0_20:4) levels	CRC	IVW	6	0.121906	0.037574	0.001177	1.129648	1.049446	1.21598
Phosphatidylcholine (18:0_20:2) levels	CRC	Wald ratio	1	-0.270001	0.063576	2.17E-05	0.763379	0.673943	0.864683
Phosphatidylcholine (18:0_20:4) levels	CRC	IVW	7	0.103884	0.032103	0.001213	1.109472	1.041812	1.181525
Phosphatidylcholine (18:0_20:5) levels	CRC	IVW	2	0.180818	0.041682	1.44E-05	1.198197	1.104201	1.300195
Phosphatidylcholine (18:1_18:2) levels	CRC	IVW	5	-0.132653	0.06438	0.039354	0.875769	0.771948	0.993553
Phosphatidylcholine (18:1_20:2) levels	CRC	IVW	3	-0.183896	0.038795	2.13E-06	0.832022	0.771103	0.897755
Phosphatidylcholine (18:1_20:4) levels	CRC	IVW	7	0.124063	0.031718	9.17E-05	1.132087	1.063852	1.2047
Phosphatidylcholine (18:2_20:3) levels	CRC	Wald ratio	1	-0.389877	0.086505	6.57E-06	0.67714	0.571537	0.802256
Phosphatidylcholine (O-16:0_20:4) levels	CRC	IVW	3	0.170552	0.058949	0.003813	1.18596	1.056554	1.331215
Phosphatidylcholine (O-16:0_22:5) levels	CRC	Wald ratio	1	0.486179	0.107872	6.57E-06	1.626091	1.316202	2.008942
Phosphatidylcholine (O-18:0_20:4) levels	CRC	IVW	2	0.229877	0.055334	3.26E-05	1.258445	1.129102	1.402604
Phosphatidylcholine (O-18:1_18:2) levels	CRC	Wald ratio	1	-0.324436	0.078332	3.45E-05	0.722935	0.620043	0.842902
Phosphatidylcholine (O-18:1_20:4) levels	CRC	IVW	4	0.2311	0.05402	1.89E-05	1.259985	1.133397	1.400711
Phosphatidylcholine (O-18:2_18:1) levels	CRC	Wald ratio	1	-0.530418	0.122387	1.46E-05	0.588359	0.462876	0.74786
Phosphatidylethanolamine (O-16:1_18:2) levels	CRC	Wald ratio	1	-0.495903	0.120097	3.64E-05	0.60902	0.481286	0.770656

Phosphatidylethanolamine (O-18:1_18:2) levels	CRC	Wald ratio	1	-0.392158	0.09234	2.17E-05	0.675598	0.563751	0.809635
Phosphatidylethanolamine (O-18:1_20:4) levels	CRC	IVW	2	0.253125	0.098549	0.010214	1.288045	1.061803	1.562492
Phosphatidylethanolamine (O-18:2_18:2) levels	CRC	Wald ratio	1	-0.384647	0.113394	0.000694	0.680691	0.545038	0.850107
Phosphatidylinositol (18:0_20:3) levels	CRC	IVW	5	-0.135495	0.06424	0.034928	0.873283	0.769968	0.990461
Sphingomyelin (d34:2) levels	CRC	IVW	5	0.232115	0.112835	0.039675	1.261265	1.011019	1.573452
**Replication Dataset**									
Sterol ester (27:1/18:1) levels	CRC	IVW	6	0.110356	0.050562	0.029067	1.116675	1.011317	1.233009
Sterol ester (27:1/18:3) levels	CRC	Wald ratio	1	0.33979	0.159344	0.032972	1.404652	1.027856	1.919576
Sterol ester (27:1/20:3) levels	CRC	IVW	5	0.144932	0.053685	0.00694	1.155961	1.040509	1.284223
Sterol ester (27:1/20:4) levels	CRC	IVW	9	0.092943	0.032799	0.0046	1.097399	1.029073	1.170263
Sterol ester (27:1/20:5) levels	CRC	IVW	2	0.163804	0.066508	0.013781	1.177983	1.034015	1.341997
Phosphatidylethanolamine (18:0_0:0) levels	CRC	IVW	2	0.092015	0.04483	0.040117	1.096382	1.004157	1.197076
Phosphatidylethanolamine (18:2_0:0) levels	CRC	Wald ratio	1	-0.273396	0.065505	3.00E-05	0.760792	0.669124	0.865017
Phosphatidylcholine (14:0_18:1) levels	CRC	Wald ratio	1	-0.297931	0.071384	3.00E-05	0.742352	0.645427	0.853834
Phosphatidylcholine (14:0_18:2) levels	CRC	Wald ratio	1	-0.285182	0.066671	1.89E-05	0.751877	0.659774	0.856838
Phosphatidylcholine (16:0_16:0) levels	CRC	IVW	4	0.108648	0.038479	0.004749	1.11477	1.033788	1.202096
Phosphatidylcholine (16:0_16:1) levels	CRC	Wald ratio	1	-0.289869	0.069452	3.00E-05	0.748361	0.653119	0.857492
Phosphatidylcholine (16:0_18:0) levels	CRC	Wald ratio	1	-0.285616	0.052533	5.42E-08	0.751551	0.678018	0.833059
Phosphatidylcholine (16:1_18:0) levels	CRC	Wald ratio	1	-0.290613	0.06963	3.00E-05	0.747805	0.652406	0.857154
Phosphatidylcholine (18:0_20:5) levels	CRC	Wald ratio	1	0.17523	0.083494	0.035841	1.191521	1.01165	1.403373
Phosphatidylcholine (18:0_22:5) levels	CRC	Wald ratio	1	-0.309937	0.07426	3.00E-05	0.733493	0.634139	0.848414
Phosphatidylcholine (18:1_18:1) levels	CRC	IVW	3	0.096344	0.040589	0.017613	1.101138	1.016931	1.192317
Phosphatidylcholine (O-16:1_16:0) levels	CRC	Wald ratio	1	0.116384	0.043983	0.008143	1.123427	1.030636	1.224572
Phosphatidylcholine (O-16:1_20:4) levels	CRC	IVW	2	0.095197	0.036743	0.009572	1.099876	1.023452	1.182006
Phosphatidylcholine (O-18:1_18:2) levels	CRC	Wald ratio	1	-0.205603	0.064517	0.001438	0.814156	0.717447	0.923901
Phosphatidylcholine (O-18:1_20:3) levels	CRC	Wald ratio	1	0.200088	0.073452	0.006449	1.22151	1.057726	1.410655
Phosphatidylcholine (O-18:2_20:4) levels	CRC	Wald ratio	1	0.238113	0.078275	0.00235	1.268853	1.088384	1.479247
Phosphatidylethanolamine (O-18:2_18:2) levels	CRC	Wald ratio	1	-0.316593	0.064454	9.02E-07	0.728627	0.642156	0.826742
Phosphatidylinositol (16:0_20:4) levels	CRC	IVW	2	-0.208526	0.079526	0.008739	0.81178	0.694615	0.948708
Phosphatidylinositol (18:0_20:3) levels	CRC	IVW	4	0.068187	0.026887	0.011212	1.070566	1.015608	1.128497
Phosphatidylinositol (18:0_20:4) levels	CRC	IVW	5	0.041773	0.018593	0.024661	1.042658	1.005344	1.081357
Sphingomyelin (d42:2) levels	CRC	IVW	9	0.074983	0.028454	0.008408	1.077866	1.019399	1.139687
Triacylglycerol (46:2) levels	CRC	Wald ratio	1	-0.275693	0.066056	3.00E-05	0.759046	0.666869	0.863964

Table. S6 Summary of meta-analysis results for replication and discovery datasets(Red represents the discovery of significant causal evidence in the dataset)

Phenotype	Discovery dataset		Replication dataset		Meta-analysis				Whether evidence of causation is established	
	$OR_{IVW/Wald\ ratio}$(95%CI)	Pval	$OR_{IVW/Wald\ ratio}$(95%CI)	Pval	I^2(%)	Model	OR (95% CI)	Pval		
Sterol ester (27:1/20:4) levels	1.119 (1.067, 1.173)	3.97E-06	1.097 (1.029, 1.170)	0.0046	0	Fixed	1.111 (1.070, 1.154)	5.88E-08	YES	Significant
Sterol ester (27:1/20:5) levels	1.214 (1.119, 1.317)	3.07E-06	1.178 (1.034, 1.342)	0.01378	0	Fixed	1.204 (1.123, 1.290)	1.42E-07	YES	Significant
Phosphatidylcholine (20:4_0:0) levels	1.147 (1.077, 1.222)	1.94E-05	1.084 (0.976, 1.203)	0.13179	0	Fixed	1.130 (1.070, 1.193)	9.64E-06	YES	Significant
Phosphatidylcholine (14:0_18:2) levels	0.746 (0.595, 0.936)	0.011	0.752 (0.660, 0.857)	1.89E-05	0	Fixed	0.750 (0.670, 0.840)	6.62E-07	YES	Suggestive
Phosphatidylcholine (15:0_18:2) levels	0.860 (0.765, 0.966)	0.01119	1.025 (0.931, 1.128)	0.6126	81	Random	0.942 (0.793, 1.119)	0.495	NO	exclude
Phosphatidylcholine (16:0_18:0) levels	0.611 (0.481, 0.777)	5.78E-05	0.752 (0.678, 0.833)	5.42E-08	59	Random	0.698 (0.574, 0.848)	3.06E-04	YES	Significant
Phosphatidylcholine (16:0_18:2) levels	0.881 (0.789, 0.983)	0.02324	1.017 (0.942, 1.097)	0.66912	77	Random	0.952 (0.827, 1.095)	0.491	NO	exclude
Phosphatidylcholine (16:0_20:1) levels	0.518 (0.381, 0.703)	2.51E-05	NA	NA	NA	NA	NA	NA	YES	Suggestive
Phosphatidylcholine (16:0_20:2) levels	0.822 (0.728, 0.930)	0.00174	0.989 (0.916, 1.068)	0.78657	84	Random	0.907 (0.757, 1.087)	0.292	NO	exclude
Phosphatidylcholine (16:0_20:4) levels	1.134 (1.072, 1.200)	1.22E-05	0.992 (0.897, 1.096)	0.86983	81	Random	1.068 (0.973, 1.217)	0.325	NO	exclude
Phosphatidylcholine (16:0_20:5) levels	1.212 (1.103, 1.332)	6.42E-05	NA	NA	NA	NA	NA	NA	YES	Suggestive
Phosphatidylcholine (16:0_22:4) levels	1.312 (1.170, 1.472)	3.37E-06	1.077 (0.845, 1.372)	0.54873	52	Random	1.225 (1.019, 1.473)	0.031	YES	Significant
Phosphatidylcholine (16:0_22:5) levels	1.181 (1.093, 1.277)	2.78E-05	1.122 (0.981, 1.284)	0.0926	0	Fixed	1.166 (1.090, 1.247)	7.90E-06	YES	Significant
Phosphatidylcholine (16:0_22:6) levels	1.858 (1.403, 2.461)	1.52E-05	NA	NA	NA	NA	NA	NA	YES	Suggestive
Phosphatidylcholine (16:1_18:1) levels	0.768 (0.639, 0.924)	0.00499	0.926 (0.791, 1.083)	0.33357	56	Random	0.849 (0.707, 1.019)	0.079	NO	exclude
Phosphatidylcholine (16:1_18:2) levels	0.864 (0.791, 0.943)	0.00111	0.965 (0.886, 1.052)	0.41606	68	Random	0.913 (0.820, 1.018)	0.102	NO	exclude
Phosphatidylcholine (16:1_20:4) levels	1.256 (1.128, 1.398)	3.13E-05	NA	NA	NA	NA	NA	NA	YES	Suggestive
Phosphatidylcholine (17:0_18:2) levels	0.805 (0.662, 0.979)	0.0297	0.992 (0.897, 1.098)	0.88334	71	Random	0.909 (0.743, 1.113)	0.356	NO	exclude
Phosphatidylcholine (17:0_20:4) levels	1.130 (1.049, 1.216)	0.00118	1.036 (0.954, 1.125)	0.40241	58	Random	1.084 (0.996, 1.180)	0.063	NO	exclude
Phosphatidylcholine (18:0_20:2) levels	0.763 (0.674, 0.865)	2.17E-05	NA	NA	NA	NA	NA	NA	YES	Suggestive
Phosphatidylcholine (18:0_20:4) levels	1.109 (1.042, 1.182)	0.00121	1.014 (0.953, 1.078)	0.66874	75	Random	1.060 (0.971, 1.157)	0.192	NO	exclude
Phosphatidylcholine (18:0_20:5) levels	1.198 (1.104, 1.300)	1.44E-05	1.192 (1.012, 1.403)	0.03584	0	Fixed	1.197 (1.112, 1.288)	1.45E-06	YES	Significant
Phosphatidylcholine (18:1_18:2) levels	0.876 (0.772, 0.994)	0.03935	1.035 (0.943, 1.136)	0.46999	77	Random	0.958 (0.814, 1.127)	0.603	NO	exclude
Phosphatidylcholine (18:1_20:2) levels	0.832 (0.771, 0.898)	2.13E-06	0.928 (0.851, 1.012)	0.08991	71	Random	0.877 (0.788, 0.976)	0.016	YES	Significant
Phosphatidylcholine (18:1_20:4) levels	1.132 (1.064, 1.205)	9.17E-05	0.991 (0.928, 1.057)	0.779	88	Random	1.060 (0.930, 1.207)	0.385	NO	exclude
Phosphatidylcholine (18:2_20:3) levels	0.677 (0.572, 0.802)	6.57E-06	NA	NA	NA	NA	NA	NA	YES	Suggestive
Phosphatidylcholine (O-16:0_20:4) levels	1.186 (1.057, 1.331)	0.00381	1.021 (0.913, 1.141)	0.72019	70	Random	1.100 (0.949, 1.273)	0.205	NO	exclude
Phosphatidylcholine (O-16:0_22:5) levels	1.626 (1.316, 2.009)	6.57E-06	NA	NA	NA	NA	NA	NA	YES	Suggestive
Phosphatidylcholine (O-18:0_20:4) levels	1.258 (1.129, 1.403)	3.26E-05	0.958 (0.776, 1.184)	0.69248	80	Random	1.115 (0.855, 1.454)	0.421	NO	exclude
Phosphatidylcholine (O-18:1_18:2) levels	0.723 (0.620, 0.843)	3.45E-05	0.814 (0.717, 0.924)	0.00144	27	Fixed	0.776 (0.704, 0.856)	3.63E-07	YES	Significant
Phosphatidylcholine (O-18:1_20:4) levels	1.260 (1.133, 1.401)	1.89E-05	0.997 (0.864, 1.152)	0.97157	85	Random	1.127 (0.896, 1.417)	0.308	NO	exclude
Phosphatidylcholine (O-18:2_18:1) levels	0.588 (0.463, 0.748)	1.46E-05	NA	NA	NA	NA	NA	NA	YES	Suggestive
Phosphatidylethanolamine (18:2_0:0) levels	0.804 (0.732, 0.882)	4.23E-06	0.761 (0.669, 0.865)	3.00E-05	0	Fixed	0.789 (0.732, 0.851)	7.27E-10	YES	Significant
Phosphatidylethanolamine (O-16:1_18:2) levels	0.609 (0.481, 0.771)	3.64E-05	NA	NA	NA	NA	NA	NA	YES	Suggestive
Phosphatidylethanolamine (O-18:1_18:2) levels	0.676 (0.564, 0.810)	2.17E-05	NA	NA	NA	NA	NA	NA	YES	Suggestive
Phosphatidylethanolamine (O-18:1_20:4) levels	1.288 (1.062, 1.562)	0.01021	1.160 (0.991, 1.357)	0.06528	0	Fixed	1.209 (1.071, 1.366)	0.002	YES	Suggestive
Phosphatidylethanolamine (O-18:2_18:2) levels	0.681 (0.545, 0.850)	0.00069	0.729 (0.642, 0.827)	9.02E-07	0	Fixed	0.717 (0.642, 0.800)	3.05E-09	YES	Suggestive
Phosphatidylinositol (18:0_20:3) levels	0.873 (0.770, 0.990)	0.03493	1.071 (1.016, 1.128)	0.01121	88	Random	0.975 (0.799, 1.190)	0.804	NO	exclude
Sphingomyelin (d34:2) levels	1.261 (1.011, 1.573)	0.03967	1.043 (0.947, 1.149)	0.39659	58	Random	1.116 (0.934, 1.334)	0.227	NO	exclude

Table S7.Sensitivity analysis summary in the discovery dataset

Exposure	GWAS Catalog ID	MR-Egger intercept			Cochrane's Q-IVW			MR-PRESSO global test		
		Intercept	SE	*Pval*	*Q*	*Q_df*	*Q_pval*	RSS_{obs}	*P-value*	*Outlier*
Sterol ester (27:1/14:0) levels	GCST90277238	NA	NA	NA	2.302	1	0.129	NA	NA	NA
Sterol ester (27:1/16:0) levels	GCST90277240	-0.003	0.039	0.940	11.855	7	0.105	15.434	0.123	rs174547
Sterol ester (27:1/16:1) levels	GCST90277241	-0.059	0.133	0.733	1.004	2	0.605	NA	NA	NA
Sterol ester (27:1/17:0) levels	GCST90277242	NA	NA	NA	6.975	1	0.008	NA	NA	NA
Sterol ester (27:1/18:0) levels	GCST90277244	0.016	0.029	0.607	7.189	5	0.207	9.888	0.278	rs4246215,rs429358
Sterol ester (27:1/18:1) levels	GCST90277245	0.018	0.092	0.854	6.659	5	0.247	9.533	0.288	NA
Sterol ester (27:1/18:2) levels	GCST90277246	0.005	0.031	0.869	11.874	7	0.105	15.345	0.159	rs2524299
Sterol ester (27:1/18:3) levels	GCST90277247	0.005	0.060	0.938	14.867	4	0.005	27.480	0.054	NA
Sterol ester (27:1/20:2) levels	GCST90277248	-0.189	0.073	0.234	7.125	2	0.028	NA	NA	NA
Sterol ester (27:1/20:3) levels	GCST90277249	-0.006	0.064	0.926	11.249	5	0.047	15.927	0.080	NA
Sterol ester (27:1/20:4) levels	GCST90277250	0.010	0.015	0.513	7.613	9	0.574	8.486	0.718	NA
Sterol ester (27:1/20:5) levels	GCST90277251	0.002	0.026	0.957	0.075	2	0.963	NA	NA	NA
Sterol ester (27:1/22:6) levels	GCST90277252	NA	NA	NA	4.644	1	0.031	NA	NA	NA
Ceramide (d40:1) levels	GCST90277253	-0.004	0.027	0.884	3.393	5	0.640	4.076	0.737	NA
Ceramide (d40:2) levels	GCST90277254	NA	NA	NA	1.373	1	0.241	NA	NA	NA
Ceramide (d42:1) levels	GCST90277255	0.041	0.073	0.678	0.419	2	0.811	NA	NA	NA
Ceramide (d42:2) levels	GCST90277256	-0.028	0.022	0.247	3.311	7	0.855	4.311	0.867	NA
Diacylglycerol (16:1_18:1) levels	GCST90277260	NA	NA	NA	0.258	1	0.611	NA	NA	NA
Diacylglycerol (18:1_18:1) levels	GCST90277261	-0.013	0.033	0.730	4.162	4	0.385	5.501	0.512	NA
Diacylglycerol (18:1_18:2) levels	GCST90277262	-0.049	0.025	0.120	6.045	5	0.302	7.834	0.398	NA
Phosphatidylcholine (16:0_0:0) levels	GCST90277264	NA	NA	NA	0.964	1	0.326	NA	NA	NA
Phosphatidylcholine (18:2_0:0) levels	GCST90277267	-0.175	0.247	0.608	20.280	2	0.000	NA	NA	NA
Phosphatidylcholine (20:4_0:0) levels	GCST90277268	-0.013	0.021	0.613	3.126	3	0.373	4.886	0.627	NA
Phosphatidylethanolamine (18:0_0:0) levels	GCST90277269	NA	NA	NA	0.027	1	0.869	NA	NA	NA
Phosphatidylethanolamine (18:2_0:0) levels	GCST90277271	NA	NA	NA	0.024	1	0.877	NA	NA	NA
Phosphatidylcholine (14:0_18:1) levels	GCST90277273	NA	NA	NA	0.364	1	0.546	NA	NA	NA
Phosphatidylcholine (14:0_18:2) levels	GCST90277274	-0.036	0.056	0.631	5.473	2	0.065	NA	NA	NA
Phosphatidylcholine (15:0_18:2) levels	GCST90277276	0.041	0.018	0.085	8.755	5	0.119	25.495	0.252	NA
Phosphatidylcholine (16:0_16:0) levels	GCST90277277	-0.122	0.100	0.348	10.391	3	0.016	20.469	0.079	NA
Phosphatidylcholine (16:0_18:1) levels	GCST90277281	NA	NA	NA	1.096	1	0.295	NA	NA	NA
Phosphatidylcholine (16:0_18:2) levels	GCST90277282	0.047	0.017	0.042	11.188	6	0.083	28.704	0.168	NA
Phosphatidylcholine (16:0_18:3) levels	GCST90277283	0.550	0.882	0.645	9.389	2	0.009	NA	NA	NA
Phosphatidylcholine (16:0_20:2) levels	GCST90277285	0.029	0.019	0.200	7.018	5	0.219	17.019	0.279	NA
Phosphatidylcholine (16:0_20:3) levels	GCST90277286	NA	NA	NA	4.342	1	0.037	NA	NA	NA
Phosphatidylcholine (16:0_20:4) levels	GCST90277287	-0.001	0.024	0.956	1.051	3	0.789	1.942	0.869	NA
Phosphatidylcholine (16:0_22:4) levels	GCST90277289	NA	NA	NA	0.547	1	0.459	NA	NA	NA
Phosphatidylcholine (16:0_22:5) levels	GCST90277290	-0.014	0.025	0.622	0.402	3	0.940	2.545	0.793	NA
Phosphatidylcholine (16:1_18:1) levels	GCST90277293	0.039	0.039	0.390	8.035	4	0.090	16.383	0.161	NA
Phosphatidylcholine (16:1_18:2) levels	GCST90277294	0.026	0.014	0.109	9.106	7	0.245	20.857	0.292	NA
Phosphatidylcholine (17:0_18:2) levels	GCST90277297	0.085	0.036	0.256	5.531	2	0.063	NA	NA	NA
Phosphatidylcholine (17:0_20:4) levels	GCST90277298	-0.022	0.025	0.430	7.543	5	0.183	20.275	0.394	NA
Phosphatidylcholine (18:0_18:1) levels	GCST90277299	NA	NA	NA	3.908	1	0.048	NA	NA	NA
Phosphatidylcholine (18:0_18:2) levels	GCST90277300	0.089	0.031	0.062	12.278	4	0.015	34.633	0.146	NA
Phosphatidylcholine (18:0_20:3) levels	GCST90277303	-0.021	0.099	0.864	5.123	2	0.077	NA	NA	NA
Phosphatidylcholine (18:0_20:4) levels	GCST90277304	-0.013	0.020	0.541	10.217	6	0.116	15.481	0.362	NA
Phosphatidylcholine (18:0_20:5) levels	GCST90277305	NA	NA	NA	0.066	1	0.798	NA	NA	NA
Phosphatidylcholine (18:0_22:5) levels	GCST90277306	NA	NA	NA	6.189	1	0.013	NA	NA	NA
Phosphatidylcholine (18:0_22:6) levels	GCST90277307	NA	NA	NA	6.960	1	0.008	NA	NA	NA
Phosphatidylcholine (18:1_18:1) levels	GCST90277308	0.103	0.033	0.087	11.074	3	0.011	31.435	0.144	NA
Phosphatidylcholine (18:1_18:2) levels	GCST90277309	0.048	0.016	0.060	9.898	4	0.042	42.378	0.303	NA
Phosphatidylcholine (18:1_20:2) levels	GCST90277311	0.002	0.028	0.959	0.229	2	0.892	NA	NA	NA
Phosphatidylcholine (18:1_20:3) levels	GCST90277312	0.131	0.079	0.344	4.390	2	0.111	NA	NA	NA
Phosphatidylcholine (18:1_20:4) levels	GCST90277313	-0.023	0.019	0.266	5.726	6	0.455	17.993	0.433	NA
Phosphatidylcholine (18:2_18:2) levels	GCST90277314	0.089	0.030	0.203	9.412	2	0.009	NA	NA	NA
Phosphatidylcholine (18:2_20:4) levels	GCST90277317	-0.118	0.044	0.115	9.053	3	0.029	22.996	0.158	NA
Phosphatidylcholine (O-16:0_16:1) levels	GCST90277319	NA	NA	NA	1.503	1	0.220	NA	NA	NA
Phosphatidylcholine (O-16:0_18:1) levels	GCST90277320	-0.043	0.096	0.732	0.200	2	0.905	NA	NA	NA
Phosphatidylcholine (O-16:0_18:2) levels	GCST90277321	NA	NA	NA	9.536	1	0.002	NA	NA	NA
Phosphatidylcholine (O-16:0_20:3) levels	GCST90277322	0.118	0.057	0.286	4.342	2	0.114	NA	NA	NA
Phosphatidylcholine (O-16:0_20:4) levels	GCST90277323	-0.038	0.020	0.312	3.509	2	0.173	NA	NA	NA
Phosphatidylcholine (O-16:1_18:1) levels	GCST90277327	-0.007	0.062	0.927	0.576	2	0.750	NA	NA	NA
Phosphatidylcholine (O-16:1_18:2) levels	GCST90277328	NA	NA	NA	0.272	1	0.602	NA	NA	NA
Phosphatidylcholine (O-16:1_20:3) levels	GCST90277329	0.021	0.123	0.890	5.846	2	0.054	NA	NA	NA
Phosphatidylcholine (O-16:1_20:4) levels	GCST90277330	-0.047	0.048	0.504	9.686	2	0.008	NA	NA	NA
Phosphatidylcholine (O-16:2_18:0) levels	GCST90277331	NA	NA	NA	0.067	1	0.795	NA	NA	NA
Phosphatidylcholine (O-18:0_20:4) levels	GCST90277336	NA	NA	NA	0.133	1	0.716	NA	NA	NA
Phosphatidylcholine (O-18:1_16:0) levels	GCST90277337	0.009	0.080	0.926	0.040	2	0.980	NA	NA	NA
Phosphatidylcholine (O-18:1_20:3) levels	GCST90277339	NA	NA	NA	9.466	1	0.002	NA	NA	NA
Phosphatidylcholine (O-18:1_20:4) levels	GCST90277340	-0.016	0.021	0.531	0.659	3	0.883	2.898	0.748	NA
Phosphatidylcholine (O-18:2_16:0) levels	GCST90277341	NA	NA	NA	10.022	1	0.002	NA	NA	NA
Phosphatidylcholine (O-18:2_18:2) levels	GCST90277343	NA	NA	NA	10.546	1	0.001	NA	NA	NA
Phosphatidylcholine (O-18:2_20:4) levels	GCST90277344	NA	NA	NA	11.943	1	0.001	NA	NA	NA
Phosphatidylethanolamine (16:0_18:2) levels	GCST90277345	-0.067	0.041	0.239	7.362	3	0.061	17.933	0.253	rs1077834,rs174564
Phosphatidylethanolamine (16:0_20:4) levels	GCST90277346	0.003	0.022	0.883	2.104	4	0.717	2.697	0.790	NA
Phosphatidylethanolamine (18:0_18:2) levels	GCST90277347	-0.022	0.020	0.302	3.726	8	0.881	4.507	0.891	rs174567
Phosphatidylethanolamine (18:0_20:4) levels	GCST90277348	0.003	0.039	0.946	16.612	7	0.020	20.915	0.051	NA
Phosphatidylethanolamine (18:1_18:1) levels	GCST90277349	-0.037	0.056	0.555	9.475	4	0.050	17.225	0.154	rs174548,rs2070895
Phosphatidylethanolamine (O-16:1_20:4) levels	GCST90277351	NA	NA	NA	9.930	1	0.002	NA	NA	NA
Phosphatidylethanolamine (O-18:1_20:4) levels	GCST90277354	NA	NA	NA	2.174	1	0.140	NA	NA	NA
Phosphatidylethanolamine (O-18:2_20:4) levels	GCST90277357	NA	NA	NA	4.366	1	0.037	NA	NA	NA
Phosphatidylinositol (16:0_18:1) levels	GCST90277358	NA	NA	NA	13.090	1	2.97E-04	NA	NA	NA
Phosphatidylinositol (16:0_18:2) levels	GCST90277359	-0.081	0.126	0.637	15.492	2	4.33E-04	NA	NA	NA
Phosphatidylinositol (16:0_20:4) levels	GCST90277360	-0.404	0.101	0.156	15.915	2	3.50E-04	NA	NA	NA

Phosphatidylinositol (18:0_18:1) levels	GCST90277361	-0.050	0.054	0.418	1.257	4	0.869	2.537	0.835	rs174556
Phosphatidylinositol (18:0_18:2) levels	GCST90277362	0.016	0.038	0.684	17.614	6	0.007	33.089	0.069	NA
Phosphatidylinositol (18:0_20:3) levels	GCST90277363	0.069	0.024	0.061	10.987	4	0.027	27.320	0.179	NA
Phosphatidylinositol (18:0_20:4) levels	GCST90277364	-0.020	0.028	0.526	4.455	5	0.486	5.257	0.625	rs174533
Phosphatidylinositol (18:1_18:1) levels	GCST90277365	0.027	0.373	0.954	0.008	2	0.996	NA	NA	rs174568,rs36634
Phosphatidylinositol (18:1_18:2) levels	GCST90277366	0.269	0.123	0.272	9.827	2	0.007	NA	NA	NA
Phosphatidylinositol (18:1_20:4) levels	GCST90277367	NA	NA	NA	0.182	1	0.670	NA	NA	NA
Sphingomyelin (d32:1) levels	GCST90277368	0.002	0.041	0.976	3.127	2	0.209	NA	NA	NA
Sphingomyelin (d34:0) levels	GCST90277369	0.016	0.088	0.876	6.848	3	0.077	13.293	0.261	NA
Sphingomyelin (d34:1) levels	GCST90277370	-0.005	0.039	0.900	9.611	6	0.142	14.177	0.146	NA
Sphingomyelin (d34:2) levels	GCST90277371	-0.027	0.074	0.742	11.540	4	0.021	20.824	0.073	NA
Sphingomyelin (d36:1) levels	GCST90277372	-0.043	0.025	0.132	10.393	8	0.239	12.586	0.270	NA
Sphingomyelin (d36:2) levels	GCST90277373	-0.094	0.090	0.406	17.936	3	0.000	37.872	0.054	NA
Sphingomyelin (d38:1) levels	GCST90277374	-0.036	0.023	0.158	8.664	9	0.469	9.831	0.557	NA
Sphingomyelin (d38:2) levels	GCST90277375	-0.053	0.028	0.154	4.988	4	0.288	8.148	0.384	rs174556
Sphingomyelin (d40:1) levels	GCST90277376	-0.018	0.030	0.573	7.665	7	0.363	9.316	0.424	NA
Sphingomyelin (d40:2) levels	GCST90277377	-0.018	0.039	0.673	6.943	5	0.225	8.492	0.370	rs174561
Sphingomyelin (d42:2) levels	GCST90277378	0.007	0.033	0.844	7.448	8	0.489	9.496	0.519	NA
Triacylglycerol (48:1) levels	GCST90277382	NA	NA	NA	0.105	1	0.745	NA	NA	NA
Triacylglycerol (48:2) levels	GCST90277383	NA	NA	NA	0.085	1	0.771	NA	NA	NA
Triacylglycerol (48:3) levels	GCST90277384	NA	NA	NA	0.127	1	0.721	NA	NA	NA
Triacylglycerol (49:2) levels	GCST90277386	NA	NA	NA	0.182	1	0.670	NA	NA	NA
Triacylglycerol (50:1) levels	GCST90277387	-0.017	0.057	0.816	0.280	2	0.869	NA	NA	NA
Triacylglycerol (50:2) levels	GCST90277388	NA	NA	NA	0.198	1	0.657	NA	NA	NA
Triacylglycerol (50:3) levels	GCST90277389	-0.047	0.087	0.687	0.287	2	0.866	NA	NA	NA
Triacylglycerol (50:4) levels	GCST90277390	-0.050	0.103	0.712	0.248	2	0.884	NA	NA	NA
Triacylglycerol (50:5) levels	GCST90277391	-0.038	0.073	0.696	0.302	2	0.860	NA	NA	NA
Triacylglycerol (51:2) levels	GCST90277393	NA	NA	NA	0.339	1	0.560	NA	NA	NA
Triacylglycerol (51:3) levels	GCST90277394	0.001	0.029	0.976	2.885	5	0.718	3.771	0.766	NA
Triacylglycerol (51:4) levels	GCST90277395	NA	NA	NA	0.388	1	0.533	NA	NA	NA
Triacylglycerol (52:2) levels	GCST90277396	0.008	0.037	0.850	2.599	3	0.458	3.708	0.570	NA
Triacylglycerol (52:3) levels	GCST90277397	-0.018	0.024	0.486	3.617	5	0.606	5.081	0.631	NA
Triacylglycerol (52:4) levels	GCST90277398	-0.021	0.025	0.443	4.261	6	0.641	5.724	0.682	NA
Triacylglycerol (52:5) levels	GCST90277399	0.017	0.037	0.682	1.552	3	0.670	2.432	0.740	NA
Triacylglycerol (52:6) levels	GCST90277400	-0.031	0.051	0.649	0.398	2	0.820	NA	NA	rs102274
Triacylglycerol (53:2) levels	GCST90277401	NA	NA	NA	0.456	1	0.500	NA	NA	NA
Triacylglycerol (53:3) levels	GCST90277402	0.027	0.038	0.535	6.457	4	0.168	8.670	0.271	rs174560
Triacylglycerol (53:4) levels	GCST90277403	0.010	0.030	0.757	1.742	3	0.628	2.588	0.720	NA
Triacylglycerol (54:3) levels	GCST90277404	0.042	0.029	0.382	2.357	2	0.308	NA	NA	rs174560
Triacylglycerol (54:4) levels	GCST90277405	-0.022	0.032	0.543	12.309	5	0.031	15.597	0.091	NA
Triacylglycerol (54:5) levels	GCST90277406	0.016	0.041	0.729	3.987	3	0.263	6.088	0.400	NA
Triacylglycerol (54:6) levels	GCST90277407	0.083	0.121	0.616	19.006	2	7.46E-05	NA	NA	NA
Triacylglycerol (54:7) levels	GCST90277408	-0.030	0.046	0.627	0.455	2	0.797	NA	NA	rs174584
Triacylglycerol (56:3) levels	GCST90277409	NA	NA	NA	0.037	1	0.848	NA	NA	rs174560,rs1759614
Triacylglycerol (56:4) levels	GCST90277410	-0.079	0.185	0.743	17.456	2	1.62E-04	NA	NA	NA
Triacylglycerol (56:5) levels	GCST90277411	0.044	0.064	0.616	1.114	2	0.573	NA	NA	rs97384
Triacylglycerol (56:6) levels	GCST90277412	-0.004	0.032	0.917	2.054	5	0.842	2.698	0.872	rs174533
Triacylglycerol (56:7) levels	GCST90277413	-0.007	0.032	0.844	1.818	3	0.611	2.736	0.701	rs174554
Triacylglycerol (56:8) levels	GCST90277414	0.044	0.043	0.407	2.762	3	0.430	4.786	0.500	rs174530
Triacylglycerol (58:7) levels	GCST90277415	-0.001	0.035	0.978	2.583	4	0.630	3.807	0.694	rs102275
Triacylglycerol (58:8) levels	GCST90277416	NA	NA	NA	0.009	1	0.926	NA	NA	NA

Table S8.Summary of the results of the reverse Mendelian randomization analysis

outcome	exposure	method	nsnp	b	se	pval	lo_ci	up_ci
Sterol ester (27:1/14:0) levels	CRC	IVW	6	0.011449	0.056447	0.839268	-0.099187	0.122085
Sterol ester (27:1/15:0) levels	CRC	IVW	6	-0.03347	0.057499	0.560496	-0.146168	0.079227
Sterol ester (27:1/16:0) levels	CRC	IVW	7	0.009265	0.050192	0.853545	-0.08911	0.107641
Sterol ester (27:1/16:1) levels	CRC	IVW	8	-0.028445	0.049513	0.565634	-0.12549	0.068601
Sterol ester (27:1/17:0) levels	CRC	IVW	7	0.001804	0.051314	0.971948	-0.09877	0.102379
Sterol ester (27:1/17:1) levels	CRC	IVW	7	0.011419	0.05287	0.829001	-0.092207	0.115045
Sterol ester (27:1/18:0) levels	CRC	IVW	8	0.022927	0.04795	0.632555	-0.071056	0.116909
Sterol ester (27:1/18:1) levels	CRC	IVW	6	0.01583	0.05607	0.777693	-0.094067	0.125727
Sterol ester (27:1/18:2) levels	CRC	IVW	7	0.029567	0.050306	0.556709	-0.069034	0.128168
Sterol ester (27:1/18:3) levels	CRC	IVW	5	-0.010464	0.061959	0.865882	-0.131904	0.110975
Sterol ester (27:1/20:2) levels	CRC	IVW	8	-0.013781	0.053579	0.797015	-0.118795	0.091233
Sterol ester (27:1/20:3) levels	CRC	IVW	6	-0.016873	0.05199	0.745524	-0.118773	0.085027
Sterol ester (27:1/20:4) levels	CRC	IVW	9	-0.011502	0.044569	0.796361	-0.098857	0.075854
Sterol ester (27:1/20:5) levels	CRC	IVW	6	0.016254	0.052961	0.758911	-0.08755	0.120059
Sterol ester (27:1/22:6) levels	CRC	IVW	6	0.02035	0.052921	0.700587	-0.083376	0.124075
Ceramide (d40:1) levels	CRC	IVW	7	0.048267	0.052463	0.357567	-0.054561	0.151095
Ceramide (d40:2) levels	CRC	IVW	4	0.021552	0.069943	0.757981	-0.115536	0.158639
Ceramide (d42:1) levels	CRC	IVW	7	0.026186	0.0498	0.599011	-0.071422	0.123794
Ceramide (d42:2) levels	CRC	IVW	8	-0.028099	0.050115	0.575013	-0.126325	0.070127
Cholesterol levels	CRC	IVW	9	0.015042	0.045117	0.73884	-0.073388	0.103471
Diacylglycerol (16:0_18:1) levels	CRC	IVW	6	-0.026629	0.05801	0.646209	-0.140329	0.087071
Diacylglycerol (16:0_18:2) levels	CRC	IVW	6	-0.018454	0.060957	0.76209	-0.137931	0.101023
Diacylglycerol (16:1_18:1) levels	CRC	IVW	7	-0.033499	0.05439	0.537956	-0.140104	0.073105
Diacylglycerol (18:1_18:1) levels	CRC	IVW	6	-0.044538	0.055347	0.420983	-0.153018	0.063941
Diacylglycerol (18:1_18:2) levels	CRC	IVW	5	-0.087809	0.063754	0.168413	-0.212766	0.037148
Diacylglycerol (18:1_18:3) levels	CRC	IVW	8	0.002613	0.05038	0.95864	-0.096132	0.101357
Phosphatidylcholine (16:0_0:0) levels	CRC	IVW	7	-0.027091	0.052124	0.603241	-0.129254	0.075072
Phosphatidylcholine (18:0_0:0) levels	CRC	IVW	6	0.049922	0.056965	0.380836	-0.06173	0.161573
Phosphatidylcholine (18:1_0:0) levels	CRC	IVW	8	-0.012832	0.047497	0.787026	-0.105926	0.080261
Phosphatidylcholine (18:2_0:0) levels	CRC	IVW	7	-0.004468	0.052236	0.931833	-0.106852	0.097915
Phosphatidylcholine (20:4_0:0) levels	CRC	IVW	10	-0.016565	0.04595	0.718466	-0.106626	0.073496
Phosphatidylethanolamine (18:0_0:0) levels	CRC	IVW	8	-0.000632	0.053365	0.990552	-0.105228	0.103964
Phosphatidylethanolamine (18:1_0:0) levels	CRC	IVW	7	0.013755	0.057736	0.811702	-0.099408	0.126917
Phosphatidylethanolamine (18:2_0:0) levels	CRC	IVW	9	-0.008651	0.045848	0.850339	-0.098513	0.081211
Phosphatidylcholine (14:0_16:0) levels	CRC	IVW	8	0.046783	0.054178	0.387863	-0.059406	0.152972
Phosphatidylcholine (14:0_18:1) levels	CRC	IVW	8	-0.028468	0.049632	0.566251	-0.125746	0.068811
Phosphatidylcholine (14:0_18:2) levels	CRC	IVW	7	-0.044022	0.051641	0.39396	-0.145238	0.057194
Phosphatidylcholine (15:0_18:1) levels	CRC	IVW	8	-0.009351	0.049606	0.85048	-0.106578	0.087876
Phosphatidylcholine (15:0_18:2) levels	CRC	IVW	6	0.099849	0.053841	0.063666	-0.00568	0.205377
Phosphatidylcholine (16:0_16:0) levels	CRC	IVW	10	0.034884	0.042976	0.41696	-0.04935	0.119118
Phosphatidylcholine (16:0_16:1) levels	CRC	IVW	8	-0.029756	0.047142	0.527913	-0.122154	0.062642
Phosphatidylcholine (16:0_17:1) levels	CRC	IVW	10	-0.00373	0.043317	0.931378	-0.088631	0.081171
Phosphatidylcholine (16:0_18:0) levels	CRC	IVW	8	0.039372	0.046865	0.400845	-0.052483	0.131226
Phosphatidylcholine (16:0_18:1) levels	CRC	IVW	9	-0.036235	0.045398	0.424776	-0.125216	0.052745
Phosphatidylcholine (16:0_18:2) levels	CRC	IVW	6	0.076497	0.052127	0.142236	-0.025672	0.178665
Phosphatidylcholine (16:0_18:3) levels	CRC	IVW	7	-0.048124	0.049863	0.334485	-0.145856	0.049608
Phosphatidylcholine (16:0_20:1) levels	CRC	IVW	7	0.03796	0.052189	0.467	-0.064329	0.14025
Phosphatidylcholine (16:0_20:2) levels	CRC	IVW	7	-0.019211	0.049768	0.699482	-0.116756	0.078333
Phosphatidylcholine (16:0_20:3) levels	CRC	IVW	4	0.019306	0.063452	0.760923	-0.105059	0.143672
Phosphatidylcholine (16:0_20:4) levels	CRC	IVW	7	0.02721	0.052018	0.600915	-0.074745	0.129165
Phosphatidylcholine (16:0_20:5) levels	CRC	IVW	5	0.02086	0.060928	0.732067	-0.098559	0.14028
Phosphatidylcholine (16:0_22:4) levels	CRC	IVW	7	-0.049647	0.050172	0.3224	-0.147985	0.04869
Phosphatidylcholine (16:0_22:5) levels	CRC	IVW	7	-0.021958	0.052388	0.675106	-0.124638	0.080721
Phosphatidylcholine (16:0_22:6) levels	CRC	IVW	9	0.030649	0.044822	0.4941	-0.057201	0.118499
Phosphatidylcholine (16:1_18:0) levels	CRC	IVW	7	0.009966	0.05188	0.847662	-0.091719	0.111651
Phosphatidylcholine (16:1_18:1) levels	CRC	IVW	9	-0.027247	0.045117	0.545899	-0.115675	0.061182
Phosphatidylcholine (16:1_18:2) levels	CRC	IVW	8	0.016194	0.047033	0.730622	-0.075992	0.108379
Phosphatidylcholine (16:1_20:4) levels	CRC	IVW	7	0.010815	0.05603	0.846942	-0.099003	0.120633
Phosphatidylcholine (17:0_18:1) levels	CRC	IVW	8	-0.002443	0.047832	0.959264	-0.096194	0.091308
Phosphatidylcholine (17:0_18:2) levels	CRC	IVW	5	0.045802	0.05688	0.420678	-0.065683	0.157287
Phosphatidylcholine (17:0_20:4) levels	CRC	IVW	8	0.037539	0.047078	0.425226	-0.054733	0.129811
Phosphatidylcholine (18:0_18:1) levels	CRC	IVW	9	0.023022	0.045789	0.615124	-0.066725	0.112768
Phosphatidylcholine (18:0_18:2) levels	CRC	IVW	4	0.01445	0.073965	0.845108	-0.13052	0.15942
Phosphatidylcholine (18:0_18:3) levels	CRC	IVW	8	0.03244	0.048089	0.499946	-0.061815	0.126695
Phosphatidylcholine (18:0_20:2) levels	CRC	IVW	6	0.007867	0.058881	0.893711	-0.107539	0.123273
Phosphatidylcholine (18:0_20:3) levels	CRC	IVW	7	0.026345	0.049027	0.591019	-0.069748	0.122439
Phosphatidylcholine (18:0_20:4) levels	CRC	IVW	9	0.031288	0.044392	0.480929	-0.055721	0.118297
Phosphatidylcholine (18:0_20:5) levels	CRC	IVW	7	0.036466	0.050103	0.466719	-0.061735	0.134668
Phosphatidylcholine (18:0_22:5) levels	CRC	IVW	10	0.00225	0.044113	0.95933	-0.084213	0.088712
Phosphatidylcholine (18:0_22:6) levels	CRC	IVW	8	-0.004253	0.048458	0.930068	-0.09923	0.090724
Phosphatidylcholine (18:1_18:1) levels	CRC	IVW	8	-0.006253	0.046812	0.893739	-0.098004	0.085498
Phosphatidylcholine (18:1_18:2) levels	CRC	IVW	7	0.057255	0.048917	0.241826	-0.038623	0.153133
Phosphatidylcholine (18:1_18:3) levels	CRC	IVW	5	0.015322	0.060591	0.800362	-0.103436	0.13408
Phosphatidylcholine (18:1_20:2) levels	CRC	IVW	8	-0.018363	0.053388	0.730878	-0.123004	0.086278
Phosphatidylcholine (18:1_20:3) levels	CRC	IVW	7	0.004837	0.049102	0.921524	-0.091404	0.101078
Phosphatidylcholine (18:1_20:4) levels	CRC	IVW	9	0.015446	0.04472	0.729805	-0.072205	0.103096
Phosphatidylcholine (18:2_18:2) levels	CRC	IVW	5	0.045533	0.057485	0.428308	-0.067137	0.158203
Phosphatidylcholine (18:2_20:1) levels	CRC	IVW	6	0.028432	0.063801	0.655855	-0.096617	0.153482
Phosphatidylcholine (18:2_20:3) levels	CRC	IVW	7	0.043221	0.050893	0.395735	-0.056528	0.142971
Phosphatidylcholine (18:2_20:4) levels	CRC	IVW	7	0.046231	0.049188	0.347285	-0.050179	0.14264
Phosphatidylcholine (O-16:0_16:0) levels	CRC	IVW	6	-0.054525	0.053501	0.308132	-0.159387	0.050336
Phosphatidylcholine (O-16:0_16:1) levels	CRC	IVW	6	-0.026653	0.065607	0.684561	-0.155241	0.101936
Phosphatidylcholine (O-16:0_18:1) levels	CRC	IVW	7	0.003717	0.051544	0.942508	-0.09731	0.104744
Phosphatidylcholine (O-16:0_18:2) levels	CRC	IVW	7	0.046367	0.05287	0.380489	-0.057258	0.149992
Phosphatidylcholine (O-16:0_20:3) levels	CRC	IVW	8	0.025335	0.048204	0.599175	-0.069144	0.119815
Phosphatidylcholine (O-16:0_20:4) levels	CRC	IVW	9	0.052658	0.044917	0.241057	-0.035379	0.140694
Phosphatidylcholine (O-16:0_22:5) levels	CRC	IVW	8	-0.015861	0.051814	0.759525	-0.117417	0.085695
Phosphatidylcholine (O-16:1_16:0) levels	CRC	IVW	7	-0.013964	0.059128	0.813307	-0.129854	0.101926
Phosphatidylcholine (O-16:1_18:0) levels	CRC	IVW	7	-0.019741	0.054668	0.718017	-0.126891	0.087409
Phosphatidylcholine (O-16:1_18:1) levels	CRC	IVW	8	0.020616	0.050008	0.680151	-0.077399	0.118631
Phosphatidylcholine (O-16:1_18:2) levels	CRC	IVW	7	0.012673	0.053551	0.812931	-0.092288	0.117633
Phosphatidylcholine (O-16:1_20:3) levels	CRC	IVW	6	-0.013945	0.052681	0.79124	-0.1172	0.08931
Phosphatidylcholine (O-16:1_20:4) levels	CRC	IVW	8	0.060847	0.050592	0.229095	-0.038314	0.160007
Phosphatidylcholine (O-16:2_18:0) levels	CRC	IVW	9	0.029422	0.046476	0.526692	-0.06167	0.120514
Phosphatidylcholine (O-17:0_15:0) levels	CRC	IVW	6	0.040107	0.056413	0.477106	-0.070461	0.150676
Phosphatidylcholine (O-17:0_17:1) levels	CRC	IVW	5	-0.046999	0.059028	0.425908	-0.162695	0.068696
Phosphatidylcholine (O-18:0_14:0) levels	CRC	IVW	8	0.019555	0.049503	0.692829	-0.077472	0.116581

Phosphatidylcholine (O-18:0_16:1) levels	CRC	IVW	7	0.003226	0.053962	0.952328	-0.102539	0.108991
Phosphatidylcholine (O-18:0_20:4) levels	CRC	IVW	9	0.022008	0.045557	0.629044	-0.067285	0.1113
Phosphatidylcholine (O-18:1_16:0) levels	CRC	IVW	7	0.002552	0.052657	0.961351	-0.100657	0.10576
Phosphatidylcholine (O-18:1_18:2) levels	CRC	IVW	7	-0.009593	0.054892	0.861263	-0.117182	0.097995
Phosphatidylcholine (O-18:1_20:3) levels	CRC	IVW	8	0.02041	0.048379	0.673107	-0.074412	0.115233
Phosphatidylcholine (O-18:1_20:4) levels	CRC	IVW	8	0.025159	0.046652	0.589689	-0.066279	0.116597
Phosphatidylcholine (O-18:2_16:0) levels	CRC	IVW	7	0.005184	0.055445	0.925507	-0.103488	0.113856
Phosphatidylcholine (O-18:2_18:1) levels	CRC	IVW	7	0.04907	0.051512	0.3408	-0.051894	0.150034
Phosphatidylcholine (O-18:2_18:2) levels	CRC	IVW	6	0.009825	0.053541	0.854403	-0.095116	0.114765
Phosphatidylcholine (O-18:2_20:4) levels	CRC	IVW	5	0.001564	0.06115	0.979596	-0.118289	0.121417
Phosphatidylethanolamine (16:0_18:2) levels	CRC	IVW	6	-0.007349	0.064411	0.909159	-0.133594	0.118896
Phosphatidylethanolamine (16:0_20:4) levels	CRC	IVW	5	0.058386	0.063574	0.358409	-0.066218	0.18299
Phosphatidylethanolamine (18:0_18:2) levels	CRC	IVW	6	0.001873	0.052729	0.971659	-0.101476	0.105223
Phosphatidylethanolamine (18:0_20:4) levels	CRC	IVW	5	-0.002029	0.061209	0.973556	-0.122	0.117942
Phosphatidylethanolamine (18:1_18:1) levels	CRC	IVW	8	-0.029063	0.04912	0.554065	-0.125339	0.067212
Phosphatidylethanolamine (O-16:1_18:2) leve	CRC	IVW	7	0.033988	0.062823	0.588502	-0.089145	0.15712
Phosphatidylethanolamine (O-16:1_20:4) leve	CRC	IVW	4	0.064906	0.063232	0.30467	-0.059029	0.188841
Phosphatidylethanolamine (O-16:1_22:5) leve	CRC	IVW	6	-0.00329	0.064447	0.959284	-0.129606	0.123026
Phosphatidylethanolamine (O-18:1_18:2) leve	CRC	IVW	9	0.027695	0.046815	0.554123	-0.064062	0.119453
Phosphatidylethanolamine (O-18:1_20:4) leve	CRC	IVW	8	0.005815	0.04997	0.907365	-0.092126	0.103755
Phosphatidylethanolamine (O-18:2_18:1) leve	CRC	IVW	5	-0.048458	0.06231	0.436752	-0.170585	0.07367
Phosphatidylethanolamine (O-18:2_18:2) leve	CRC	IVW	7	0.026388	0.05283	0.617442	-0.07716	0.129935
Phosphatidylethanolamine (O-18:2_20:4) leve	CRC	IVW	7	0.011629	0.050601	0.818235	-0.08755	0.110808
Phosphatidylinositol (16:0_18:1) levels	CRC	IVW	7	0.042791	0.054697	0.434016	-0.064414	0.149997
Phosphatidylinositol (16:0_18:2) levels	CRC	IVW	5	0.063142	0.066002	0.338737	-0.066222	0.192506
Phosphatidylinositol (16:0_20:4) levels	CRC	IVW	8	0.056352	0.052717	0.285092	-0.046974	0.159678
Phosphatidylinositol (18:0_18:1) levels	CRC	IVW	9	0.021745	0.047444	0.646711	-0.071245	0.114736
Phosphatidylinositol (18:0_18:2) levels	CRC	IVW	5	0.018381	0.066612	0.782599	-0.112179	0.148941
Phosphatidylinositol (18:0_20:3) levels	CRC	IVW	8	-0.012419	0.048326	0.797193	-0.107138	0.0823
Phosphatidylinositol (18:0_20:4) levels	CRC	IVW	6	0.035701	0.054359	0.511336	-0.070843	0.142245
Phosphatidylinositol (18:1_18:1) levels	CRC	IVW	8	0.011008	0.050185	0.826375	-0.087355	0.109371
Phosphatidylinositol (18:1_18:2) levels	CRC	IVW	9	0.033391	0.046954	0.476993	-0.058639	0.125422
Phosphatidylinositol (18:1_20:4) levels	CRC	IVW	6	0.044187	0.062192	0.477402	-0.07771	0.166083
Sphingomyelin (d32:1) levels	CRC	IVW	6	0.025492	0.058677	0.663967	-0.089515	0.140499
Sphingomyelin (d34:0) levels	CRC	IVW	7	0.056643	0.053147	0.286529	-0.047526	0.160812
Sphingomyelin (d34:1) levels	CRC	IVW	7	0.028225	0.051021	0.580126	-0.071776	0.128226
Sphingomyelin (d34:2) levels	CRC	IVW	8	-0.001663	0.050436	0.973692	-0.100518	0.097192
Sphingomyelin (d36:1) levels	CRC	IVW	4	-0.022145	0.062968	0.725077	-0.145563	0.101273
Sphingomyelin (d36:2) levels	CRC	IVW	6	0.028836	0.055673	0.604492	-0.080284	0.137956
Sphingomyelin (d38:1) levels	CRC	IVW	7	-0.020304	0.050968	0.690356	-0.120201	0.079593
Sphingomyelin (d38:2) levels	CRC	IVW	4	-0.016205	0.063089	0.797282	-0.139859	0.107449
Sphingomyelin (d40:1) levels	CRC	IVW	5	-0.044746	0.057311	0.434943	-0.157077	0.067584
Sphingomyelin (d40:2) levels	CRC	IVW	7	0.032378	0.051001	0.525518	-0.067583	0.13234
Sphingomyelin (d42:2) levels	CRC	IVW	5	0.049246	0.057315	0.390215	-0.06309	0.161583
Triacylglycerol (46:1) levels	CRC	IVW	4	-0.016025	0.068135	0.814054	-0.149569	0.117519
Triacylglycerol (46:2) levels	CRC	IVW	6	0.045123	0.064328	0.483022	-0.08096	0.171205
Triacylglycerol (48:0) levels	CRC	IVW	4	0.058987	0.075062	0.431963	-0.088135	0.206108
Triacylglycerol (48:1) levels	CRC	IVW	4	-0.043356	0.062606	0.488612	-0.166064	0.079352
Triacylglycerol (48:2) levels	CRC	IVW	6	-0.065665	0.054323	0.226742	-0.172138	0.040808
Triacylglycerol (48:3) levels	CRC	IVW	6	-0.022737	0.054383	0.675884	-0.129327	0.083853
Triacylglycerol (49:1) levels	CRC	IVW	7	-0.028303	0.056854	0.618617	-0.139736	0.083131
Triacylglycerol (49:2) levels	CRC	IVW	6	0.010903	0.062949	0.862485	-0.112476	0.134283
Triacylglycerol (50:1) levels	CRC	IVW	6	-0.03044	0.053293	0.567875	-0.134893	0.074014
Triacylglycerol (50:2) levels	CRC	IVW	6	-0.045648	0.053167	0.390578	-0.149856	0.05856
Triacylglycerol (50:3) levels	CRC	IVW	5	-0.007671	0.060212	0.898628	-0.125685	0.110344
Triacylglycerol (50:4) levels	CRC	IVW	5	0.004347	0.060254	0.94249	-0.113751	0.122444
Triacylglycerol (50:5) levels	CRC	IVW	4	-0.020601	0.070146	0.769	-0.158086	0.116885
Triacylglycerol (51:1) levels	CRC	IVW	5	0.020214	0.066891	0.762501	-0.110892	0.151321
Triacylglycerol (51:2) levels	CRC	IVW	6	-0.059993	0.053237	0.259784	-0.164338	0.044352
Triacylglycerol (51:3) levels	CRC	IVW	7	-0.056709	0.050825	0.264524	-0.156327	0.042909
Triacylglycerol (51:4) levels	CRC	IVW	7	0.05106	0.056323	0.364635	-0.059332	0.161453
Triacylglycerol (52:2) levels	CRC	IVW	6	-0.043088	0.053172	0.41774	-0.147306	0.06113
Triacylglycerol (52:3) levels	CRC	IVW	6	-0.041246	0.053117	0.437445	-0.145356	0.062864
Triacylglycerol (52:4) levels	CRC	IVW	5	-0.00602	0.060118	0.92024	-0.123852	0.111812
Triacylglycerol (52:5) levels	CRC	IVW	4	-0.008273	0.065199	0.899027	-0.136062	0.119516
Triacylglycerol (52:6) levels	CRC	IVW	5	0.069933	0.061359	0.254398	-0.050331	0.190196
Triacylglycerol (53:2) levels	CRC	IVW	5	-0.061911	0.05799	0.285694	-0.17557	0.051749
Triacylglycerol (53:3) levels	CRC	IVW	5	-0.025205	0.060318	0.676043	-0.143429	0.093019
Triacylglycerol (53:4) levels	CRC	IVW	7	-0.029566	0.053355	0.579483	-0.134143	0.07501
Triacylglycerol (54:3) levels	CRC	IVW	7	-0.002145	0.053717	0.968149	-0.107429	0.10314
Triacylglycerol (54:4) levels	CRC	IVW	8	0.000488	0.047478	0.991791	-0.092569	0.093546
Triacylglycerol (54:5) levels	CRC	IVW	7	0.017476	0.052328	0.738402	-0.085087	0.120038
Triacylglycerol (54:6) levels	CRC	IVW	6	-0.014557	0.052581	0.781895	-0.117615	0.088501
Triacylglycerol (54:7) levels	CRC	IVW	8	-0.027391	0.04758	0.564829	-0.120649	0.065866
Triacylglycerol (56:3) levels	CRC	IVW	6	0.01944	0.057633	0.735884	-0.093521	0.132401
Triacylglycerol (56:4) levels	CRC	IVW	5	0.011419	0.062745	0.855585	-0.111561	0.1344
Triacylglycerol (56:5) levels	CRC	IVW	6	-0.004733	0.064605	0.941593	-0.13136	0.121893
Triacylglycerol (56:6) levels	CRC	IVW	6	-0.007388	0.055204	0.893539	-0.115588	0.100812
Triacylglycerol (56:7) levels	CRC	IVW	6	-0.038413	0.055098	0.485688	-0.146404	0.069578
Triacylglycerol (56:8) levels	CRC	IVW	6	-0.032778	0.056511	0.561893	-0.14354	0.077984
Triacylglycerol (58:7) levels	CRC	IVW	5	-0.018134	0.069637	0.794552	-0.154623	0.118355
Triacylglycerol (58:8) levels	CRC	IVW	4	-0.023398	0.06718	0.727625	-0.15507	0.108274

These are selected and significantly reduced contents. Showing results for the main method only. Readers may request the nonmodified whole contents by contacting the corresponding authors of the current study: weiguoli1987@163.com (G.W.); Huojiege@jsatcm.com (J. H.)

Table S9. Summary of co-localization analysis results

phenotype	nsnps	PP.H0.abf	PP.H1.abf	PP.H2.abf	PP.H3.abf	PP.H4.abf
Sterol ester (27:1/20:4) levels	210	2.17E-253	0.230656	9.52E-255	0.00937	76.00%
Sterol ester (27:1/20:5) levels	298	7.15E-128	0.010232	2.15E-127	0.029828	95.99%
Phosphatidylcholine (20:4_0:0) levels	269	4.71E-195	0.014161	1.56E-194	0.045902	93.99%
Phosphatidylcholine (16:0_18:0) levels	320	5.48E-14	0.020803	1.22E-13	0.045351	93.38%
Phosphatidylcholine (16:0_22:4) levels	258	9.24E-59	0.017916	3.02E-58	0.057584	92.45%
Phosphatidylcholine (16:0_22:5) levels	244	1.70E-129	0.017907	4.09E-129	0.042247	93.98%
Phosphatidylcholine (18:0_20:5) levels	256	1.38E-131	0.012185	4.11E-131	0.035355	95.25%
Phosphatidylcholine (18:1_20:2) levels	298	4.20E-117	0.009734	1.27E-116	0.028512	96.18%
Phosphatidylcholine (O-18:1_18:2) levels	258	3.64E-35	0.0162	1.19E-34	0.051973	93.18%
Phosphatidylethanolamine (18:2_0:0) levels	275	8.44E-93	0.007631	2.79E-92	0.024285	96.81%
